# Global, regional, and national burdens of pancreatic cancer attributable to smoking from 1990 to 2021 and the projections to 2035:a systematic analysis from the global burden of disease study 2021

**DOI:** 10.3389/fonc.2025.1547029

**Published:** 2025-05-30

**Authors:** Roubing Du, Yu Wang, Mengshu Pan, Jie Zhu, Yaping Zhao, Chuanzhen Zhang, Changhong Liu, Yuan Gao

**Affiliations:** ^1^ Department of Gastroenterology, The First Affiliated Hospital of Shandong First Medical University & Shandong Provincial Qianfoshan Hospital, Jinan, Shandong, China; ^2^ Primary Care Medicine Department, Second Affiliated Hospital of Anhui Medical University, Hefei, Anhui, China; ^3^ Department of Infectious Disease, Second Affiliated Hospital of Anhui Medical University, Hefei, Anhui, China; ^4^ Shandong University of Traditional Chinese Medicine, Jinan, Shandong, China

**Keywords:** global health, smoking, pancreatic cancer, disability-adjusted life years, average annual percentage changes

## Abstract

**Background:**

Studies on global epidemiological patterns regarding the burden of pancreatic cancer (PC) attributable to smoking are limited. This study aimed to analyze the latest disease burden of PC attributable to smoking between 1990 and 2021, further analyze the main factors, and predict the trend in the coming period.

**Methods:**

Data from the Global Burden of Diseases, Injuries, and Risk Factors Study (GBD) 2021 was extracted and analyzed by different geographical levels, age, sex, and socio-demographic index (SDI). Key measures included age-standardized disability-adjusted life years (DALYs) rates (ASDR), age-standardized mortality rates (ASMR), and average annual percentage changes (AAPCs). Further analyses were conducted using the decomposition analysis and Bayesian Age-Period-Cohort (BAPC) model.

**Results:**

Globally, the ASDR and ASMR of the disease demonstrated a decreasing trend. The burden was significantly higher among males, the middle-aged, and the elderly population. A positive correlation with SDI levels across most regions was exhibited. Join-point analysis indicated a decreasing trend in disease burden among high SDI regions while an increasing trend among low-middle SDI regions. Decomposition analysis indicated that proactive epidemiological changes had played a positive role in reducing the burden in High SDI regions. Projection analysis estimated that the disease burden in East Asia, showing a significant upward trend, warranted particular focus.

**Conclusions:**

Despite ongoing tobacco control efforts, the global burden of the disease, which has declined only slightly, remains a significant concern, particularly in high-income areas and among men. Based on stronger tobacco control measures, increased emphasis on disease prevention, early screening, and treatment is essential.

## Highlights

- This study comprehensively documents the global burden of PC attributable to smoking using the latest GBD 2021 database as well as projects the trend in the coming period.
- Despite the global decline in smoking rates, the burden of PC attributable to smoking has not decreased significantly.
- Greater consideration should be given to high-income countries and low- and middle-income countries, especially male populations, in the design and enhancement of global planning for tobacco control and health promotion.

## Introduction

Pancreatic cancer (PC) represents a group of highly aggressive malignant tumors of the digestive system originating from pancreatic duct epithelial cells and acinar cells (1). The disease typically presents with an insidious onset, making early diagnosis difficult. Patients often lack opportunities for curative surgery and show poor tolerance to conventional cancer treatments, resulting in an abysmal prognosis. s (2),. According to data from the Surveillance, Epidemiology, and End Results project database of the National Cancer Institute’s data for PC in both sexes and all races, during 2014-2020, more than half (51%) of all cases were diagnosed at the distant stage with a 5-year survival rate of 3.1%, and even taking all the stages into account, the 5-year survival ratesare only 12.8% (3). According to the latest estimate based on global cancer statistics by world region for the year 2022 from the International Agency for Research on Cancer, this disease ranked as the sixth leading cause of cancer mortality, accounting for nearly 5% of all cancer deaths globally (4). The GBD 2021 reveals that the number of incident and death cases of PC in both sexes increased more than one time from 207905 and 211613 to 508532 and 505752 (5). All these reports have indicated a gradual increase in the number of incident cases and deaths from PC, which would not only pose a serious threat to human life and health but also constitute a significant burden on public health.

According to the most recent overview of the established modifiable risk factors for PC, cigarette smoking, obesity, alcohol use, new-onset diabetes, long-standing diabetes, family history of PC, and pancreatitis were the well-established risks factors for PC, with cigarette smoking remaining a leading cause worldwide (6). A uniquely large collaborative pooled analysis analyzing data from the International PC Case–Control Consortium, confirmed that compared with never smokers, the OR was 1.2 (95% confidence interval [CI] 1.0 to 1.3) for former smokers and 2.2 (95% CI 1.7 to 2.8) for current cigarette smokers, with a significant increasing trend in risk with increasing number of cigarettes among current smokers (odds ratios [OR] = 3.4 for ≥ 35 cigarettes per day, P for trend <0.0001) and duration of cigarette smoking (OR = 2.4 for ≥ 40years of smoking) (7). In addition to being a high-risk factor for the incidence, cigarette smoking has also been associated with a reduction in survival among patients with PC. Through survival analysis, Chen Yuan et al. observed that compared to never-smokers, the multivariable-adjusted hazard ratio (HR) for death in current smokers was 1.37 (95% CI, 1.11 to 1.69, P = 0.003). Additionally, as the number of pack-years of smoking increased, the survival rate showed a statistically significant negative correlation (P trend =0.008) a (8). In addition, tobacco exposure has been shown through basic experimental studies to initiate and accelerate the onset and progression of PC at the mechanistic level (9). In addition, recent basic research uncovered that cigarette smoke extract (CSE) exposure can not only significantly promote PC attributable to smoking progression by upregulating of Chromobox protein homolog 3 (CBX3) via promoting the overexpression of Y-box-binding protein 1 (YBX1) but also the invasion and metastasis of the cancer cells by inducing histone deacetylase 4 (HDAC4) (10, 11).

Through a 15-year period joint effect of the WHO Framework Convention on Tobacco Control (WHO FCTC) together with its technical package–MPOWER has reduced the global prevalence of smoking from 22.8% in 2007 to 17.0% in 2021, more than 8 million people still die each year from tobacco-related diseases (12). Smoking prevalence varies widely by region, age group, and sex, potentially affecting the distribution of PC attributable to smoking. However, studies on the global epidemiological patterns regarding the burden of PC attributable to smoking are relatively limited. In our study, for the first time, we used the latest data from the publicly available Global Burden of Disease database 2021 to analyze the distribution and changes in the global burden of PC attributable to smoking between 1990 and 2021 (13). Unlike previous studies that primarily focused on specific regions or diseases, our analysis not only covers the global burden of PC but also takes into account differences across regions, age groups, genders, and SDI levels. Additionally, this study delves into the impact of changes in age structure, population growth, and epidemiological trends on disease burden in different regions, and provides predictions for future trends. This offers new perspectives and scientific evidence for developing more targeted and cost-effective tobacco control policies.

## Method

### Data source

Estimating years lived with disability (YLDs), years of life lost (YLLs), disability-adjusted life-years (DALYs), and healthy life expectancy (HALE) for 371 diseases and injuries in seven super-regions, 21 regions, 204 countries and territories (including 21 countries with subnational locations), and 811 subnational locations, from 1990 to 2021, the Global Burden of Diseases, Injuries, and Risk Factors Study (GBD) 2021 aims to provide comprehensive and current global, regional, and national data on the burden of disease, injury, and risk factors to quantify the state of global health. These data were stratified by geographical location, socio-economic development level, gender, and different age groups (5). Based on 2021 Socio-demographic Index (SDI) values, a composite indicator of background social and economic conditions that influence health outcomes in each location, countries were divided into five quintiles, termed low, low-middle, middle, high-middle, and high. The SDI values for each country and their corresponding classifications can be accessed from the Institute for Health Metrics and Evaluation (IHME) website [http://ghdx.healthdata.org]. Pancreatic cancer has a poor prognosis and a short survival period for patients. The mortality can accurately reflect the contribution of smoking to the mortality burden of pancreatic cancer. Meanwhile, the DALYs can comprehensively consider both mortality and disease on health life expectancy, providing a more holistic quantification of the overall health loss caused by smoking. We extracted raw data from the GBD, including estimates of DALYs and deaths in absolute numbers, as well as age-standardized rates (ASRs) to conduct analysis (14). The data for this study were sourced from a publicly available database, requiring no ethical approval or informed consent. No ethical approval and informed consent were required because of the public availability of GBD and no identifiable information was included in the analyses.

### Definition of PC and smoking

GBD 2021 provided age-sex-location and yearly estimates of PC prevalence, which were derived from pooled analyses across only one domain: the prevalence of PC. The smoking population in this study refers to individuals who use any tobacco product including all smoked tobacco products—eg. cigarettes, pipes, cigars, shisha, bidis, kreteks, and other locally smoked tobacco products daily or occasionally, including those who have used any smoking product in the past. While risks from smokeless tobacco, electronic cigarettes (also known as e-cigarettes), vaping products, heated tobacco products, chewing tobacco, and second-hand smoke are outside the scope of this study. For current smokers, exposure levels were estimated using two continuous measures: the number of cigarettes smoked per day and the cumulative number of cigarettes smoked per year. For former smokers, exposure was assessed based on the number of years since cessation (15).

### Disease burden indicators

YLLs were the product of the number of deaths and standard life expectancy at each age of death, and YLDs were the product of the prevalence of each sequela and its corresponding disability weight. DALYs were calculated by summing YLDs and YLLs. The mortality numbers and the mortality rates are also included in the analysis. We also reported estimates generated as age-standardized results (e.g. ASDR and ASMR) to allow comparison of estimates made for populations with different age structures. Due to the potential heterogeneity in PC attributable to smoking, and burden resulting from variations in population age structures, we employed ASR and AAPC to quantify the trends in DALYs and mortality rates associated with PC. AAPC refers to the average annual percentage change across multiple periods, which is used to describe the overall trend over a longer period. It is calculated by fitting a regression line to the natural logarithm of the rates and provides a summary measure of a trend over time. We revealed the large differences and trends in smoking-attributable PC’s DALYs and deaths between sexes, ages, regions, countries/territories, and SDIs. 95% uncertainty intervals (UIs) were generated for all final estimates as the 25th and 95th percentile values of 500 draws. The AAPC, along with its 95% confidence intervals (CIs), was determined using a linear regression model to quantify changes over time.

### Disease burden analysis

For descriptive Analysis, the first step in a more in-depth analysis of the study, the study described the distribution of the disease burden at different geographical levels (including global, the 21 geographic super-regions, and 204 countries and territories) based on the burden of disease indicators described above and created a global map of disease burden distribution, highlighting the regions with a high or a clear upward trend of disease burden. Additionally, stratifying age into five-year intervals, pyramid charts were drawn to show the differences in DALY number, DALY rate, mortality, and mortality rate between males and females across different age groups in each year. Furthermore, the distribution of disease burden across different SDI regions is also described. Based on the differences in disease burden across different SDI regions, Spearman’s rank-order correlation analysis was used to measure the intensity and direction of the correlations between the ASDR, ASMR, their AAPCs, and the SDI, and a two-tailed p-value was calculated. A p-value less than 0.05 was regarded as significant (16).

### Join-point analysis

Join-pint regression model is first proposed by Kim in 2000 (17). Based on the temporal characteristics of the disease distribution, this model builds a piecewise regression and identifies significant changes in trends, referred to as joint points. Model results can be summarized using the metrics of annual percentage change (APC) and AAPC and their 95%CI to evaluate the trend of independent intervals of piecewise functions and assess the average trend over the entire study interval. Trends were described as increasing or decreasing when the AAPC or APC was statistically significant according to a two-sided p-value < 0.05, and as stable otherwise.

### Decomposition analysis

We employed the decomposition method invented by Das Gupta (18) to quantify the impact of age structure, population growth, and epidemiological changes on the DALYs and mortality number of PC attributable to smoking. Age structure refers to the distribution of different age groups within a population. Population growth refers to changes in the total number of people in a region or country. Epidemiological changes refer to shifts or trends in the distribution and determinants of health-related states in populations, including the dynamics of disease occurrence, prevalence, and mortality, which can be influenced by various factors, including but not limited to environmental factors, Behavioral Factors, Socioeconomic Status, Demographic Shifts, Genetic Factors, Healthcare Infrastructure and Policies (19). This methodological approach quantifies the impact of each factor on total change thereby informing targeted interventions and effective public health strategies to reduce the disease burden.

### Predictive analytics

To better forecast the future trends of DALYs and mortality rate, We utilized the Bayesian Age-Period-Cohort (BAPC) model, the probabilistic forecasts obtained by which are well calibrated and not too wide, demonstrating better predictive performance than other methods. The model, trained on historical data up to 2021, capturing the underlying patterns and dynamics of disease burden indicators over time, enables us to anticipate the evolving epidemiological landscape of PC.

### Statistical analysis

Variables were expressed in numbers, percentages, and ratios. Pearson correlation analyses were conducted to evaluate the associations between ASR and SDI in 2021, AAPC, and the mean SDI of 204 countries and territories. All statistical analyses and visualizations for this study were carried out using R 4.1.2 software. A significance level of P < 0.05 was used to determine statistical significance.

## Result

### Global burden of PC attributable to smoking from 1990 to 2021

Globally, the absolute burden of PC attributable to smoking has increased compared to 1990 (**Table 1**). The total DALYs rose from 1030311.87(95% UI,935957.37 - 1124871.5) in 1990 to 1789502.84(95% UI,1567220.76 - 2042056.59) in 2021. Similarly, the number of deaths increased from 38430.87(95% UI,34757.93 - 42214.02)in 1990 to 72170.03(95% UI,62852.94 - 82937.45) in 2021. However, after adjusting for time and age structure effects, the overall burden of PC attributable to smoking showed a certain degree of downward trend. The ASDR per 100,000 people decreased from 24.98(95% UI,22.66 -27.32) in 1990 to20.38(95% UI,17.84-23.25)in 2021 (AAPC,-0.65 [95% CI,-0.72 –0.58], P = 0) and the ASMR per 100,000 people decreased from 0.98(95% UI,0.89-1.08)in 1990 to 0.83(95% UI,0.73-0.96)in 2021(AAPC,-0.51[95% CI,-0.62 - -0.4], P = 0). Overall, on a global scale, despite noticeable increases in the total DALYs and the number of deaths compared to 30 years ago, after adjusting for the effects of time and age structure, downward trends have been observed both in ASDR and ASMR(**Table 1**).

**Table 1:** Global DALY and mortality of PC attributable to smoking and their AAPC by gender, SDI level, and region.

Characteristics	DALY 1990		DALY 2021		AAPC,% (95% CI)	P	Mortality 1990		Mortality 2021		AAPC,% (95% CI)	P
	No. (95% UI)	ASDR(/100,000) (95% UI	No. (95% UI)	ASDR (/100,000) (95% UI)			No. (95% UI)	ASMR(/100,000) (95% UI)	No. (95% UI)	ASMR(/100,000) (95% UI)		
Global	1030311.87 (935957.37 to to 1124871.5)	24.98 (22.66 to 27.32)	1789502.84 (1567220.76 to 2042056.59)	20.38 (17.84 to 23.25)	-0.65 (-0.72 to -0.58)	0	38430.87 (34757.93 to 42214.02)	0.98 (0.89 to 1.08)	72170.03 (62852.94 to 82937.45)	0.83 (0.73 to 0.96)	-0.51 (-0.62 to -0.40)	0
Gender												
Male	818685.07 (741892.63 to 899032.97)	41.74 (37.87 to 45.89)	1454884.28 (1261445.24 to 1677719.51)	34.73 (30.11 to 40.01)	-0.58 (-0.69 to -0.47)	0	29557.00 (26761.76 to 32600.83)	1.65 (1.48 to 1.82)	57260.69 (49810.36 to 66081.29)	1.42 (1.23 to 1.65)	-0.45 (-0.56 to -0.35)	0
Female	211626.79 (190301.46 to 233263.01)	9.89 (8.88 to 10.91)	334618.56 (291427.56 to 377986.66)	7.25 (6.32 to 8.18)	-1.01 (-1.11 to -0.90)	0	8873.87 (7870.88 to 9847.19)	0.43 (0.38 to 0.47)	14909.33 (12685.61 to 17190.41)	0.32 (0.27 to 0.37)	-0.94 (-1.04 to -0.84)	0
SDI rank												
High SDI	434178.24 (395058.27 to 473716.53)	40.38 (36.80 to 43.96)	595957.57 (522740.92 to 672270.78)	31.07 (27.49 to 34.78)	-0.86 (-0.98 to -0.74)	0	17658.08 (15909.77 to 19453.73)	1.60 (1.44 to 1.76)	26649.72 (22763.3 to 30843.83)	1.28 (1.11 to 1.47)	-0.73 (-0.85 to -0.60)	0
High-middle SDI	359802.44 (327229.38 to 395587.9)	34.64 (31.52 to 38.11)	630579.72 (538232.20 to 737072.94)	31.89 (27.26 to 37.23)	-0.24 (-0.35 to -0.13)	0	12599.27 (11433.85 to 13843.48)	1.25 (1.13 to 1.37)	24348.06 (20923.71 to 28483.37)	1.22 (1.05 to 1.42)	-0.06 (-0.19 to 0.07)	0.353
Middle SDI	185818.1 (160778.93 to 214639.25)	16.35 (14.18 to 18.78)	432976.32 (358675.29 to 520051.3)	15.27 (12.70 to 18.32)	-0.22 (-0.32 to -0.12)	0	6385.44 (5551.39 to 7324.14)	0.62 (0.54 to 0.71)	16365.69 (13662.76 to 19625)	0.60 (0.50 to 0.72)	-0.08 (-0.17 to 0.01)	0.076
Low-middle SDI	40536.66(32495.43 to 48720.16)	6.16(4.94 to 7.40)	110333.25 (97373.04 to 124389.03)	7.26 (6.41 to 8.18)	0.56 (0.47 to 0.64)	0	1431.32 (1146.2 to 1724)	0.24 (0.19 to 0.29)	4085.32 (3610.2 to 4605.53)	0.29 (0.25 to 0.32)	0.61 (0.50 to 0.73)	0
Low SDI	8485.68 (6283.29 to 10532.55)	3.49 (2.59 to 4.32)	17693.37 (14284.6 to 22096.27)	3.27 (2.63 to 4.06)	-0.2 (-0.28 to -0.12)	0	302.37 (224.4 to 374.51)	0.14 (0.1 to 0.17)	643.79 (517.82 to 797.96)	0.13 (0.11 to 0.16)	-0.10 (-0.21to 0.00)	0.059
GBD regions												
Andean Latin America	1862.3 (1532.91 to 2269)	8.87 (7.29 to 10.83)	5405.35 (4023.65 to 7082.59)	9.02 (6.70 to 11.85)	0.21 (-0.31 to 0.74)	0.426	71.34 (58.43 to 87.34)	0.36 (0.29 to 0.44)	218.16 (161.7 to 287.54)	0.37 (0.28 to 0.49)	0.30 (-0.27 to 0.87)	0.31
Australasia	6556.23 (5864.42 to 7314.74)	28.26 (25.34 to 31.56)	8876.98 (7537.36 to 10408.49)	18.29 (15.63 to 21.32)	-1.36 (-1.79 to -0.93)	0	264.67 (235.8 to 299.14)	1.12 (0.99 to 1.26)	385.25 (317.94 to 468.87)	0.73 (0.61 to 0.87)	-1.35 (-1.74 to -0.96)	0
Caribbean	4492.11 (4015.87 to 5015.79)	17.1 (15.28 to 19.11)	8629.13 (7272.39 to 10391.72)	15.94 (13.44 to 19.16)	-0.24 (-0.64 to 0.16)	0.244	180.94 (159.81 to 204.59)	0.70 (0.62 to 0.80)	341.19 (282.11 to 415.65)	0.63 (0.52 to 0.77)	-0.39 (-0.73 to -0.04)	0.029
Central Asia	6390.53 (5383.26 to 7705.88)	12.55 (10.58 to 15.12)	14922.03 (12859.69 to 17248.01)	16.31 (14.09 to 18.84)	0.90 (0.20 to 1.59)	0.011	207.45 (174.76 to 248.78)	0.42 (0.35 to 0.51)	509.36 (439.77 to 587.42)	0.60 (0.52 to 0.69)	1.14 (0.63 to 1.66)	0
Central Europe	67829.86 (62389.85 to 74103.77)	44.63 (41.00 to 48.68)	82013.18 (71556.7 to 92023.03)	40.85 (35.74 to 45.7)	-0.29 (-0.44 to -0.13)	0	2386.31 (2183.42 to 2608.22)	1.57 (1.44 to 1.72)	3211 (2782.84 to 3616.47)	1.50 (1.31 to 1.69)	-0.13 (-0.29 to 0.03)	0.109
Central Latin America	13422.59 (12142.57 to 14802.97)	15.48 (13.97 to 17.11)	25143.25 (21172.55 to 29255.2)	9.77 (8.22 to 11.35)	-1.52 (-1.88 to -1.16)	0	506.73 (455.07 to 561.57)	0.63 (0.56 to 0.70)	984.74 (830.09 to 1151.89)	0.39 (0.33 to 0.46)	-1.54 (-1.85 to -1.23)	0
Central Sub-Saharan Africa	1163.72 (878.33 to 1451.89)	4.55 (3.45 to 5.66)	2814.03 (1955.14 to 4005.12)	4.33 (3.01 to 6.16)	-0.14 (-0.25 to -0.04)	0.006	39.15 (29.65 to 48.92)	0.17 (0.13 to 0.21)	91.87 (64.01 to 130.85)	0.16 (0.11 to 0.22)	-0.18 (-0.28 to -0.08)	0.001
East Asia	252648.74 (205445.64 to 305786.27)	26.34 (21.53 to 31.88)	617563.85 (467039.28 to 787817.41)	27.46 (20.83 to 34.84)	0.12 (0.00 to 0.23)	0.044	8556.82 (7044.41 to 10345.24)	0.98 (0.81 to 1.18)	23911.43 (18358.81 to 30416.35)	1.08 (0.83 to 1.37)	0.30 (0.15 to 0.45)	0
Eastern Europe	101951.88 (91996.85 to 114168.73)	36.12 (32.61 to 40.37)	121954.38 (106677.46 to 137712.18)	36.84 (32.25 to 41.51)	0.23 (-0.19 to 0.66)	0.285	3330.38 (3005.77 to 3706.09)	1.17 (1.05 to 1.30)	4284.08 (3751.72 to 4856.59)	1.24 (1.09 to 1.41)	0.36 (-0.04 to 0.77)	0.077
Eastern Sub-Saharan Africa	3125.88 (2391.06 to 3851.65)	3.96 (3.05 to 4.87)	6757.61 (5119.76 to 9102.15)	3.71 (2.84 to 4.97)	-0.21 (-0.29 to -0.13)	0	113.86 (87.86 to 139.57)	0.16 (0.12 to 0.20)	242.22 (186.54 to 323.44)	0.15 (0.12 to 0.2)	-0.22 (-0.29 to -0.14)	0
High-income Asia Pacific	88407.67 (81374.69 to 95249.62)	42.54 (39.09 to 45.94)	110310.89 (96130.04 to 125424.14)	27.76 (24.48 to 31.06)	- 1.37 (-1.63 to -1.10)	0	3472.76 (3176.64 to 3779.74)	1.71 (1.57 to 1.87)	5447.34 (4597.42 to 6355.8)	1.18 (1.02 to 1.35)	-1.24 (-1.43 to -1.05)	0
High-income North America	136893.7 (123064.22 to 152181.37)	41.12 (37.10 to 45.56)	196657.35 (169599.35 to 227339.22)	31.34 (27.17 to 36.03)	to 0.89 (-1.08 to -0.71)	0	5655.00 (5033.68 to 6373.82)	1.62 (1.45 to 1.82)	8644.33 (7322.36 to 10306.75)	1.30 (1.11 to 1.54)	-0.73 (-0.88 to -0.57)	0
North Africa and Middle East	24404.24 (19650.89 to 30218.89)	13.15 (10.57 to 16.19)	80277.12 (67441.07 to 93215.42)	16.19 (13.57 to 18.9)	0.66 (0.57 to 0.76)	0	840.04 (673.65 to 1032.32)	0.50 (0.4 to 0.61)	2907.45 (2439.11 to 3407.65)	0.65 (0.54 to 0.76)	0.84 (0.75 to 0.94)	0
Oceania	257.93 (197.66 to 334.97)	7.67 (5.92 to 9.94)	703.04 (538.01 to 935.12)	8.03 (6.18 to 10.55)	0.17 (0.00 to 0.33)	0.047	8.56 (6.62 to 11.16)	0.29 (0.22 to 0.37)	23.09 (17.78 to 30.32)	0.30 (0.23 to 0.39)	0.14 (-0.02 to 0.30)	0.085
South Asia	28380.15 (21609.70 to 34869.59)	4.56 (3.45 to 5.63)	64073.19 (53827.37 to 74888.21)	4.16 (3.5 to 4.85)	-0.29 (-0.47 to -0.11)	0.002	1002.41 (757.57 to 1241.44)	0.18 (0.13 to 0.22)	2471.79 (2078.03 to 2889.31)	0.17 (0.14 to 0.2)	-0.08 (-0.27 to 0.12)	0.424
Southeast Asia	30559.87 (26091.41 to 35406.26)	11.01 (9.38 to 12.77)	96075.62 (80159.61 to 114510.53)	13.45 (11.27 to 16.06)	0.66 (0.58 to 0.75)	0	1067.37 (910.06 to 1240.05)	0.42 (0.36 to 0.49)	3463.91 (2919.3 to 4137.03)	0.52 (0.44 to 0.62)	0.69 (0.60 to 0.77)	0
Southern Latin America	18325.29 (16236.92 to 20633.29)	38.88 (34.45 to 43.76)	26129.15 (22977.27 to 29697.78)	31.09 (27.4 to 35.29)	-0.7 (-0.99 to -0.42)	0	668.68 (587.37 to 758.03)	1.42 (1.25 to 1.61)	1001.64 (871.75 to 1151.93)	1.16 (1.01 to 1.33)	-0.64 (-0.88 to -0.39)	0
Southern Sub-Saharan Africa	5206.45 (4337.73 to 6677.98)	17.83 (14.76 to 23.18)	10989.59 (9322.78 to 12750.14)	17.28 (14.67 to 20.11)	-0.14 (-0.65 to 0.38)	0.593	179.26 (147.7 to 235.13)	0.67 (0.55 to 0.88)	376.08 (320.32 to 437.84)	0.64 (0.54 to 0.74)	-0.17 (-0.64 to 0.31)	0.49
Tropical Latin America	25737.98 (23301.41 to 28159.48)	26.52 (23.94 to 29.16)	48804.15 (42173.31 to 55815.98)	18.51 (15.97 to 21.20)	-1.12 (-1.32 to -0.92)	0	941.28 (844.82 to 1042.2)	1.05 (0.94 to 1.18)	1929.37 (1637.77 to 2246.26)	0.75 (0.63 to 0.87)	-1.04 (-1.32 to -0.75)	0
Western Europe	211213.79 (190439.86 to 230437.02)	38.79 (35.10 to 42.2)	256629.57 (226671.47 to 290103.56)	31.65 (28.22 to 35.32)	-0.66 (-0.78 to -0.54)	0	8885.36 (7928.47 to 9813.6)	1.55 (1.38 to 1.70)	11560.35 (9964.08 to 13365.13)	1.29 (1.12 to 1.46)	-0.60 (-0.72 to -0.48)	0
Western Sub-Saharan Africa	1480.97 (1207.51 to 1754.72)	1.57 (1.29 to 1.86)	4773.39 (3811.19 to 5868.25)	2.17 (1.73 to 2.66)	1.06 (1.01 to 1.11)	0	52.49 (43.34 to 62.31)	0.06 (0.05 to 0.07)	165.36 (131.42 to 202.57)	0.08 (0.07 to 0.10)	1.08 (1.03 to 1.14)	0

PC, pancreatic cancer; DALYs, disability-adjusted life years; ASDR, age-standardized disability-adjusted life year; ASMR, age-standardized mortality rate; SDI, socio-demographic index; AAPC, average annual percentage changes.

### The regional burden of PC attributable to smoking from 1990 to 2021

**Figure 1:**
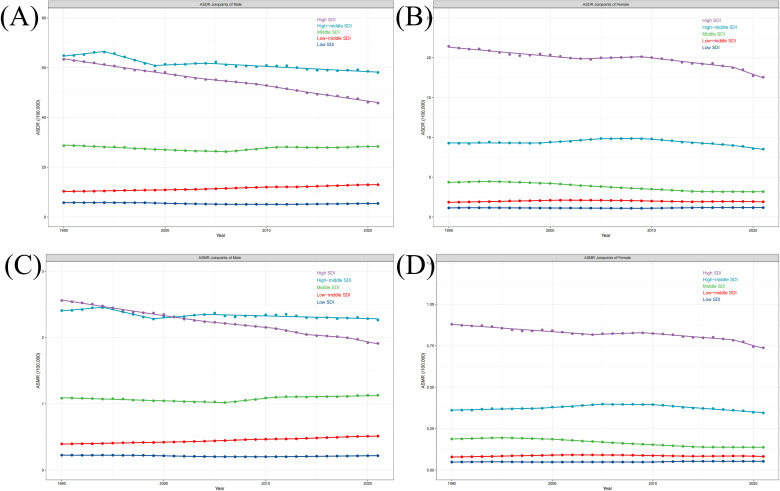
The burdens of PC attributable to smoking among 204 countries and territories in 2021. **(A)** Regional distribution of the ASDR of PC attributable to smoking in 2021. **(B)** Regional distribution of trends in ASDR from 1990 to 2021. **(C)** Regional distribution of the ASMR of PC attributable to smoking in 2021. **(D)** Regional distribution of trends in ASMR from 1990 to 2021. (PC, pancreatic cancer; ASDR, age-standardized disability-adjusted life year; ASMR, age-standardized mortality rate).

Regionally, the burden of PC attributable to smoking varied significantly across the 21 geographic super-regions(**Table 1**, **Figure 1**). East Asia and Oceania each recorded the highest and the lowest number of DALYs in both 1990 and 2021, respectively. However, the highest and the lowest records of the ASDR in both 1990 and 2021 are held by Central Europe and Western Sub-Saharan Africa, respectively, with Central a downward trend in Europe and a notable upward trend in Western Sub-Saharan Africa. Rising from 1.57 per 100,000 (95% UI,1.29 -1.86) in 1990 to 2.17 per 100,000(95% UI, 1.73-2.66)in 2021, with an AAPC of 1.06 ([95% CI, 1.01 - 1.11], p=0), Western Sub-Saharan Africa experienced the most significant increase in ASDR during this period. Conversely, decreasing from 15.48 per 100,000(95% UI, 13.97 - 17.11) in 1990 to 9.77 per 100,000(95% UI, 8.22 - 11.35)in 2021, with an AAPC of -1.52([95% CI,-1.88 - -1.16], p=0), Central Latin America experienced the largest declines in ASDR. Deaths and ASMR were generally higher across all regions, with East Asia recording the highest number of mortality and Central Europe recording the ASMR in 2021. It is worth mentioning that, similar to its ASDR, although Western Sub-Saharan Africa showed the lowest ASMR in both 1990 and 2021, it exhibited a significant upward trend in ASMR with an AAPC of 1.08 ([95% CI,1.03 - 1.14], p=0), second only to Central Asia, which had the highest increase with an AAPC of 1.14 ([95% CI, 0.63 - 1.66], p=0). Similar to ASDR, the largest decline in ASMR occurred in Central Latin America, decreasing from 0.63(95% UI, 0.56-0.7)in 1990 to 0.39(95% UI, 0.33-0.46), with an AAPC of -1.54 ([95% CI,-1.85 to - 1.23], p=0).

### National burden of PC attributable to smoking from 1990 to 2021

Nationally, the burden of PC attributable to smoking varied significantly across different countries and territories. In 2021, the ASDR and ASMR per 100,000 people ranged from 0.47(95% UI, 0.34 - 0.62)and 0.02(95% UI, 0.01 - 0.03)in Nigeria to 83.2(95% UI, 66.05 - 102.38) and 3.15(95% UI, 2.46 - 4) in Greenland. In the rankings for ASDR and ASMR in 2021, countries Greenland, Greece, Montenegro, and Armenia are all among the top 5(**Figures 1A, C**). Despite the highest ASDR or ASMR globally, China and the United States of America had the highest absolute numbers of DALYs and deaths, driven by their large populations. For a similar reason, the Russian Federation, Japan, Germany, and India are all ranked in the top six for both of the aforementioned indicators. From 1990 to 2021, the burden of PC attributable to smoking has shifted significantly across different countries (**Figures 1B, D**). San Marino and Ireland experienced the most substantial decreases in both ASDR (AAPCSan Marino = -2.49 [95% CI, -2.89 - -2.08], P < 0.001, AAPCIreland = -2.43 [95% CI, -3.05 - -1.81], P < 0.001) and ASMR (AAPCSan Marino = -2.61[95% CI, -2.82 - -2.4), P < 0.001, AAPCIreland = -2.43 [95% CI, -2.89 - -1.98, P < 0.001), while Turkmenistan and Cabo Verde experienced the most substantial increases in both ASDR (AAPCTurkmenistan = 8.03 [95% CI, 5.52 - 10.6], P < 0.001, AAPCCabo Verde = 7.33 [95% CI, 6.26 - 8.4], P < 0.001)) and ASMR (AAPCTurkmenistan = 8.17 [95% CI, 5.66 - 10.74), P < 0.001, AAPCCabo Verde = 7.46 [95% CI, 6.12 - 8.81], P < 0.001). In contrast, Fiji, Germany, and the United States Virgin Islands exhibited relatively stable ASDR and ASMR, with the 95%CI for their AAPCs clustering around zero, indicating minimal changes in the disease burden(**Supplementary Table S1**). Overall, the trends in ASDR closely mirrored the trends in ASMR, reflecting consistent patterns in the burden of smoking-related PC across nations.

### PC burden attributable to smoking by sex and age group

**Figure 2:**
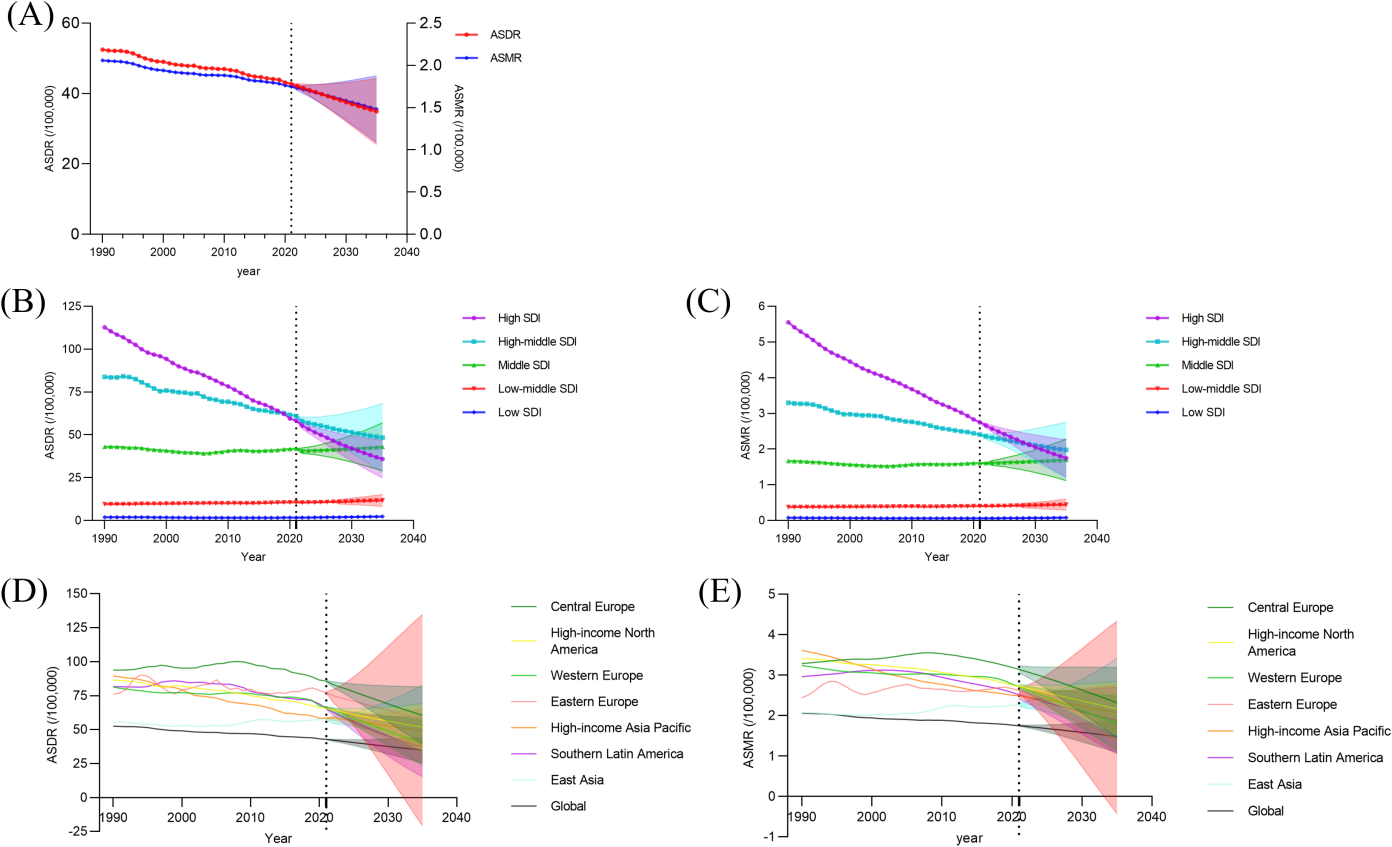
The burdens of PC attributable to smoking of different age groups from 1990 to 2021. The DALYs **(A)**, ASDR **(B)**, Death **(C)**, mortality rate **(D)** of different age groups from 1990 to 2021. (PC, pancreatic cancer; ASDR, age-standardized disability-adjusted life year; ASMR, age-standardized mortality rate).

In the sex subgroup analysis, the burden of PC attributable to smoking was significantly higher in males compared to females (**Figure 2**). In the age subgroup analysis in 2021, DALY rates rose with age, peaking at 70-74 years for both sexes, at 199.4/100,000 (95% UI, 170.18 - 234.02) for males and 47.5/100,000 (95% UI, 39.67 - 56.89)for females, and then declined. The trend in mortality rates was similar for both sexes, with a peak at 90-94 years 14.82/100,000 (95% UI, 11.41 - 18.81) for males and 4.81/100,000 (95% UI, 3.25 - 6.42) for females (**Supplementary Table S2**). From 1990 to 2021, although DALYs for PC attributable to smoking rose to some extent each year, the ASDR reduced significantly for both sexes, with a more pronounced decrease in females (AAPCASDR = -1.01 [95% CI, -1.11 to -0.9], P < 0.001). Likewise, the number of deaths rose significantly for both sexes, but the ASMR experienced a decline to a certain extent for both sexes, with a more significant decrease in females (AAPCASMR = -0.94 [95% CI, -1.04 - -0.84], P < 0.001) (**Figure 2**, **Table 1**).

### PC burden attributable to smoking by SDI

**Figure 3:**
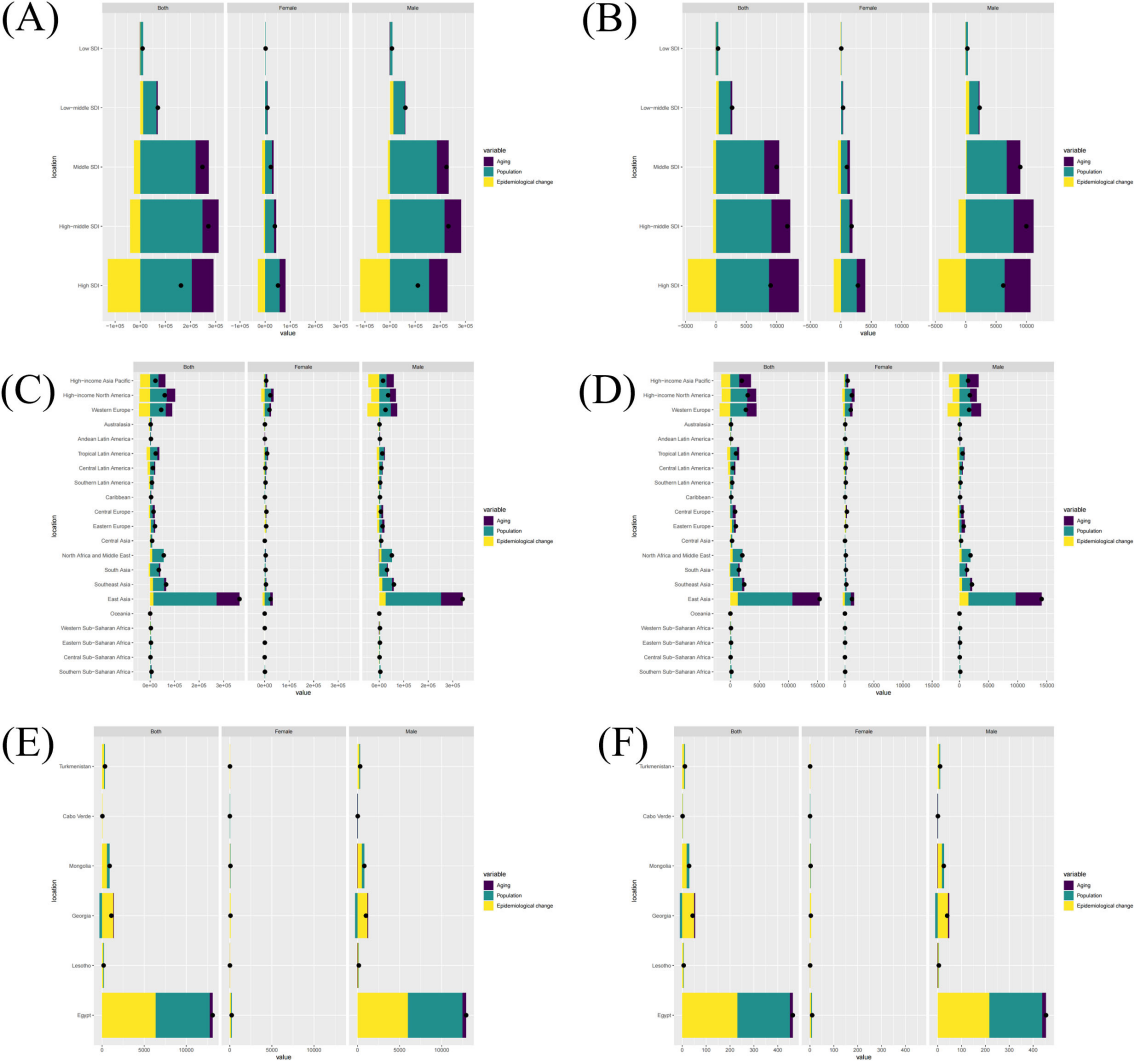
The correlation between SDI and ASR in 21 regions and 204 countries. The correlation between SDI and ASDR **(A)** or ASMR **(B)** in 21 regions. The correlation between SDI and ASDR **(C)** or ASMR **(D)** in 204 countries. The correlation between mean SDI and the AAPC of ASDR **(E)** or the AAPC of ASMR **(F)** in 204 countries. (ASDR, age-standardized disability-adjusted life year; ASMR, age-standardized mortality rate; AAPC, average annual percentage change).

According to the SDI classification in 2021, both ASDR and ASMR exhibited some degree of positive correlation with SDI levels across most regions, with the high SDI category displaying the highest ASDR and ASMR (**Supplementary Figure S1**). Although joint-point analysis indicated that this positive correlation persisted in most areas, further analysis revealed significant differences in the relationship between ASR and SDI across the 21 geographic regions and 204 countries (**Figures 3A–D**). At the 21 geographic regions level, contrary to the majority of other regions, the ASR exhibited a negative correlation with the SDI level in high-income Asia Pacific, High−income North America, Western Europe, and Australasia (**Figures 3A, B**). At the national level (**Figures 3C, D**), most countries fulfilled the condition of a positive correlation between ASRs and SDI levels, but there were still a few countries with significant deviations. From 1990 to 2021, changes in ASRs for PC attributable to smoking have been inconsistent across SDI subregions (**Table 1**). In most regions with different SDI levels, except in the Low-middle SDI region, both ASDR and ASMR showed a decreasing trend. Particularly, the decline in ASDR intensified with increasing SDI levels, with the most significant decrease in ASDR occurring in the High SDI region, reducing from 40.38/100,000 (95% UI, 36.80 - 43.96) in 1990 to 31.07/100,000 (95% UI, 27.49 - 34.78) in 2021, AAPC = -0.86 [95% CI, -0.98 - -0.74]. Similarly, the most significant decrease in ASMR also occurred in the High SDI region, from 1.6/100,000 (95% UI, 1.44 - 1.76) in 1990 to 1.28/100,000 (95% UI, 1.11 - 1.47) in 2021, with an AAPC = -0.73 [95% CI, -0.85 - -0.6]. Notably, the low SDI region also showed a certain degree of reduction in ASMR, more pronounced compared to other regions, with an AAPC of -0.1 [95% CI, -0.21 - 0] (**Table 1**). The above AAPC trends were also reflected at the national level(**Figures 3E, F**).

### Trends in ASDR and ASMR across time, sex, and SDI

**Figure 4:**
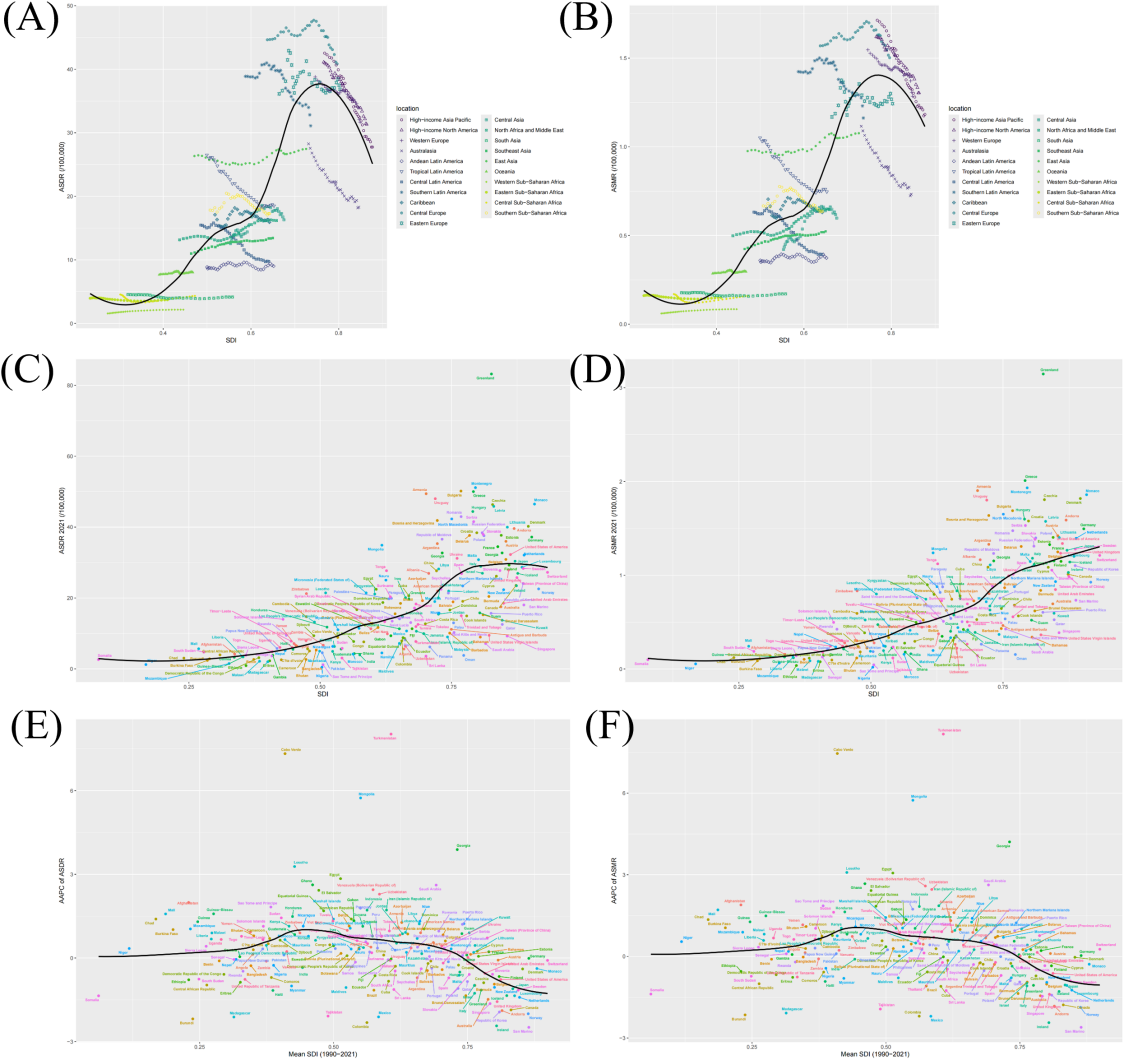
The join-point analysis of ASR change by sex and SDI, 1990 to 2021. The join-point analysis for PC attributable to smoking in ASDR by SDI of male **(A)**, female **(B)**. The join-point analysis for PC attributable to smoking in ASMR by SDI of male **(C)**, female **(D)**. (PC, pancreatic cancer; ASDR, age-standardized disability-adjusted life year; ASMR, age-standardized mortality rate; SDI, Socio-demographic Index).

Globally, the overall burden of PC attributable to smoking showed a certain degree of downward trend from 1990 to 2021 (**Table 1**). Based on different SDI categories, apart from a more pronounced downward trend in ASDR and ASMR shown in the High SDI region and a more pronounced upward trend in the Low-middle SDI region, the trends in other SDI regions are almost uniform, remaining stable or slightly decreasing(**Supplementary Figure S1**, **Supplementary Table S3**). Further analyzing the changing trends of disease burden by gender, we observed that in the High SDI region, the declines in ASDR and ASMR for males were more pronounced than for females (**Figure 4**). Maintaining a relatively stable level, compared to females, the ASDR and ASMR in High-middle and Middle SDI regions for males are relatively higher, which are higher than in Low-middle and Low SDI regions to a greater extent than females (**Figure 4**). The distinct levels of burden between genders are indicated.

### Decomposition analysis

**Figure 5:**
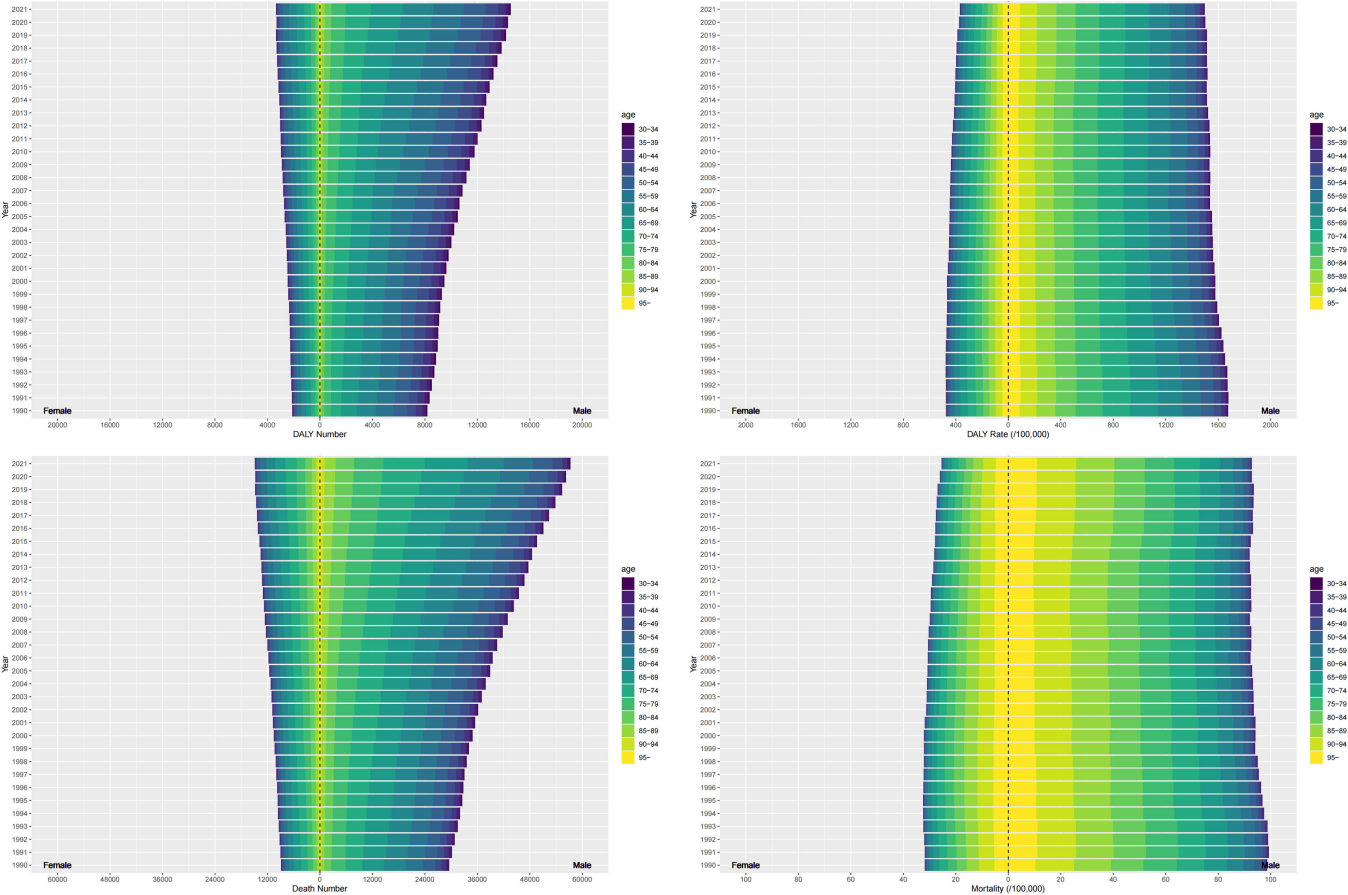
The decomposition analysis of PC burden attributable to smoking, 1990 to 2021. Changes in PC attributable to smoking DALYs **(A)** and mortality **(B)** according to population-level determinants of ageing, population growth, and epidemiological change from 1990 to 2021 at the global level by SDI quintile. Changes in PC attributable to smoking DALYs **(C)** and mortality **(D)** according to population-level determinants of ageing, population growth, and epidemiological change from 1990 to 2021 at the 21 regions level. Changes in PC attributable to smoking DALYs **(E)** and mortality **(F)** according to population-level determinants of ageing, population growth, and epidemiological change from 1990 to 2021 of the top six countries with the most DALYs and mortality increased. The black dot represents the overall value of change contributed by all 3 components. For each component, the magnitude of a positive value indicates a corresponding increase in PC attributable to smoking attributed to the component; the magnitude of a negative value indicates a corresponding decrease in PC attributable to smoking attributed to the related component. (PC, pancreatic cancer; DALYs, Disability-adjusted life-years).

Our decomposition analysis provided insights into the relative contributions of aging, population growth, and epidemiological changes to DALYs and mortality of PC attributable to smoking according to various regions, intending to clearly highlight the primary driving factor (**Figure 5**). According to five SDI regions, High-middle SDI and Middle SDI regions had obvious increases in DALYs and death numbers, primarily driven by population growth followed by aging (**Figures 5A, B**). In contrast, though facing population growth and aging of almost the same magnitude as the High-middle SDI and Middle SDI regions, the increase in DALYs and mortality numbers in High SDI regions is less, largely due to the significant positive impact of the epidemiological changes. According to the 21 GBD regions, in addition to the major distributions of population growth and aging, unfavorable epidemiological changes also contributed to the noticeable increase in the above indicators in East Asia. Conversely, epidemiological changes in High-income Asia Pacific, High-income North America, and Western Europe played a role in mitigating the increase in disease burden to some extent (**Figures 5C, D**). When it comes to the national level, we were primarily concerned with the top six nations with the largest increases in DALYs and death numbers, where the significant increases were mainly due to the substantial negative impact of epidemiological changes (**Figures 5E, F**). In terms of gender, there were no significant differences between males and females.

### Projection analysis

**Figure 6:**
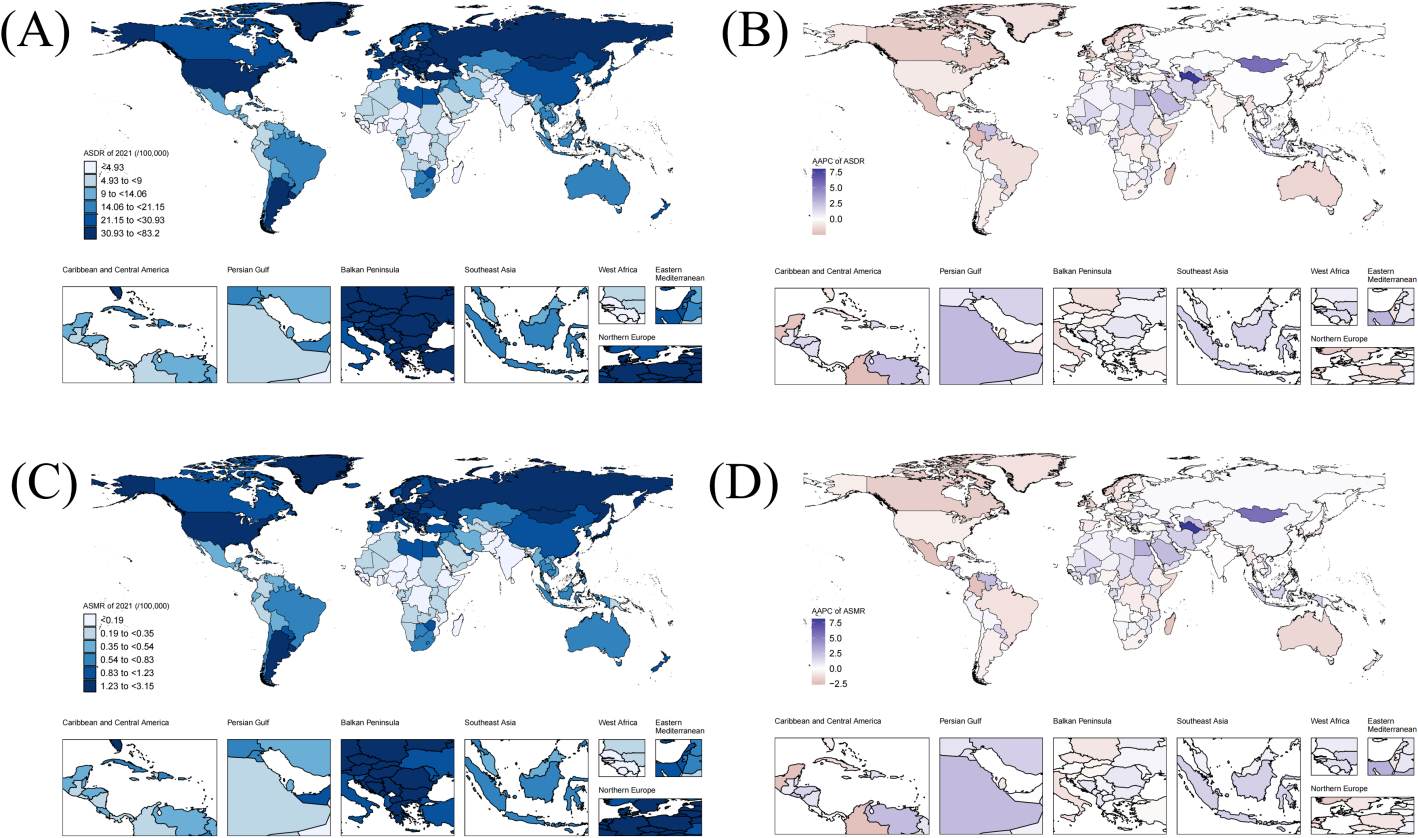
The predictive analysis of PC burden attributable to smoking,1990 to 2035. The global predictive analysis of PC attributable to smoking in ASDR and ASMR **(A)**. The predictive analysis of PC attributable to smoking of ASDR **(B)** and ASMR **(C)** in different SDI regions. The predictive analysis of PC attributable to smoking of ASDR **(D)** and ASMR **(E)** in the seven geographic super-regions with the highest disease burden in 2021.

To predict trends in the age-standardized DALY rate and mortality rate from 2020 to 2035, BAPC was performed. Globally, both ASDR and ASMR are projected to continue a similarly pronounced downward trend, reflecting the consistency between the two indicators (**Figure 6A**, **Supplementary Table S4**). Specifically for five regions, the trends of ASDR and ASMR are also similar. The decreasing trends of ASDR and ASMR are more pronounced in high and high-middle SDI regions, especially in high SDI regions, while there is a certain degree of increase in middle SDI areas, which needs to be brought to our attention, and remains relatively stable in other regions (**Figures 6B, C**, **Supplementary Table S5**). To better draw attention to the priority geographic super-regions and facilitate the implementation of effective measures at a more specific level, we have further predicted the trend of disease burden changes in subsequent years in the seven geographic super-regions with the highest disease burden in 2021. The results indicate that although the overall ASDR and ASMR levels in these 7 regions are significantly higher than the global average, it is encouraging to see that the majority of them are experiencing a downward trend over the coming years. However, it is concerning that the disease burden continues to rise in East Asia, with projections estimating the ASDR per 100,000 people at 60.70 and the ASMR per 100,000 people at 2.42 by 2035, making it the region with the highest disease burden (**Figures 6D, E**, **Supplementary Table S6**). It is worth noting that in some regions with sparse data or low predicted values, the lower limit of the 95% uncertainty interval is slightly below zero. This reflects the statistical uncertainty in the model estimates rather than implying negative values in reality.

## Discussion

As one of the leading causes of cancer-related mortality, PC remains a significant global health issue. Smoking continues to be a potentially modifiable lifestyle risk factor with the strongest correlation with PC. It is necessary and meaningful to conduct disease studies on the burden of disease of PC attributable to smoking. In our study, for the first time, we have detailed the global burden and evolving trends of PC attributable to smoking by leveraging the latest data from the GBD 2021. We not only reveal the differences in smoking-attributable PC DALYs and mortality between sexes, ages, regions, countries/territories, and SDIs but also further analyze the main factors affecting the burden of disease and predict the trend of the burden of disease in the coming period.

Smoking remains the most detrimental form of tobacco use. Previous basic research has identified that smoking plays a role in initiating the onset of PC and accelerating its progression, invasion, and metastasis (10, 11). In addition, this finding is corroborated by previous clinical studies showing a higher incidence rate and a lower survival rate after onset of the disease in smokers (7, 8). Since the implementation of effective tobacco control programs and policies, such as the WHO FCTC, the global prevalence of smoking has significantly declined (20, 21) despite the uneven distribution among regions, and smoke-free environments are increasingly prevalent worldwide (22).

Globally, despite the increasing absolute burden of the disease, after adjusting for time and age structure effects, the burden of the disease has shown a downward trend from 1990 to 2021, suggesting that epidemiological change had played a positive role. Around 2010, 5years following the implementation of the WHO FCTC, noticeable decreasing trends in both the global ASDR and ASMR were indicated by the Join-point regression model, which was likely related to the period of fastest decrease in the prevalence of smoking tobacco use across the largest number of countries. It is worth mentioning that the APCs of ASDR and ASMR in recent years are higher than in the past, suggesting that the progress made in tobacco control in recent years has played a remarkably effective role in the prevention and control of PC attributable to smoking (23). This has been confirmed in our decomposition analysis section. Encouragingly, our predictive analysis indicates a continued decline in ASDR and ASMR for the disease globally over the next few years. The realization of this prediction is contingent on the premise that continued implementation of strong tobacco control measures will be taken to create favorable epidemiological conditions that can neutralize the adverse effects of population aging and demographic changes on the burden of disease.

Despite the variations of the impact of smoking on the PC burden by regions and countries/territories, males bear a significantly greater burden of disease than females, and the trend of burden reduction is more pronounced in females than in males, which should be considered in cancer prevention and early screening. According to a systematic analysis of the prevalence of smoking tobacco use in 2019, this is highly likely due to the lower prevalence, shorter duration, and lower intensity of smoking in females than in males as well as the more significant reduction in global age-standardised prevalence of smoking tobacco use from 1990 among females (15). Moreover, the increased activation of androgen receptor expression in the PC cell lines and their surrounding tissues might be involved in the carcinogenesis and cancer development of PC and further lead to a higher burden of disease in males (24).

According to the prevalence of tobacco use from the GBD 2019, East Asia is the super-region with the largest number of tobacco smokers in 2019 due to its large population base (15). The highest number of DALYs and mortality in East Asia in our study is likely related to this situation. After adjusting for time and age structure effects, with its high age-standardized prevalence of smoking tobacco use (15), Central Europe has the highest ASDR and ASMR, consistent with patterns observed in other smoking-related diseases. With the large decreases in age-standardized prevalence of smoking tobacco use in Colombia and Costa Rica, the countries have continuously introduced anti-smoking laws that have severe penalties and high costs associated with consumption, the largest declines in ASDR and ASMR were observed in Central Latin America, showing the potential of the effective tools to operate to greatly reduce the prevalence of smoking tobacco use and save millions of lives over the coming decades. With almost the lowest tobacco consumption per person, the ASDR and ASMR of smoking tobacco in some economically underdeveloped regions, such as sub-Saharan Africa, have always maintained the lowest levels, but with the largest relative increases in the number of smokers during the period (15). The manifestation that Western Sub-Saharan Africa is experiencing almost the most significant increase in ASDR and ASMR warnings us that it is high time that strong tobacco control policies should be implemented and enforced to slow down the rate of increase in the burden of disease in this area with the economic recovery and growth.

With almost the lowest and the highest age-standardized prevalence of current use of smoking tobacco, Nigeria and Greenland have the corresponding levels of ASDR and ASMR. With 341 million (30%) of 1·14 billion tobacco smokers globally, China is the country with the largest number of tobacco smokers in 2019 likely because the reduction in prevalence has not kept pace with population growth. Sufficient decreases in the prevalence of smoking are needed to offset the large population base and the demographic force of population growth, further reducing the absolute burden of disease. Respectively approving the WHO FCTC in 2004 and 2005, becoming two of the early parties to the “convention”, and have implemented a series of policies to reduce tobacco consumption and related health risks, such as smoke-free environments, tobacco tax policies, and restrictions on tobacco advertising and promotion, San Marino and Ireland experienced the most substantial decreases in both ASDR and ASMR. Reiterating, proactive tobacco control policies play a significant role in reducing the burden of this disease.

Focus on the trends of the past 5-10 years, the trends in PC attributable to smoking have varied significantly across SDI regions, characterized by declining ASDR and ASMR in high SDI regions, while observing a mild increase or relative stability in low and low-middle SDI regions and a gradual increase in medium SDI regions. This divergence can be attributed to several factors: Countries with high SDI typically enforce stringent tobacco regulations (25), promote physical activity, optimize dietary habits, and possess advanced healthcare systems with robust screening capabilities, and this is also one of the reasons why the burden of disease in high-income regions such as Asia-Pacific and North America is inversely proportional to the level of SDI. Conversely, In low and low-middle SDI regions, the current level of tobacco control policy implementation is far from sufficient. Most of the countries that have yet to adopt a single MPOWER measure at the highest level of achievement belong to low and low-middle SDI regions. Of those 20 countries where cigarettes have become more affordable, 17 belong to low and low-middle SDI regions (26). The affordability of cigarettes and the lack of necessary restrictive measures have led to a high smoking prevalence in the regions, and coupled with its relatively backward medical level, the burden of disease has gradually increased. The higher burden of disease in medium SDI regions may be linked to improving economic conditions, which have led to increased smoking prevalence and the gradual enhancement of physical examination. The notable regional disparities in the burden of disease across SDI levels underscore socio-spatial inequities in the prevention and management of PC. Future efforts should focus on not only taking effective tobacco control measures such as raising taxes but also bolstering healthcare capacity in low and middle-income regions, alongside interregional collaborations aimed at promoting smoking cessation.

The main drivers behind changes in the burden of disease are threefold: age structure, population growth, and epidemiological changes. Each factor affects the burden of disease differently across regions and periods. The preliminary conclusion that a higher incidence rate is among the elderly enlightens us to observe the weight of each factor and highlight the primary driving factors. Our study indicated that the increases in DALYs and death numbers among most regions and countries were primarily driven by population growth followed by aging, and the variations in the growth rates of burdens of disease across different regions were influenced by the distinct roles that epidemiological changes had played. The epidemiological changes in High SDI areas have shown a positive impact in mitigating the increase in the absolute burden of disease, while the nations experiencing the most rapid burden growth have shown negative effects. Within the framework of the decomposition analysis model, “epidemiological change” refers to shifts in disease incidence, prevalence, and mortality rates, influenced by various factors such as pathogen variations (e.g. the emergence of highly virulent strains), the effectiveness of public health interventions (e.g., vaccination and antibiotic use), lifestyle changes (e.g. diet and exercise habits), environmental factors (e.g. climate change), and socioeconomic changes (e.g. access to and quality of healthcare resources). Epidemiological changes affect the spread and severity of diseases, thereby altering the burden of disease. Smoking as a potentially modifiable lifestyle risk factor with the strongest correlation with PC is an important component of epidemiological changes. Thus, under the general trend of population growth and aging, taking active measures to change epidemiological factors, such as restricting tobacco use, is critical to slow down the growth of the absolute burden of disease.

It is projected that over the next 15 years, most regions will continue their current trends in burden of disease changes. However, it is a cause for concern that East Asia, which is currently among the top 7 countries with the highest burden of disease and facing severe population growth and aging issues, will continue to show a significant upward trend. If effective intervention measures are not taken in time, it is anticipated that by 2035, this region will become the area with the highest burden of disease. Therefore, strengthening disease prevention efforts by restricting the use of tobacco and increasing investment in healthcare resources for PC attributable to smoking are imminent.

What needs to be clarified is that because the original purpose of DALYs was to emphasize the greater impact of death or disability in younger age groups on social productivity, it is inevitable that the burden of disease in younger individuals may be overinflated compared to it in older individuals, based on the calculation formula for DALYs mentioned earlier. Therefore, when describing the burden of disease, we introduce the ASDR indicator to adjust the time and age structure effects. When assessing the relationship between the burden of disease and age, similar to some top-tier articles that also stratify age into five-year intervals, we simply presented DALYs by age group, without comparing DALYs between groups with large age differences because of a certain degree of unfairness. Presenting DALYs by age group has its corresponding value. On the one hand, under the same number of disabilities, the DALYs calculated for younger populations should be higher than for older groups. However, our study data indicated that the DALYs in younger groups were significantly lower than in older groups, which further suggested that the burden of PC attributable to smoking in young people was significantly lower. On the other hand, the DALYs between two age groups with close ages are reasonably comparable. According to our data, the inter-group DALYs differences among the 45-49, 50-54, and 55-59 age groups were more pronounced than the differences amongother adjacent age groups, reflecting a clear trend of the increasing burden of disease with age in the middle-aged population. This is not only related to the age characteristics of the peak incidence of PC itself but also the cumulative increase in tobacco use over the years, according to current research which has shown that the risk of PC in smokers significantly increases with the amount of daily smoking, the number of years smoked, and the cumulative number of packs smoked (27).

Several limitations should be noted. Firstly, GBD data, including its complex modeling and data input, significant methodological changes with each revision, and the potential for assumptions and knowledge gaps to influence estimates. Additionally, the GBD database only controls for certain covariates, meaning that unadjusted covariates (such as dietary habits, lifestyle, medication use, metabolic and physiological changes, etc.) may influence the study results (28). Secondly, based on the insidious onset inherent characteristics of PC, there may be misdiagnosed and underreporting of cases in some regions and countries/territories with underdeveloped healthcare systems, especially in low-income areas, leading to a lack of high-quality and detailed data, potentially leading to an underestimation of the burden of disease. Thirdly, the exposure to tobacco may be underestimated to some extent. On the one hand, the data were sourced from public databases, and information regarding smoking habits relied on self-reporting, potentially resulting in an underestimation of smoking prevalence, particularly among females and younger individuals. On the other hand, the databases assessed only traditional smoking tobacco products, excluding emerging tobacco products like e-cigarettes, whose health implications are increasingly recognized (29). Consequently, reported cases of PC attributed to smoking in public health records may be lower than the actual prevalence. Prospective studies utilizing real-world data are warranted to assess the risk of PC among smokers. Such studies would provide more accurate assessments of the burden of PC attributable to smoking and inform targeted interventions to mitigate its public health impact.

## Conclusions

We analyzed the global burden of PC attributable to smoking and its variations across different regions, countries/territories, sexes, and age groups. Despite the decline in global smoking prevalence, PC attributable to smoking remains a significant issue, especially in high and high-middle-income regions and among males. Additionally, the progressive increase burden of disease in low-middle-income regions also deserves our attention. In the context of the general trend of population growth and aging, epidemiological factors have played an important role in influencing the trends in the burden of disease. Therefore, targeted tobacco control measures are essential to mitigate this persistent health burden, focusing on regions experiencing rising smoking prevalence and associated disease impacts.

## Data Availability

The datasets presented in this study can be found in online repositories. The names of the repository/repositories and accession number(s) can be found below: The dataset supporting the conclusions of this article is available in the Global Health Data Exchange repository. (https://vizhub.healthdata.org).
