# Supplementary material for: Global, regional, and national burdens of pancreatic cancer attributable to smoking from 1990 to 2021 and the projections to 2035:a systematic analysis from the global burden of disease study 2021
Source: Front Oncol. 2025 May 30;15:1547029. doi: 10.3389/fonc.2025.1547029 (PMC12163048; doi:10.3389/fonc.2025.1547029)
Supplement: Supplementary file 1 [file DataSheet1.docx]

**Supplementary Figure 1** The join-point analysis of ASR change by SDI from 1990 to 2021

**
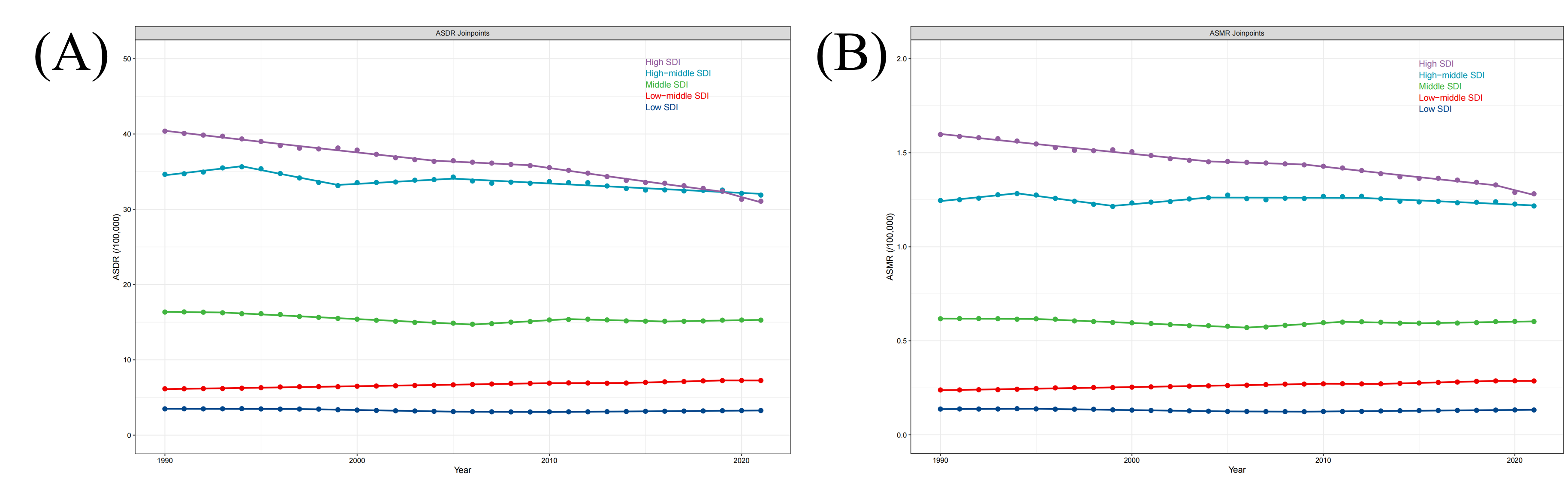
**

The join-point analysis of ASDR(A) and ASMR(B) change by SDI from 1990 to 2021.

**Note:** PC, pancreatic cancer; ASDR, age-standardized disability-adjusted life year; ASMR, age-standardized mortality rate; SDI, Socio-demographic Index.

**Supplementary Table 1** DALY and mortality of PC attributable to smoking and their AAPCs in 204 countries and territories

| **characteristics** | **DALY 1990** | | **DALY 2021** | | **AAPC,% (95% CI)** | **P** | **Mortality 1990** | | **Mortality 2021** | | **AAPC,% (95% CI)** | **P** |
| --- | --- | --- | --- | --- | --- | --- | --- | --- | --- | --- | --- | --- |
|  | **No (95% UI)** | **ASDR (/100,000) (95% UI)** | **No (95% UI)** | **ASDR (/100,000) (95% UI)** |  |  | **No. (95% UI)** | **ASMR (/100,000) (95% UI)** | **No. (95% UI)** | **ASMR (/100,000) (95% UI)** |  |  |
| Afghanistan | 168.95 (82.22 to 331.70) | 2.27 (1.13 to 4.42) | 505.55 (274.27 to 884.16) | 4.16 (2.37 to 7.25) | 1.99 (1.91 to 2.07) | 0 | 6.14 (3.09 to 11.84) | 0.09 (0.04 to 0.17) | 15.84 (9.02 to 27.43) | 0.16 (0.09 to 0.27) | 1.89 (1.81 to 1.97) | 0 |
| Albania | 438.76 (342.32 to 570.83) | 20.50 (16.03 to 26.45) | 1165.44 (799.61 to 1658.86) | 26.94 (18.52 to 38.22) | 0.83 (0.26 to 1.39) | 0.004 | 17.10 (13.43 to 21.94) | 0.87 (0.68 to 1.10) | 51.72 (35.80 to 72.90) | 1.17 (0.81 to 1.64) | 0.91 (0.42 to 1.4) | 0 |
| Algeria | 607.09 (473.07 to 765.81) | 4.96 (3.86 to 6.31) | 2022.42 (1460.62 to 2693.37) | 5.61 (4.02 to 7.51) | 0.39 (0.25 to 0.53) | 0 | 24.30 (18.71 to 31.08) | 0.23 (0.18 to 0.29) | 83.49 (59.30 to 110.98) | 0.26 (0.19 to 0.35) | 0.45 (0.26 to 0.63) | 0 |
| American Samoa | 4.29 (3.37 to 5.35) | 16.73 (13.23 to 20.78) | 11.75 (9.22 to 15.07) | 21.99 (17.31 to 27.96) | 0.9 (0.65 to 1.15) | 0 | 0.14 (0.11 to 0.18) | 0.62 (0.48 to 0.77) | 0.41 (0.32 to 0.51) | 0.80 (0.63 to 1.00) | 0.88 (0.62 to 1.13) | 0 |
| Andorra | 40.43 (27.41 to 57.14) | 68.30 (46.44 to 96.70) | 60.20 (38.00 to 85.30) | 39.60 (25.07 to 56.06) | -1.89 (-2.3 to -1.47) | 0 | 1.56 (1.06 to 2.20) | 2.69 (1.85 to 3.79) | 2.46 (1.58 to 3.44) | 1.59 (1.02 to 2.23) | -1.83 (-2.17 to -1.48) | 0 |
| Angola | 290.78 (203.43 to 407.03) | 6.43 (4.54 to 8.96) | 931.61 (596.93 to 1329.00) | 6.61 (4.31 to 9.43) | 0.13 (-0.14 to 0.41) | 0.343 | 9.50 (6.70 to 13.26) | 0.24 (0.17 to 0.33) | 30.19 (19.66 to 43.09) | 0.24 (0.16 to 0.35) | 0.14 (-0.1 to 0.37) | 0.249 |
| Antigua and Barbuda | 4.71 (3.83 to 5.60) | 9.43 (7.69 to 11.10) | 12.60 (10.53 to 14.80) | 11.17 (9.35 to 13.12) | 0.55 (-0.71 to 1.83) | 0.392 | 0.20 (0.16 to 0.24) | 0.37 (0.30 to 0.45) | 0.50 (0.41 to 0.60) | 0.46 (0.38 to 0.56) | 0.68 (-0.67 to 2.05) | 0.327 |
| Argentina | 14605.68 (12901.22 to 16616.46) | 44.42 (39.20 to 50.45) | 19005.61 (16492.87 to 21773.35) | 35.33 (30.72 to 40.37) | -0.71 (-1.05 to -0.37) | 0 | 532.67 (465.12 to 610.63) | 1.62 (1.42 to 1.86) | 733.95 (630.02 to 850.84) | 1.33 (1.14 to 1.54) | -0.6 (-0.93 to -0.27) | 0 |
| Armenia | 1095.34 (900.93 to 1340.78) | 36.49 (30.26 to 44.32) | 2137.37 (1737.04 to 2564.06) | 49.42 (40.23 to 59.25) | 1.09 (0.55 to 1.63) | 0 | 37.22 (31.03 to 45.46) | 1.31 (1.10 to 1.60) | 83.82 (68.28 to 100.76) | 1.90 (1.55 to 2.29) | 1.31 (0.79 to 1.85) | 0 |
| Australia | 5381.54 (4820.16 to 6011.96) | 27.84 (24.95 to 31.10) | 7091.29 (5992.56 to 8357.10) | 17.52 (14.88 to 20.68) | -1.48 (-1.89 to -1.08) | 0 | 1.09 (0.97 to 1.24) | 1.28 (1.14 to 1.45) | 0.69 (0.57 to 0.84) | 1.19 (0.97 to 1.45) | -1.45 (-1.87 to -1.02) | 0 |
| Austria | 3930.70 (3502.02 to 4373.80) | 36.77 (32.89 to 40.79) | 5757.09 (5027.92 to 6502.62) | 35.99 (31.63 to 40.63) | -0.08 (-0.47 to 0.31) | 0.693 | 157.17 (136.86 to 177.46) | 1.38 (1.22 to 1.55) | 245.43 (211.61 to 280.43) | 1.42 (1.23 to 1.61) | 0.11 (-0.2 to 0.41) | 0.492 |
| Azerbaijan | 715.88 (518.54 to 1015.30) | 12.96 (9.37 to 18.45) | 2236.85 (1482.38 to 3280.84) | 19.24 (12.92 to 27.89) | 1.27 (0.97 to 1.57) | 0 | 23.28 (16.95 to 33.02) | 0.44 (0.32 to 0.63) | 76.90 (52.10 to 110.47) | 0.73 (0.50 to 1.01) | 1.61 (1.32 to 1.9) | 0 |
| Bahamas | 14.61 (11.65 to 17.86) | 8.83 (7.05 to 10.88) | 39.68 (29.36 to 50.58) | 8.97 (6.66 to 11.46) | 0.13 (-0.48 to 0.75) | 0.678 | 0.50 (0.40 to 0.62) | 0.32 (0.26 to 0.40) | 1.41 (1.05 to 1.80) | 0.34 (0.25 to 0.44) | 0.25 (-0.31 to 0.81) | 0.379 |
| Bahrain | 41.00 (33.19 to 49.93) | 21.93 (17.41 to 26.78) | 192.44 (137.34 to 283.73) | 18.91 (13.88 to 26.94) | -0.45 (-0.74 to -0.17) | 0.002 | 1.47 (1.17 to 1.79) | 0.95 (0.76 to 1.16) | 6.77 (4.88 to 9.89) | 0.87 (0.64 to 1.20) | -0.25 (-0.67 to 0.18) | 0.26 |
| Bangladesh | 2906.35 (1610.66 to 4086.33) | 6.00 (3.34 to 8.45) | 7562.58 (5006.83 to 11370.21) | 5.33 (3.53 to 7.92) | -0.28 (-0.53 to -0.03) | 0.031 | 107.21 (60.02 to 150.18) | 0.24 (0.13 to 0.33) | 301.92 (201.35 to 443.49) | 0.22 (0.15 to 0.33) | -0.02 (-0.31 to 0.27) | 0.877 |
| Barbados | 32.84 (27.01 to 38.95) | 12.19 (10.13 to 14.37) | 50.66 (36.59 to 67.04) | 10.10 (7.32 to 13.39) | -0.42 (-0.86 to 0.02) | 0.061 | 1.45 (1.18 to 1.74) | 0.49 (0.40 to 0.58) | 2.24 (1.61 to 2.98) | 0.43 (0.31 to 0.57) | -0.29 (-0.9 to 0.33) | 0.354 |
| Belarus | 3831.76 (3377.95 to 4258.46) | 29.28 (26.11 to 32.58) | 5829.65 (4600.83 to 7284.03) | 37.65 (29.77 to 47.07) | 0.83 (0.42 to 1.23) | 0 | 136.12 (120.54 to 152.51) | 1.04 (0.92 to 1.16) | 209.11 (165.33 to 258.59) | 1.31 (1.04 to 1.62) | 0.69 (0.16 to 1.23) | 0.01 |
| Belgium | 5922.12 (5256.19 to 6614.40) | 40.40 (36.18 to 45.09) | 6313.95 (5499.87 to 7256.61) | 30.88 (27.09 to 35.18) | -0.94 (-1.4 to -0.47) | 0 | 254.95 (224.75 to 287.62) | 1.66 (1.46 to 1.86) | 281.36 (241.71 to 330.95) | 1.25 (1.08 to 1.45) | -1.02 (-1.72 to -0.31) | 0.005 |
| Belize | 7.76 (6.20 to 10.52) | 8.34 (6.65 to 11.27) | 37.83 (30.78 to 45.47) | 11.68 (9.54 to 14.05) | 1.14 (0.57 to 1.72) | 0 | 0.29 (0.23 to 0.40) | 0.32 (0.25 to 0.44) | 1.36 (1.11 to 1.63) | 0.45 (0.37 to 0.55) | 1.19 (0.58 to 1.8) | 0 |
| Benin | 56.06 (42.75 to 71.23) | 2.75 (2.11 to 3.49) | 147.33 (104.57 to 201.54) | 2.66 (1.91 to 3.61) | -0.1 (-0.27 to 0.06) | 0.223 | 0.11 (0.08 to 0.14) | 0.04 (0.03 to 0.06) | 0.10 (0.08 to 0.14) | 0.04 (0.03 to 0.05) | -0.1 (-0.29 to 0.08) | 0.258 |
| Bermuda | 16.74 (12.06 to 20.92) | 26.29 (18.89 to 32.93) | 25.76 (19.98 to 32.88) | 20.43 (15.82 to 25.91) | -0.98 (-1.56 to -0.4) | 0.001 | 0.66 (0.47 to 0.84) | 1.07 (0.76 to 1.37) | 1.14 (0.89 to 1.50) | 0.84 (0.65 to 1.08) | -0.95 (-1.54 to -0.35) | 0.002 |
| Bhutan | 5.47 (2.39 to 9.25) | 2.09 (0.92 to 3.50) | 16.29 (9.09 to 24.76) | 2.67 (1.50 to 4.08) | 0.79 (0.67 to 0.92) | 0 | 0.19 (0.08 to 0.32) | 0.08 (0.04 to 0.14) | 0.69 (0.40 to 1.05) | 0.12 (0.07 to 0.18) | 1.1 (0.97 to 1.23) | 0 |
| Bolivia (Plurinational State of) | 411.26 (280.89 to 550.02) | 12.24 (8.34 to 16.30) | 1222.23 (796.81 to 1766.73) | 12.80 (8.36 to 18.50) | 0.16 (0.03 to 0.3) | 0.015 | 15.09 (10.21 to 20.09) | 0.48 (0.32 to 0.63) | 47.33 (30.67 to 68.17) | 0.52 (0.34 to 0.75) | 0.29 (0.14 to 0.44) | 0 |
| Bosnia and Herzegovina | 1496.51 (1250.77 to 1778.10) | 33.29 (27.78 to 39.61) | 2512.42 (1929.49 to 3206.94) | 41.87 (32.23 to 53.81) | 0.86 (0.63 to 1.09) | 0 | 52.37 (43.65 to 62.24) | 1.25 (1.04 to 1.48) | 102.64 (78.88 to 129.01) | 1.64 (1.26 to 2.07) | 0.99 (0.78 to 1.2) | 0 |
| Botswana | 84.91 (61.54 to 118.10) | 13.96 (10.22 to 19.34) | 250.70 (164.77 to 370.58) | 15.55 (10.57 to 22.17) | 0.35 (0.1 to 0.6) | 0.005 | 3.01 (2.21 to 4.12) | 0.54 (0.40 to 0.74) | 8.95 (6.13 to 12.68) | 0.63 (0.44 to 0.85) | 0.46 (0.22 to 0.7) | 0 |
| Brazil | 25470.69 (23050.77 to 27862.60) | 26.87 (24.26 to 29.56) | 47630.63 (41215.00 to 54386.28) | 18.48 (15.96 to 21.14) | -1.15 (-1.29 to -1.02) | 0 | 1.06 (0.95 to 1.19) | 0.63 (0.56 to 0.69) | 0.74 (0.63 to 0.87) | 0.85 (0.72 to 1.00) | -1.09 (-1.37 to -0.8) | 0 |
| Brunei Darussalam | 24.94 (19.41 to 31.16) | 22.98 (17.85 to 28.71) | 60.55 (47.12 to 75.59) | 15.26 (11.89 to 18.98) | -1.35 (-1.6 to -1.1) | 0 | 0.91 (0.71 to 1.13) | 0.93 (0.72 to 1.17) | 2.13 (1.67 to 2.66) | 0.62 (0.48 to 0.78) | -1.36 (-1.61 to -1.11) | 0 |
| Bulgaria | 5143.62 (4459.55 to 5915.12) | 41.91 (36.29 to 48.01) | 6036.36 (4792.70 to 7380.04) | 50.17 (40.18 to 61.24) | 0.61 (-0.04 to 1.27) | 0.066 | 171.01 (148.33 to 198.09) | 1.37 (1.19 to 1.58) | 218.95 (173.67 to 268.49) | 1.69 (1.34 to 2.06) | 0.74 (0.1 to 1.38) | 0.023 |
| Burkina Faso | 83.80 (58.69 to 116.89) | 1.77 (1.24 to 2.46) | 249.91 (168.91 to 344.36) | 2.40 (1.64 to 3.30) | 1.02 (0.91 to 1.12) | 0 | 2.84 (2.00 to 3.92) | 0.06 (0.05 to 0.09) | 8.36 (5.75 to 11.45) | 0.09 (0.06 to 0.12) | 1.05 (0.88 to 1.22) | 0 |
| Burundi | 138.52 (99.85 to 182.12) | 5.66 (4.11 to 7.38) | 156.13 (98.15 to 255.08) | 2.84 (1.82 to 4.53) | -2.18 (-2.27 to -2.1) | 0 | 4.95 (3.63 to 6.41) | 0.21 (0.16 to 0.27) | 5.32 (3.41 to 8.45) | 0.11 (0.07 to 0.17) | -2.15 (-2.25 to -2.05) | 0 |
| C?te d'Ivoire | 109.64 (79.52 to 146.92) | 2.33 (1.70 to 3.08) | 340.24 (224.20 to 509.66) | 2.57 (1.73 to 3.76) | 0.3 (0.14 to 0.46) | 0 | 3.54 (2.59 to 4.70) | 0.09 (0.06 to 0.11) | 11.09 (7.45 to 16.22) | 0.10 (0.07 to 0.14) | 0.29 (0.14 to 0.44) | 0 |
| Cabo Verde | 2.28 (1.79 to 2.81) | 1.06 (0.84 to 1.31) | 42.66 (29.81 to 58.03) | 8.90 (6.07 to 12.13) | 7.33 (6.26 to 8.4) | 0 | 0.09 (0.07 to 0.11) | 0.04 (0.03 to 0.05) | 1.52 (1.02 to 2.09) | 0.34 (0.22 to 0.46) | 7.46 (6.12 to 8.81) | 0 |
| Cambodia | 616.52 (421.03 to 887.47) | 12.67 (8.74 to 18.11) | 1977.30 (1400.23 to 2646.10) | 14.74 (10.58 to 19.38) | 0.51 (0.44 to 0.58) | 0 | 21.71 (15.03 to 30.88) | 0.49 (0.34 to 0.69) | 72.16 (51.81 to 94.16) | 0.59 (0.43 to 0.76) | 0.63 (0.56 to 0.69) | 0 |
| Cameroon | 206.82 (152.52 to 278.10) | 4.06 (3.03 to 5.42) | 750.87 (507.58 to 1105.73) | 5.05 (3.43 to 7.51) | 0.7 (0.62 to 0.78) | 0 | 6.76 (5.08 to 9.03) | 0.15 (0.11 to 0.19) | 24.17 (16.37 to 35.91) | 0.18 (0.12 to 0.27) | 0.74 (0.66 to 0.82) | 0 |
| Canada | 13372.35 (11986.46 to 14959.33) | 41.74 (37.43 to 46.69) | 16013.32 (13477.47 to 18600.65) | 23.57 (20.04 to 27.21) | -1.86 (-2.24 to -1.48) | 0 | 557.09 (496.00 to 627.67) | 1.71 (1.52 to 1.92) | 739.17 (607.09 to 877.94) | 1.01 (0.84 to 1.19) | -1.78 (-2.11 to -1.45) | 0 |
| Central African Republic | 71.33 (46.51 to 98.21) | 5.33 (3.57 to 7.24) | 109.21 (71.92 to 160.09) | 3.94 (2.67 to 5.63) | -0.97 (-1.11 to -0.83) | 0 | 2.33 (1.55 to 3.15) | 0.19 (0.13 to 0.25) | 3.40 (2.27 to 4.91) | 0.14 (0.10 to 0.20) | -1 (-1.15 to -0.86) | 0 |
| Chad | 58.15 (40.29 to 77.68) | 2.00 (1.39 to 2.67) | 191.89 (132.18 to 265.56) | 3.05 (2.12 to 4.18) | 1.4 (1.23 to 1.57) | 0 | 2.26 (1.56 to 3.08) | 0.08 (0.06 to 0.11) | 6.82 (4.73 to 9.45) | 0.12 (0.08 to 0.17) | 1.35 (1.2 to 1.5) | 0 |
| Chile | 2095.54 (1818.16 to 2418.66) | 20.03 (17.28 to 23.17) | 4783.43 (4065.71 to 5475.89) | 19.06 (16.26 to 21.81) | -0.11 (-0.76 to 0.54) | 0.736 | 74.01 (63.58 to 85.87) | 0.73 (0.62 to 0.84) | 173.88 (144.26 to 205.42) | 0.68 (0.57 to 0.80) | -0.29 (-0.95 to 0.38) | 0.396 |
| China | 245845.02 (199027.08 to 299055.59) | 26.63 (21.68 to 32.39) | 601155.27 (450655.73 to 771764.47) | 27.67 (20.76 to 35.36) | 0.1 (-0.02 to 0.22) | 0.09 | 8330.59 (6830.28 to 10121.23) | 0.99 (0.82 to 1.20) | 23303.32 (17697.26 to 29826.23) | 1.09 (0.83 to 1.38) | 0.28 (0.12 to 0.45) | 0.001 |
| Colombia | 3023.39 (2667.72 to 3470.31) | 15.93 (13.99 to 18.33) | 4400.46 (3475.13 to 5554.83) | 7.91 (6.25 to 9.99) | -2.32 (-2.58 to -2.05) | 0 | 105.39 (92.18 to 120.86) | 0.59 (0.52 to 0.68) | 169.57 (133.36 to 215.63) | 0.31 (0.24 to 0.39) | -2.2 (-2.42 to -1.97) | 0 |
| Comoros | 14.53 (9.58 to 20.08) | 6.95 (4.67 to 9.48) | 29.02 (18.98 to 45.45) | 5.66 (3.79 to 8.73) | -0.7 (-0.88 to -0.52) | 0 | 0.54 (0.36 to 0.73) | 0.28 (0.19 to 0.38) | 1.11 (0.76 to 1.69) | 0.24 (0.17 to 0.36) | -0.58 (-0.72 to -0.43) | 0 |
| Congo | 79.12 (51.98 to 112.67) | 6.72 (4.48 to 9.45) | 237.94 (149.68 to 335.03) | 7.62 (4.85 to 10.70) | 0.47 (0.19 to 0.75) | 0.001 | 2.74 (1.85 to 3.81) | 0.25 (0.18 to 0.35) | 8.00 (5.06 to 11.21) | 0.30 (0.19 to 0.41) | 0.57 (0.39 to 0.76) | 0 |
| Cook Islands | 2.14 (1.67 to 2.57) | 15.99 (12.54 to 19.28) | 3.89 (3.01 to 4.98) | 14.94 (11.47 to 19.08) | -0.2 (-0.31 to -0.08) | 0.001 | 0.08 (0.06 to 0.09) | 0.61 (0.49 to 0.74) | 0.15 (0.12 to 0.19) | 0.57 (0.44 to 0.71) | -0.23 (-0.34 to -0.12) | 0 |
| Costa Rica | 242.49 (207.80 to 276.51) | 13.64 (11.64 to 15.61) | 704.72 (572.68 to 848.90) | 12.71 (10.34 to 15.32) | -0.3 (-1.13 to 0.54) | 0.488 | 9.56 (8.06 to 10.96) | 0.56 (0.47 to 0.64) | 28.95 (23.42 to 35.22) | 0.53 (0.43 to 0.64) | -0.15 (-0.98 to 0.68) | 0.723 |
| Croatia | 2795.93 (2433.68 to 3149.21) | 44.15 (38.48 to 49.89) | 3141.78 (2571.92 to 3725.48) | 38.40 (31.53 to 45.39) | -0.25 (-0.7 to 0.2) | 0.269 | 105.30 (91.92 to 120.79) | 1.73 (1.51 to 1.99) | 141.03 (116.65 to 169.20) | 1.58 (1.30 to 1.88) | -0.26 (-0.7 to 0.2) | 0.267 |
| Cuba | 2652.75 (2329.61 to 2988.75) | 25.94 (22.71 to 29.22) | 3938.71 (3234.43 to 4721.94) | 20.62 (16.93 to 24.67) | -0.83 (-1.41 to -0.26) | 0.005 | 109.78 (94.89 to 125.57) | 1.07 (0.92 to 1.22) | 152.59 (124.20 to 184.71) | 0.78 (0.64 to 0.94) | -1.1 (-1.67 to -0.53) | 0 |
| Cyprus | 191.82 (148.07 to 241.12) | 24.02 (18.44 to 30.16) | 528.80 (394.97 to 666.93) | 26.16 (19.66 to 32.96) | 0.24 (-0.03 to 0.51) | 0.078 | 7.98 (6.12 to 10.09) | 1.06 (0.80 to 1.34) | 22.57 (16.69 to 28.68) | 1.08 (0.81 to 1.37) | -0.02 (-0.26 to 0.22) | 0.861 |
| Czechia | 8303.37 (7323.60 to 9419.25) | 61.59 (54.21 to 69.51) | 9020.94 (7481.22 to 10845.39) | 46.36 (38.48 to 55.16) | -0.89 (-1.02 to -0.75) | 0 | 314.17 (274.24 to 357.16) | 2.27 (1.98 to 2.57) | 379.36 (311.12 to 459.42) | 1.81 (1.49 to 2.18) | -0.72 (-0.84 to -0.59) | 0 |
| Democratic People's Republic of Korea | 3070.08 (2148.46 to 4354.45) | 16.28 (11.49 to 23.06) | 5867.46 (3889.47 to 8436.57) | 16.70 (11.22 to 23.79) | 0.08 (0.05 to 0.11) | 0 | 96.66 (68.36 to 137.47) | 0.55 (0.40 to 0.78) | 191.57 (127.51 to 268.76) | 0.56 (0.37 to 0.78) | 0.02 (-0.01 to 0.05) | 0.133 |
| Democratic Republic of the Congo | 673.18 (477.51 to 900.29) | 3.81 (2.70 to 5.08) | 1370.55 (839.27 to 2072.27) | 3.16 (1.93 to 4.80) | -0.59 (-0.79 to -0.38) | 0 | 22.86 (16.15 to 30.65) | 0.14 (0.10 to 0.19) | 44.76 (27.31 to 67.85) | 0.12 (0.07 to 0.18) | -0.67 (-0.84 to -0.5) | 0 |
| Denmark | 3413.73 (3013.78 to 3880.78) | 45.33 (40.17 to 51.17) | 4568.71 (3950.26 to 5272.99) | 40.22 (34.69 to 46.01) | -0.39 (-1.15 to 0.37) | 0.311 | 153.06 (133.63 to 176.75) | 1.89 (1.66 to 2.17) | 224.55 (192.44 to 262.58) | 1.82 (1.57 to 2.11) | -0.03 (-0.68 to 0.62) | 0.923 |
| Djibouti | 11.88 (6.71 to 18.71) | 7.68 (4.48 to 11.83) | 67.00 (39.58 to 109.42) | 9.36 (5.77 to 14.77) | 0.64 (0.49 to 0.79) | 0 | 0.40 (0.23 to 0.62) | 0.30 (0.18 to 0.46) | 2.32 (1.42 to 3.68) | 0.39 (0.24 to 0.59) | 0.77 (0.65 to 0.89) | 0 |
| Dominica | 6.99 (5.49 to 8.79) | 12.13 (9.63 to 15.18) | 14.47 (10.68 to 19.73) | 16.62 (12.34 to 22.54) | 1.04 (0.96 to 1.12) | 0 | 0.29 (0.22 to 0.36) | 0.48 (0.37 to 0.61) | 0.55 (0.41 to 0.75) | 0.65 (0.48 to 0.87) | 0.97 (0.88 to 1.06) | 0 |
| Dominican Republic | 472.19 (364.66 to 595.33) | 12.50 (9.58 to 15.79) | 1892.71 (1457.74 to 2525.82) | 18.73 (14.45 to 25.02) | 1.4 (0.91 to 1.89) | 0 | 18.68 (14.20 to 23.62) | 0.54 (0.42 to 0.69) | 78.38 (59.93 to 104.90) | 0.80 (0.61 to 1.06) | 1.23 (0.7 to 1.77) | 0 |
| Ecuador | 515.17 (448.09 to 583.13) | 9.59 (8.32 to 10.89) | 1418.11 (1020.79 to 1882.46) | 8.55 (6.15 to 11.36) | -0.64 (-1.37 to 0.1) | 0.09 | 20.15 (17.43 to 22.96) | 0.39 (0.34 to 0.45) | 57.69 (41.90 to 76.07) | 0.35 (0.26 to 0.47) | -0.57 (-1.21 to 0.08) | 0.083 |
| Egypt | 2907.54 (2431.62 to 3450.16) | 9.34 (7.87 to 11.12) | 16038.82 (12630.55 to 20472.31) | 22.44 (17.90 to 28.45) | 2.84 (2.56 to 3.13) | 0 | 95.90 (80.64 to 114.38) | 0.35 (0.30 to 0.42) | 558.82 (445.21 to 714.65) | 0.90 (0.72 to 1.13) | 3.06 (2.82 to 3.3) | 0 |
| El Salvador | 122.59 (101.15 to 146.05) | 3.95 (3.24 to 4.72) | 462.56 (343.82 to 610.36) | 7.72 (5.75 to 10.19) | 2.43 (1.96 to 2.9) | 0 | 4.19 (3.39 to 4.98) | 0.14 (0.11 to 0.17) | 16.69 (12.41 to 22.14) | 0.28 (0.21 to 0.37) | 2.42 (2 to 2.85) | 0 |
| Equatorial Guinea | 11.67 (7.48 to 16.31) | 5.30 (3.48 to 7.34) | 52.32 (30.87 to 78.25) | 8.82 (5.33 to 12.89) | 1.69 (1.49 to 1.89) | 0 | 0.39 (0.26 to 0.53) | 0.19 (0.13 to 0.26) | 1.72 (1.03 to 2.50) | 0.33 (0.21 to 0.48) | 1.84 (1.65 to 2.04) | 0 |
| Eritrea | 45.57 (31.81 to 60.53) | 2.95 (2.11 to 3.90) | 83.65 (45.98 to 136.77) | 2.28 (1.30 to 3.64) | -0.87 (-1.05 to -0.69) | 0 | 1.32 (0.94 to 1.76) | 0.10 (0.07 to 0.13) | 2.46 (1.39 to 3.95) | 0.08 (0.04 to 0.12) | -0.77 (-0.91 to -0.62) | 0 |
| Estonia | 764.65 (672.21 to 871.26) | 37.83 (33.27 to 42.97) | 861.73 (697.65 to 1008.47) | 37.68 (30.63 to 44.05) | 0.06 (-0.52 to 0.64) | 0.842 | 26.93 (23.76 to 30.81) | 1.31 (1.16 to 1.50) | 34.86 (28.01 to 41.39) | 1.40 (1.14 to 1.64) | 0.25 (-0.34 to 0.84) | 0.406 |
| Eswatini | 33.04 (20.69 to 50.55) | 10.96 (6.93 to 16.66) | 75.19 (43.45 to 122.52) | 12.39 (7.28 to 19.95) | 0.44 (0.17 to 0.71) | 0.001 | 1.20 (0.75 to 1.81) | 0.45 (0.28 to 0.66) | 2.66 (1.58 to 4.21) | 0.50 (0.29 to 0.78) | 0.36 (0.16 to 0.55) | 0 |
| Ethiopia | 263.59 (127.69 to 444.06) | 1.18 (0.58 to 1.99) | 426.96 (252.53 to 688.81) | 0.93 (0.55 to 1.48) | -0.78 (-0.94 to -0.63) | 0 | 9.08 (4.44 to 15.29) | 0.04 (0.02 to 0.07) | 15.67 (9.31 to 24.77) | 0.04 (0.02 to 0.06) | -0.55 (-0.7 to -0.4) | 0 |
| Fiji | 46.98 (36.23 to 58.63) | 11.46 (8.87 to 14.30) | 99.02 (68.28 to 137.33) | 11.33 (7.87 to 15.61) | -0.04 (-0.18 to 0.11) | 0.617 | 1.55 (1.20 to 1.93) | 0.42 (0.32 to 0.53) | 3.44 (2.38 to 4.74) | 0.42 (0.29 to 0.58) | -0.02 (-0.15 to 0.12) | 0.822 |
| Finland | 2401.70 (2099.66 to 2725.22) | 35.68 (31.39 to 40.44) | 3115.27 (2613.87 to 3670.38) | 29.27 (24.86 to 33.92) | -0.67 (-1.21 to -0.12) | 0.018 | 92.60 (79.32 to 107.43) | 1.32 (1.14 to 1.52) | 136.78 (113.11 to 165.10) | 1.15 (0.96 to 1.36) | -0.35 (-0.85 to 0.16) | 0.179 |
| France | 24407.03 (21601.44 to 27341.20) | 32.35 (28.70 to 36.09) | 40002.30 (34115.53 to 46100.45) | 34.47 (29.84 to 39.40) | 0.22 (0.06 to 0.38) | 0.006 | 1001.67 (878.68 to 1140.86) | 1.25 (1.10 to 1.40) | 1722.04 (1444.92 to 2036.15) | 1.33 (1.13 to 1.54) | 0.21 (0.07 to 0.35) | 0.004 |
| Gabon | 37.63 (24.98 to 57.62) | 6.27 (4.19 to 9.54) | 112.40 (75.13 to 154.50) | 9.36 (6.26 to 12.80) | 1.34 (1.21 to 1.46) | 0 | 1.35 (0.90 to 2.03) | 0.23 (0.15 to 0.35) | 3.81 (2.55 to 5.21) | 0.35 (0.23 to 0.47) | 1.35 (1.23 to 1.47) | 0 |
| Gambia | 15.12 (10.88 to 20.49) | 3.91 (2.84 to 5.18) | 41.11 (28.42 to 55.70) | 3.84 (2.69 to 5.13) | 0.04 (-0.77 to 0.86) | 0.916 | 0.51 (0.37 to 0.68) | 0.14 (0.10 to 0.19) | 1.40 (0.99 to 1.87) | 0.14 (0.10 to 0.19) | 0.07 (-0.66 to 0.8) | 0.856 |
| Georgia | 692.12 (543.25 to 914.38) | 10.44 (8.20 to 13.82) | 1793.86 (1520.19 to 2101.36) | 32.72 (27.76 to 38.22) | 3.89 (2.63 to 5.16) | 0 | 22.93 (18.10 to 30.66) | 0.35 (0.27 to 0.46) | 66.39 (55.80 to 77.87) | 1.15 (0.97 to 1.35) | 4.21 (2.96 to 5.47) | 0 |
| Germany | 44613.11 (39219.54 to 49996.83) | 37.31 (33.00 to 41.79) | 61875.72 (53431.68 to 71022.81) | 37.19 (32.26 to 42.05) | -0.01 (-0.35 to 0.32) | 0.941 | 1863.55 (1608.17 to 2106.93) | 1.48 (1.28 to 1.67) | 2764.40 (2369.79 to 3235.18) | 1.50 (1.30 to 1.74) | 0.03 (-0.25 to 0.31) | 0.827 |
| Ghana | 127.88 (85.15 to 189.28) | 1.94 (1.28 to 2.83) | 767.28 (511.46 to 1175.60) | 4.30 (2.89 to 6.57) | 2.62 (2.49 to 2.75) | 0 | 4.62 (3.04 to 6.78) | 0.08 (0.05 to 0.12) | 28.10 (18.96 to 43.00) | 0.18 (0.12 to 0.27) | 2.67 (2.54 to 2.8) | 0 |
| Greece | 7121.59 (6461.69 to 7829.44) | 47.56 (43.32 to 52.18) | 9894.55 (8795.92 to 11148.66) | 50.02 (44.85 to 56.00) | 0.11 (-0.29 to 0.51) | 0.6 | 298.17 (269.39 to 330.34) | 1.94 (1.76 to 2.16) | 454.30 (395.27 to 519.26) | 2.01 (1.78 to 2.28) | 0.06 (-0.38 to 0.5) | 0.786 |
| Greenland | 47.45 (38.41 to 56.35) | 121.06 (99.10 to 143.98) | 65.80 (52.22 to 81.32) | 83.20 (66.05 to 102.38) | -1.15 (-1.27 to -1.04) | 0 | 1.57 (1.29 to 1.87) | 4.55 (3.68 to 5.45) | 2.34 (1.84 to 2.92) | 3.15 (2.46 to 4.00) | -1.06 (-1.31 to -0.81) | 0 |
| Grenada | 10.09 (8.21 to 12.23) | 15.80 (12.95 to 19.13) | 24.41 (19.23 to 29.97) | 19.73 (15.62 to 24.18) | 0.79 (0.36 to 1.22) | 0 | 0.40 (0.32 to 0.50) | 0.58 (0.47 to 0.71) | 0.89 (0.71 to 1.10) | 0.75 (0.59 to 0.93) | 0.89 (0.23 to 1.55) | 0.008 |
| Guam | 10.84 (9.06 to 12.93) | 12.05 (10.07 to 14.47) | 33.15 (26.64 to 40.58) | 15.81 (12.74 to 19.25) | 0.87 (0.19 to 1.56) | 0.012 | 0.36 (0.30 to 0.43) | 0.44 (0.36 to 0.53) | 1.11 (0.89 to 1.36) | 0.52 (0.42 to 0.64) | 0.56 (-0.19 to 1.31) | 0.142 |
| Guatemala | 147.08 (127.24 to 170.46) | 3.91 (3.38 to 4.56) | 636.21 (506.03 to 793.44) | 5.62 (4.44 to 7.01) | 0.84 (-0.24 to 1.93) | 0.129 | 5.17 (4.47 to 6.03) | 0.16 (0.14 to 0.19) | 24.62 (19.24 to 30.62) | 0.23 (0.18 to 0.28) | 0.82 (-0.26 to 1.9) | 0.137 |
| Guinea | 75.68 (54.95 to 101.76) | 2.20 (1.59 to 2.97) | 198.75 (138.80 to 287.69) | 3.29 (2.31 to 4.74) | 1.3 (1.15 to 1.44) | 0 | 2.83 (2.07 to 3.79) | 0.09 (0.06 to 0.12) | 7.13 (5.03 to 10.18) | 0.13 (0.09 to 0.18) | 1.27 (1.16 to 1.38) | 0 |
| Guinea-Bissau | 9.16 (5.21 to 13.63) | 2.10 (1.20 to 3.11) | 29.87 (19.41 to 41.93) | 3.39 (2.20 to 4.76) | 1.58 (1.46 to 1.71) | 0 | 0.31 (0.18 to 0.46) | 0.08 (0.04 to 0.11) | 0.94 (0.61 to 1.31) | 0.12 (0.08 to 0.17) | 1.49 (1.37 to 1.61) | 0 |
| Guyana | 35.57 (29.28 to 43.47) | 8.59 (7.07 to 10.51) | 80.96 (59.41 to 109.31) | 11.32 (8.32 to 15.25) | 1.14 (0.28 to 2) | 0.009 | 1.20 (0.99 to 1.46) | 0.31 (0.25 to 0.38) | 2.70 (1.99 to 3.65) | 0.40 (0.30 to 0.54) | 1.03 (0.25 to 1.82) | 0.01 |
| Haiti | 292.60 (192.50 to 406.01) | 8.08 (5.40 to 11.20) | 445.62 (300.21 to 629.32) | 5.52 (3.72 to 7.90) | -1.16 (-1.22 to -1.11) | 0 | 9.66 (6.53 to 13.41) | 0.29 (0.20 to 0.40) | 15.28 (10.32 to 21.92) | 0.21 (0.14 to 0.31) | -0.92 (-0.97 to -0.87) | 0 |
| Honduras | 166.37 (132.63 to 208.53) | 7.62 (6.08 to 9.56) | 785.36 (570.41 to 1090.09) | 11.79 (8.61 to 16.34) | 1.49 (1.22 to 1.75) | 0 | 5.92 (4.70 to 7.37) | 0.29 (0.23 to 0.36) | 29.74 (21.73 to 41.08) | 0.47 (0.35 to 0.66) | 1.63 (1.4 to 1.87) | 0 |
| Hungary | 7269.83 (6385.35 to 8261.05) | 51.33 (45.13 to 58.22) | 7557.80 (6190.07 to 9009.42) | 44.40 (36.66 to 52.75) | -0.5 (-1.06 to 0.06) | 0.079 | 257.39 (225.71 to 294.48) | 1.76 (1.55 to 2.01) | 291.41 (237.73 to 348.14) | 1.60 (1.31 to 1.91) | -0.36 (-0.92 to 0.21) | 0.213 |
| Iceland | 114.67 (99.25 to 129.59) | 41.74 (36.36 to 47.18) | 146.97 (123.78 to 175.82) | 26.94 (22.72 to 31.94) | -1.35 (-2.04 to -0.65) | 0 | 5.00 (4.31 to 5.70) | 1.75 (1.51 to 1.99) | 6.64 (5.44 to 8.12) | 1.14 (0.94 to 1.38) | -1.29 (-2.13 to -0.44) | 0.003 |
| India | 22394.59 (17208.82 to 27826.46) | 4.41 (3.37 to 5.50) | 49333.61 (40578.06 to 58295.77) | 3.96 (3.26 to 4.69) | -0.34 (-0.56 to -0.12) | 0.002 | 777.13 (593.82 to 974.27) | 0.17 (0.13 to 0.21) | 1901.14 (1572.06 to 2252.49) | 0.16 (0.13 to 0.19) | -0.1 (-0.32 to 0.12) | 0.366 |
| Indonesia | 9601.44 (8054.89 to 11754.63) | 8.70 (7.22 to 10.63) | 39919.25 (28803.38 to 53235.75) | 14.73 (10.69 to 19.53) | 1.71 (1.61 to 1.82) | 0 | 322.29 (267.03 to 395.09) | 0.33 (0.27 to 0.40) | 1391.84 (1006.05 to 1834.56) | 0.58 (0.41 to 0.76) | 1.83 (1.71 to 1.96) | 0 |
| Iran (Islamic Republic of) | 1717.60 (1363.41 to 2111.30) | 5.80 (4.64 to 7.14) | 8395.01 (7084.09 to 9774.65) | 9.96 (8.40 to 11.64) | 1.72 (1.59 to 1.85) | 0 | 58.17 (45.95 to 71.97) | 0.22 (0.18 to 0.27) | 303.57 (254.04 to 355.15) | 0.39 (0.32 to 0.46) | 1.86 (1.72 to 2) | 0 |
| Iraq | 1311.66 (883.53 to 1808.76) | 15.89 (10.77 to 21.86) | 5213.42 (3645.61 to 6863.19) | 20.09 (14.06 to 26.06) | 0.77 (0.57 to 0.97) | 0 | 47.48 (32.18 to 65.10) | 0.61 (0.41 to 0.84) | 189.39 (132.54 to 245.52) | 0.83 (0.59 to 1.09) | 1.04 (0.8 to 1.28) | 0 |
| Ireland | 2039.34 (1854.32 to 2252.36) | 50.55 (45.96 to 55.81) | 1795.35 (1505.56 to 2091.25) | 23.46 (19.71 to 27.13) | -2.43 (-3.05 to -1.81) | 0 | 91.85 (83.02 to 102.46) | 2.22 (2.00 to 2.47) | 82.69 (68.43 to 98.11) | 1.03 (0.86 to 1.21) | -2.43 (-2.89 to -1.98) | 0 |
| Israel | 1756.48 (1545.75 to 1974.94) | 37.23 (32.99 to 41.69) | 3119.62 (2698.76 to 3582.77) | 26.98 (23.50 to 30.78) | -0.98 (-1.53 to -0.43) | 0.001 | 73.54 (63.13 to 84.00) | 1.52 (1.32 to 1.74) | 136.72 (115.31 to 159.55) | 1.12 (0.95 to 1.30) | -0.98 (-1.52 to -0.44) | 0 |
| Italy | 35702.81 (32407.30 to 39038.09) | 42.21 (38.39 to 46.03) | 35769.01 (30900.87 to 40871.71) | 28.93 (25.53 to 32.70) | -1.26 (-1.49 to -1.04) | 0 | 1447.69 (1298.48 to 1604.14) | 1.64 (1.48 to 1.81) | 1639.79 (1370.31 to 1922.96) | 1.18 (1.01 to 1.36) | -1.08 (-1.25 to -0.92) | 0 |
| Jamaica | 170.31 (141.29 to 207.68) | 10.04 (8.31 to 12.29) | 326.98 (236.34 to 453.44) | 10.63 (7.69 to 14.72) | 0.53 (-0.81 to 1.88) | 0.44 | 6.89 (5.65 to 8.28) | 0.39 (0.32 to 0.47) | 12.80 (9.36 to 17.78) | 0.42 (0.31 to 0.58) | 0.55 (-0.7 to 1.81) | 0.393 |
| Japan | 73734.79 (67795.23 to 79962.27) | 42.55 (39.14 to 46.18) | 86875.92 (74807.32 to 98471.26) | 30.38 (26.84 to 33.80) | -1.11 (-1.14 to -1.07) | 0 | 2961.77 (2705.95 to 3243.36) | 1.73 (1.58 to 1.89) | 4368.66 (3642.44 to 5115.02) | 1.27 (1.09 to 1.43) | -1.05 (-1.12 to -0.98) | 0 |
| Jordan | 178.47 (140.40 to 221.88) | 11.97 (9.42 to 14.85) | 1316.36 (959.40 to 1790.79) | 15.44 (11.26 to 20.67) | 0.87 (0.54 to 1.2) | 0 | 6.13 (4.84 to 7.59) | 0.47 (0.37 to 0.59) | 45.93 (33.55 to 61.50) | 0.63 (0.46 to 0.83) | 0.96 (0.58 to 1.33) | 0 |
| Kazakhstan | 2703.63 (2018.99 to 3839.63) | 19.27 (14.38 to 27.43) | 4067.13 (3298.28 to 4853.93) | 20.05 (16.31 to 23.80) | 0.15 (-0.33 to 0.64) | 0.541 | 84.80 (63.80 to 118.53) | 0.62 (0.47 to 0.87) | 131.68 (108.11 to 156.73) | 0.67 (0.55 to 0.80) | 0.25 (-0.33 to 0.83) | 0.398 |
| Kenya | 300.64 (196.77 to 436.81) | 3.50 (2.29 to 5.07) | 1246.72 (933.55 to 1632.67) | 4.94 (3.70 to 6.40) | 1.14 (0.92 to 1.35) | 0 | 11.22 (7.32 to 16.17) | 0.14 (0.09 to 0.21) | 44.12 (33.00 to 57.10) | 0.20 (0.15 to 0.25) | 1.04 (0.83 to 1.25) | 0 |
| Kiribati | 2.10 (1.68 to 2.59) | 5.23 (4.18 to 6.41) | 5.20 (3.61 to 7.35) | 6.33 (4.42 to 8.82) | 0.63 (0.57 to 0.69) | 0 | 0.07 (0.06 to 0.09) | 0.20 (0.16 to 0.24) | 0.18 (0.12 to 0.24) | 0.24 (0.17 to 0.33) | 0.66 (0.6 to 0.72) | 0 |
| Kuwait | 78.74 (65.77 to 92.20) | 10.96 (9.09 to 12.79) | 483.02 (367.35 to 611.99) | 13.73 (10.53 to 17.54) | 0.9 (-1 to 2.84) | 0.356 | 2.57 (2.14 to 2.99) | 0.43 (0.35 to 0.51) | 16.65 (12.93 to 21.20) | 0.59 (0.46 to 0.77) | 1.01 (-1.42 to 3.5) | 0.418 |
| Kyrgyzstan | 543.13 (434.48 to 659.45) | 17.30 (13.90 to 20.89) | 1182.83 (935.54 to 1494.86) | 21.11 (16.63 to 26.51) | 0.37 (-1.07 to 1.84) | 0.616 | 18.01 (14.53 to 21.87) | 0.59 (0.48 to 0.71) | 38.29 (30.10 to 48.22) | 0.74 (0.59 to 0.93) | 0.49 (-0.95 to 1.96) | 0.508 |
| Lao People's Democratic Republic | 261.41 (154.43 to 410.27) | 11.51 (6.81 to 17.97) | 671.32 (465.23 to 897.20) | 13.21 (9.26 to 17.74) | 0.45 (0.39 to 0.52) | 0 | 9.03 (5.34 to 14.11) | 0.43 (0.26 to 0.67) | 23.71 (16.71 to 31.85) | 0.52 (0.37 to 0.69) | 0.64 (0.55 to 0.73) | 0 |
| Latvia | 1384.95 (1210.87 to 1571.20) | 38.98 (34.05 to 44.28) | 1480.55 (1215.20 to 1777.47) | 45.85 (37.70 to 54.88) | 0.45 (-0.21 to 1.11) | 0.18 | 49.10 (42.66 to 56.22) | 1.36 (1.18 to 1.56) | 55.19 (45.07 to 66.53) | 1.57 (1.29 to 1.89) | 0.42 (-0.21 to 1.05) | 0.19 |
| Lebanon | 381.12 (239.14 to 512.92) | 16.47 (10.50 to 22.09) | 1276.68 (918.05 to 1733.99) | 21.95 (15.80 to 29.77) | 0.92 (0.65 to 1.18) | 0 | 14.04 (9.02 to 18.98) | 0.65 (0.42 to 0.87) | 55.49 (39.40 to 75.84) | 0.92 (0.65 to 1.26) | 1.11 (0.89 to 1.33) | 0 |
| Lesotho | 70.16 (50.39 to 94.69) | 8.01 (5.78 to 10.78) | 253.02 (163.37 to 352.68) | 21.44 (13.97 to 30.04) | 3.28 (2.94 to 3.62) | 0 | 2.67 (1.95 to 3.61) | 0.32 (0.24 to 0.44) | 8.65 (5.68 to 12.20) | 0.80 (0.53 to 1.12) | 3.09 (2.78 to 3.39) | 0 |
| Liberia | 33.00 (21.82 to 46.56) | 2.77 (1.84 to 3.88) | 87.34 (47.28 to 137.67) | 3.47 (1.92 to 5.42) | 0.68 (0.44 to 0.92) | 0 | 1.19 (0.79 to 1.67) | 0.10 (0.07 to 0.14) | 2.81 (1.55 to 4.37) | 0.13 (0.07 to 0.20) | 0.59 (0.39 to 0.78) | 0 |
| Libya | 390.03 (259.91 to 542.24) | 19.28 (12.99 to 26.83) | 1701.72 (1141.32 to 2300.33) | 28.19 (18.99 to 38.30) | 1.22 (1.06 to 1.38) | 0 | 13.97 (9.54 to 19.01) | 0.75 (0.52 to 1.02) | 58.38 (39.91 to 78.85) | 1.11 (0.76 to 1.49) | 1.26 (1.07 to 1.45) | 0 |
| Lithuania | 1667.71 (1450.15 to 1860.87) | 37.29 (32.30 to 41.59) | 1917.31 (1605.38 to 2224.44) | 39.98 (33.56 to 46.16) | 0.2 (-0.48 to 0.89) | 0.566 | 59.09 (51.30 to 66.07) | 1.31 (1.14 to 1.46) | 73.11 (60.66 to 85.38) | 1.40 (1.17 to 1.63) | 0.21 (-0.43 to 0.86) | 0.522 |
| Luxembourg | 226.52 (197.88 to 254.96) | 43.16 (37.86 to 48.51) | 291.44 (243.04 to 345.81) | 28.69 (23.95 to 33.92) | -1.35 (-1.73 to -0.98) | 0 | 8.94 (7.71 to 10.21) | 1.65 (1.43 to 1.88) | 12.64 (10.40 to 15.29) | 1.20 (0.99 to 1.45) | -1.07 (-1.47 to -0.66) | 0 |
| Madagascar | 210.01 (144.15 to 296.72) | 3.91 (2.72 to 5.52) | 261.19 (159.47 to 420.25) | 2.02 (1.28 to 3.22) | -2.12 (-2.2 to -2.04) | 0 | 7.53 (5.22 to 10.65) | 0.16 (0.11 to 0.22) | 8.70 (5.46 to 13.82) | 0.08 (0.05 to 0.13) | -2.08 (-2.16 to -2) | 0 |
| Malawi | 66.40 (50.07 to 86.83) | 1.65 (1.26 to 2.14) | 170.87 (113.80 to 244.67) | 2.16 (1.46 to 3.06) | 0.9 (0.74 to 1.05) | 0 | 2.50 (1.89 to 3.26) | 0.07 (0.05 to 0.09) | 6.24 (4.21 to 8.81) | 0.09 (0.06 to 0.12) | 0.82 (0.66 to 0.98) | 0 |
| Malaysia | 734.09 (597.57 to 887.49) | 7.53 (6.14 to 9.13) | 2677.09 (2224.15 to 3225.03) | 9.01 (7.46 to 10.87) | 0.7 (0.43 to 0.97) | 0 | 27.25 (22.10 to 32.67) | 0.30 (0.25 to 0.37) | 101.86 (83.57 to 122.49) | 0.37 (0.30 to 0.44) | 0.61 (0.33 to 0.89) | 0 |
| Maldives | 9.10 (5.58 to 13.51) | 9.71 (6.28 to 14.33) | 25.92 (19.23 to 34.03) | 7.21 (5.41 to 9.32) | -1.06 (-1.37 to -0.75) | 0 | 0.33 (0.21 to 0.49) | 0.42 (0.27 to 0.60) | 1.02 (0.77 to 1.31) | 0.33 (0.25 to 0.43) | -0.79 (-1.07 to -0.51) | 0 |
| Mali | 147.97 (112.78 to 189.04) | 3.52 (2.67 to 4.47) | 522.13 (374.03 to 717.31) | 5.68 (4.08 to 7.71) | 1.58 (1.47 to 1.69) | 0 | 5.42 (4.10 to 6.91) | 0.15 (0.11 to 0.18) | 19.68 (14.14 to 26.70) | 0.24 (0.18 to 0.33) | 1.71 (1.59 to 1.83) | 0 |
| Malta | 163.23 (141.41 to 185.07) | 37.86 (32.87 to 42.87) | 247.30 (208.19 to 296.55) | 30.81 (26.23 to 36.56) | -0.62 (-0.89 to -0.35) | 0 | 6.43 (5.51 to 7.40) | 1.49 (1.28 to 1.72) | 10.24 (8.37 to 12.39) | 1.15 (0.96 to 1.37) | -0.79 (-1.05 to -0.52) | 0 |
| Marshall Islands | 1.35 (1.05 to 1.74) | 7.48 (5.85 to 9.62) | 4.87 (3.24 to 7.10) | 11.45 (7.66 to 16.53) | 1.39 (1.31 to 1.47) | 0 | 0.04 (0.03 to 0.06) | 0.27 (0.21 to 0.35) | 0.15 (0.10 to 0.22) | 0.41 (0.28 to 0.59) | 1.43 (1.25 to 1.61) | 0 |
| Mauritania | 43.72 (33.08 to 57.52) | 4.10 (3.09 to 5.42) | 118.27 (78.52 to 169.76) | 5.01 (3.36 to 7.05) | 0.66 (0.3 to 1.02) | 0 | 1.52 (1.14 to 1.97) | 0.15 (0.11 to 0.20) | 4.13 (2.79 to 5.68) | 0.19 (0.13 to 0.26) | 0.76 (0.51 to 1) | 0 |
| Mauritius | 138.81 (124.24 to 153.73) | 17.59 (15.77 to 19.50) | 362.14 (319.49 to 403.32) | 19.00 (16.81 to 21.08) | 0.2 (-0.89 to 1.31) | 0.719 | 4.86 (4.36 to 5.44) | 0.66 (0.59 to 0.74) | 13.54 (11.99 to 15.08) | 0.72 (0.64 to 0.80) | 0.19 (-0.92 to 1.32) | 0.735 |
| Mexico | 9046.35 (8118.74 to 10053.85) | 20.66 (18.51 to 23.01) | 13988.78 (11579.72 to 16630.84) | 10.70 (8.84 to 12.75) | -2.11 (-2.44 to -1.78) | 0 | 352.39 (314.06 to 393.59) | 0.88 (0.78 to 0.99) | 555.06 (458.93 to 658.32) | 0.44 (0.37 to 0.52) | -2.2 (-2.45 to -1.94) | 0 |
| Micronesia (Federated States of) | 7.98 (5.87 to 10.67) | 15.42 (11.35 to 20.41) | 19.04 (13.16 to 26.31) | 21.44 (14.91 to 29.20) | 1.07 (1.04 to 1.09) | 0 | 0.27 (0.20 to 0.36) | 0.55 (0.41 to 0.73) | 0.62 (0.43 to 0.85) | 0.76 (0.54 to 1.02) | 1.04 (0.99 to 1.09) | 0 |
| Monaco | 30.59 (21.51 to 41.74) | 52.15 (37.11 to 72.21) | 38.14 (25.51 to 55.65) | 46.50 (30.22 to 68.53) | -0.38 (-0.42 to -0.33) | 0 | 1.35 (0.93 to 1.83) | 2.03 (1.42 to 2.78) | 1.74 (1.17 to 2.55) | 1.86 (1.25 to 2.72) | -0.29 (-0.33 to -0.25) | 0 |
| Mongolia | 67.46 (49.68 to 90.07) | 6.09 (4.48 to 8.09) | 964.04 (686.60 to 1296.08) | 34.90 (24.90 to 46.48) | 5.74 (5.45 to 6.03) | 0 | 2.31 (1.71 to 3.07) | 0.21 (0.16 to 0.29) | 30.91 (22.03 to 41.34) | 1.24 (0.88 to 1.66) | 5.74 (5.42 to 6.06) | 0 |
| Montenegro | 307.77 (246.68 to 385.71) | 47.05 (37.52 to 59.06) | 505.33 (393.72 to 642.46) | 51.13 (39.76 to 64.94) | 0.33 (-0.01 to 0.67) | 0.057 | 10.70 (8.59 to 13.47) | 1.68 (1.33 to 2.11) | 19.48 (15.07 to 24.79) | 1.93 (1.49 to 2.46) | 0.7 (0.24 to 1.16) | 0.003 |
| Morocco | 496.10 (378.45 to 630.31) | 3.29 (2.51 to 4.19) | 1566.02 (1135.83 to 2047.29) | 4.19 (3.05 to 5.52) | 0.78 (0.65 to 0.91) | 0 | 17.47 (13.26 to 22.33) | 0.12 (0.09 to 0.16) | 56.15 (40.65 to 74.66) | 0.16 (0.11 to 0.21) | 0.82 (0.68 to 0.95) | 0 |
| Mozambique | 65.72 (50.13 to 84.45) | 1.10 (0.84 to 1.40) | 173.57 (130.05 to 224.69) | 1.50 (1.14 to 1.94) | 1.04 (0.94 to 1.15) | 0 | 2.57 (1.96 to 3.27) | 0.05 (0.04 to 0.06) | 6.41 (4.86 to 8.30) | 0.06 (0.05 to 0.08) | 0.89 (0.79 to 0.99) | 0 |
| Myanmar | 3717.70 (2366.88 to 5665.64) | 15.06 (9.76 to 22.84) | 5669.86 (4016.64 to 7735.00) | 11.03 (7.87 to 14.88) | -1 (-1.08 to -0.93) | 0 | 134.20 (88.15 to 201.88) | 0.59 (0.40 to 0.88) | 218.20 (155.42 to 295.82) | 0.46 (0.33 to 0.61) | -0.81 (-0.88 to -0.74) | 0 |
| Namibia | 27.32 (20.95 to 35.11) | 4.13 (3.19 to 5.23) | 63.59 (44.92 to 83.84) | 4.51 (3.24 to 5.84) | 0.32 (0.12 to 0.53) | 0.002 | 1.05 (0.81 to 1.36) | 0.18 (0.14 to 0.23) | 2.48 (1.79 to 3.23) | 0.20 (0.15 to 0.25) | 0.36 (0.16 to 0.55) | 0 |
| Nauru | 1.22 (0.73 to 1.91) | 22.78 (13.84 to 35.28) | 1.70 (0.99 to 2.47) | 25.05 (14.89 to 36.23) | 0.32 (0.21 to 0.43) | 0 | 0.04 (0.02 to 0.06) | 0.82 (0.51 to 1.26) | 0.05 (0.03 to 0.08) | 0.88 (0.53 to 1.26) | 0.21 (0.1 to 0.32) | 0 |
| Nepal | 512.41 (269.16 to 754.93) | 5.28 (2.75 to 7.70) | 1225.12 (708.37 to 1843.36) | 5.19 (3.00 to 7.84) | -0.05 (-0.15 to 0.06) | 0.36 | 19.20 (9.95 to 28.11) | 0.22 (0.11 to 0.32) | 51.52 (29.91 to 77.98) | 0.23 (0.14 to 0.36) | 0.19 (0.09 to 0.28) | 0 |
| Netherlands | 9467.82 (8513.58 to 10488.11) | 49.35 (44.58 to 54.63) | 10645.38 (9154.57 to 12350.55) | 32.10 (27.76 to 36.80) | -1.41 (-1.68 to -1.14) | 0 | 405.41 (359.64 to 454.70) | 2.03 (1.81 to 2.28) | 498.77 (418.01 to 588.09) | 1.39 (1.18 to 1.63) | -1.24 (-1.65 to -0.82) | 0 |
| New Zealand | 1174.69 (1027.04 to 1329.15) | 30.38 (26.65 to 34.31) | 1785.69 (1514.63 to 2087.33) | 22.33 (19.12 to 25.96) | -1.05 (-1.68 to -0.42) | 0.001 | 48.70 (42.17 to 56.39) | 1.23 (1.06 to 1.42) | 79.00 (65.11 to 94.69) | 0.93 (0.78 to 1.11) | -0.95 (-1.53 to -0.37) | 0.001 |
| Nicaragua | 76.42 (61.13 to 93.75) | 4.61 (3.67 to 5.68) | 327.58 (252.77 to 436.44) | 6.35 (4.88 to 8.46) | 1.14 (0.72 to 1.56) | 0 | 2.69 (2.15 to 3.35) | 0.18 (0.14 to 0.22) | 12.07 (9.25 to 16.08) | 0.25 (0.19 to 0.33) | 1.09 (0.6 to 1.58) | 0 |
| Niger | 33.03 (21.76 to 44.77) | 1.11 (0.74 to 1.51) | 106.05 (61.30 to 160.80) | 1.23 (0.73 to 1.84) | 0.34 (0.13 to 0.55) | 0.002 | 1.17 (0.78 to 1.59) | 0.05 (0.03 to 0.06) | 3.89 (2.32 to 5.79) | 0.05 (0.03 to 0.08) | 0.54 (0.38 to 0.7) | 0 |
| Nigeria | 230.00 (161.73 to 302.45) | 0.51 (0.36 to 0.66) | 475.71 (337.16 to 642.21) | 0.47 (0.34 to 0.62) | -0.26 (-0.41 to -0.12) | 0 | 8.82 (6.22 to 11.53) | 0.02 (0.02 to 0.03) | 16.80 (12.33 to 22.38) | 0.02 (0.01 to 0.03) | -0.44 (-0.57 to -0.3) | 0 |
| Niue | 0.22 (0.15 to 0.29) | 10.45 (7.36 to 14.35) | 0.36 (0.26 to 0.48) | 15.75 (11.44 to 21.39) | 1.32 (1.26 to 1.37) | 0 | 0.01 (0.01 to 0.01) | 0.38 (0.27 to 0.51) | 0.01 (0.01 to 0.02) | 0.58 (0.44 to 0.79) | 1.4 (1.33 to 1.47) | 0 |
| North Macedonia | 760.26 (612.79 to 942.90) | 37.82 (30.64 to 46.92) | 1450.89 (1081.14 to 1924.22) | 42.31 (31.66 to 55.68) | 0.36 (0.09 to 0.62) | 0.009 | 26.86 (21.87 to 33.50) | 1.40 (1.15 to 1.76) | 55.47 (41.64 to 72.48) | 1.65 (1.25 to 2.13) | 0.56 (0.31 to 0.81) | 0 |
| Northern Mariana Islands | 3.53 (2.62 to 4.69) | 16.06 (12.43 to 21.00) | 14.45 (11.70 to 17.75) | 23.40 (19.18 to 28.48) | 1.23 (0.8 to 1.66) | 0 | 0.11 (0.08 to 0.14) | 0.60 (0.47 to 0.77) | 0.49 (0.40 to 0.61) | 0.88 (0.72 to 1.07) | 1.23 (0.78 to 1.69) | 0 |
| Norway | 2575.12 (2309.48 to 2877.47) | 42.07 (38.17 to 46.48) | 2047.45 (1762.56 to 2308.28) | 22.57 (19.75 to 25.37) | -2 (-2.2 to -1.8) | 0 | 114.23 (99.83 to 130.11) | 1.70 (1.51 to 1.92) | 91.27 (76.13 to 105.13) | 0.93 (0.78 to 1.06) | -1.87 (-2.12 to -1.62) | 0 |
| Oman | 26.11 (17.92 to 36.99) | 3.41 (2.33 to 4.92) | 93.74 (64.07 to 132.27) | 3.81 (2.65 to 5.24) | 0.37 (-0.2 to 0.95) | 0.2 | 0.89 (0.61 to 1.26) | 0.13 (0.09 to 0.19) | 3.08 (2.14 to 4.22) | 0.15 (0.11 to 0.21) | 0.57 (0.04 to 1.11) | 0.034 |
| Pakistan | 2561.33 (2028.47 to 3129.28) | 4.41 (3.49 to 5.40) | 5935.58 (4128.32 to 8609.72) | 4.56 (3.20 to 6.62) | 0.11 (0 to 0.21) | 0.047 | 98.68 (78.09 to 121.31) | 0.18 (0.14 to 0.22) | 216.53 (153.30 to 313.90) | 0.19 (0.13 to 0.27) | 0.1 (-0.01 to 0.2) | 0.068 |
| Palau | 1.38 (1.00 to 1.87) | 13.50 (9.93 to 18.07) | 3.26 (2.36 to 4.44) | 12.99 (9.59 to 17.52) | -0.14 (-0.27 to 0) | 0.046 | 0.05 (0.04 to 0.07) | 0.53 (0.39 to 0.71) | 0.12 (0.09 to 0.16) | 0.53 (0.40 to 0.72) | -0.01 (-0.15 to 0.13) | 0.908 |
| Palestine | 155.61 (106.56 to 214.48) | 17.58 (12.15 to 24.04) | 553.09 (441.62 to 686.71) | 19.78 (15.80 to 24.40) | 0.34 (0.1 to 0.57) | 0.005 | 6.12 (4.32 to 8.36) | 0.74 (0.53 to 1.01) | 19.98 (15.94 to 24.68) | 0.82 (0.65 to 1.00) | 0.25 (0 to 0.49) | 0.052 |
| Panama | 85.34 (73.90 to 96.33) | 5.64 (4.87 to 6.38) | 273.07 (202.64 to 349.27) | 6.20 (4.60 to 7.93) | 0.28 (-0.08 to 0.64) | 0.131 | 3.39 (2.91 to 3.87) | 0.23 (0.20 to 0.27) | 11.67 (8.68 to 15.17) | 0.26 (0.20 to 0.34) | 0.44 (-0.44 to 1.32) | 0.328 |
| Papua New Guinea | 111.42 (71.44 to 172.92) | 5.25 (3.39 to 8.04) | 356.87 (231.09 to 554.54) | 5.76 (3.80 to 8.82) | 0.32 (0.11 to 0.54) | 0.003 | 3.59 (2.31 to 5.53) | 0.19 (0.12 to 0.29) | 11.26 (7.39 to 17.26) | 0.21 (0.14 to 0.31) | 0.3 (0.13 to 0.48) | 0.001 |
| Paraguay | 267.28 (217.91 to 323.34) | 11.93 (9.73 to 14.38) | 1173.53 (861.71 to 1588.42) | 19.91 (14.60 to 26.84) | 1.79 (1.65 to 1.92) | 0 | 11.11 (8.96 to 13.38) | 0.52 (0.42 to 0.63) | 49.29 (35.96 to 66.02) | 0.88 (0.63 to 1.17) | 1.74 (1.3 to 2.17) | 0 |
| Peru | 935.87 (709.55 to 1217.60) | 7.63 (5.83 to 10.09) | 2765.00 (1912.56 to 3914.51) | 8.19 (5.65 to 11.54) | 0.35 (-0.51 to 1.23) | 0.426 | 36.11 (27.62 to 47.78) | 0.31 (0.24 to 0.41) | 113.14 (78.21 to 160.22) | 0.34 (0.24 to 0.48) | 0.42 (-0.44 to 1.29) | 0.335 |
| Philippines | 4428.28 (3670.58 to 5312.37) | 13.22 (10.87 to 15.99) | 12786.89 (10163.88 to 15920.52) | 14.11 (11.34 to 17.47) | 0.21 (0.13 to 0.3) | 0 | 146.72 (120.85 to 177.59) | 0.51 (0.42 to 0.62) | 446.27 (358.81 to 550.90) | 0.54 (0.44 to 0.66) | 0.26 (0.16 to 0.36) | 0 |
| Poland | 22665.55 (20889.25 to 24714.29) | 51.73 (47.76 to 56.37) | 24868.29 (21626.62 to 28215.43) | 37.56 (32.74 to 42.51) | -1.09 (-1.25 to -0.93) | 0 | 793.20 (722.96 to 869.26) | 1.80 (1.64 to 1.97) | 980.08 (848.15 to 1123.72) | 1.40 (1.21 to 1.60) | -0.89 (-1.02 to -0.75) | 0 |
| Portugal | 3217.15 (2829.46 to 3574.13) | 23.84 (21.08 to 26.44) | 4136.14 (3588.98 to 4729.42) | 21.39 (18.51 to 24.30) | -0.37 (-0.76 to 0.03) | 0.068 | 122.91 (107.65 to 137.91) | 0.88 (0.77 to 0.99) | 160.99 (137.15 to 186.79) | 0.76 (0.65 to 0.87) | -0.52 (-0.84 to -0.2) | 0.002 |
| Puerto Rico | 394.83 (330.95 to 463.52) | 10.88 (9.12 to 12.81) | 939.86 (732.35 to 1206.69) | 15.69 (12.28 to 19.99) | 1.21 (0.27 to 2.15) | 0.011 | 16.58 (13.67 to 19.76) | 0.45 (0.38 to 0.54) | 41.12 (31.44 to 53.11) | 0.60 (0.47 to 0.77) | 0.95 (-0.02 to 1.92) | 0.055 |
| Qatar | 22.83 (16.68 to 30.84) | 16.22 (11.92 to 21.26) | 176.78 (118.07 to 262.05) | 12.56 (8.17 to 19.48) | -0.85 (-1.61 to -0.07) | 0.032 | 0.70 (0.51 to 0.93) | 0.67 (0.49 to 0.87) | 5.19 (3.41 to 7.95) | 0.53 (0.34 to 0.80) | -0.94 (-1.96 to 0.09) | 0.073 |
| Republic of Korea | 14263.82 (11700.85 to 17179.39) | 43.05 (35.52 to 51.82) | 22546.26 (17517.32 to 28180.63) | 24.08 (18.74 to 30.09) | -1.94 (-2.04 to -1.83) | 0 | 496.32 (410.15 to 599.40) | 1.69 (1.39 to 2.04) | 1042.54 (801.80 to 1329.14) | 1.10 (0.85 to 1.40) | -1.42 (-1.6 to -1.24) | 0 |
| Republic of Moldova | 1484.23 (1276.00 to 1723.26) | 31.80 (27.40 to 36.71) | 2117.14 (1790.60 to 2433.52) | 36.59 (31.13 to 41.92) | 0.39 (-0.27 to 1.05) | 0.253 | 49.01 (41.54 to 56.94) | 1.07 (0.90 to 1.24) | 73.40 (61.46 to 84.71) | 1.23 (1.04 to 1.42) | 0.12 (-0.3 to 0.56) | 0.569 |
| Romania | 9573.00 (8377.15 to 10810.19) | 33.25 (29.21 to 37.50) | 13760.22 (11336.14 to 16144.12) | 42.96 (35.39 to 50.41) | 0.96 (0.37 to 1.56) | 0.001 | 321.09 (280.66 to 363.44) | 1.12 (0.97 to 1.26) | 503.55 (413.15 to 589.63) | 1.47 (1.21 to 1.73) | 0.9 (0.34 to 1.46) | 0.001 |
| Russian Federation | 73473.22 (67769.39 to 79624.76) | 39.93 (36.82 to 43.13) | 87999.48 (75917.30 to 100420.10) | 38.40 (33.18 to 43.67) | 0.09 (-0.54 to 0.72) | 0.783 | 2370.60 (2183.22 to 2566.87) | 1.28 (1.18 to 1.39) | 3110.57 (2685.27 to 3557.65) | 1.31 (1.13 to 1.50) | 0.28 (-0.3 to 0.86) | 0.345 |
| Rwanda | 322.10 (245.34 to 415.49) | 10.96 (8.42 to 14.13) | 606.94 (405.10 to 919.90) | 9.47 (6.44 to 14.21) | -0.47 (-0.65 to -0.3) | 0 | 12.04 (9.26 to 15.53) | 0.46 (0.35 to 0.58) | 23.86 (16.24 to 35.92) | 0.42 (0.29 to 0.63) | -0.21 (-0.31 to -0.11) | 0 |
| Saint Kitts and Nevis | 3.61 (2.74 to 4.52) | 10.48 (8.01 to 12.95) | 7.75 (5.79 to 10.11) | 10.28 (7.71 to 13.45) | 0.05 (-0.61 to 0.72) | 0.876 | 0.15 (0.11 to 0.19) | 0.41 (0.31 to 0.52) | 0.29 (0.22 to 0.38) | 0.43 (0.32 to 0.56) | 0.29 (0.07 to 0.5) | 0.009 |
| Saint Lucia | 11.66 (9.82 to 13.80) | 13.50 (11.36 to 16.02) | 32.21 (24.80 to 41.02) | 13.07 (10.08 to 16.55) | -0.06 (-0.45 to 0.33) | 0.756 | 0.44 (0.36 to 0.53) | 0.52 (0.42 to 0.63) | 1.23 (0.94 to 1.57) | 0.50 (0.39 to 0.64) | -0.07 (-0.41 to 0.27) | 0.686 |
| Saint Vincent and the Grenadines | 7.83 (6.51 to 9.26) | 11.22 (9.31 to 13.26) | 19.76 (16.12 to 24.05) | 13.47 (10.96 to 16.42) | 0.58 (0.3 to 0.86) | 0 | 0.30 (0.25 to 0.36) | 0.42 (0.35 to 0.51) | 0.74 (0.60 to 0.91) | 0.51 (0.41 to 0.62) | 0.56 (0.27 to 0.86) | 0 |
| Samoa | 13.05 (10.10 to 16.22) | 14.28 (11.14 to 17.61) | 21.21 (16.04 to 27.93) | 13.62 (10.39 to 17.87) | -0.15 (-0.3 to 0) | 0.058 | 0.46 (0.37 to 0.57) | 0.54 (0.43 to 0.66) | 0.75 (0.58 to 0.98) | 0.51 (0.39 to 0.67) | -0.19 (-0.33 to -0.05) | 0.008 |
| San Marino | 12.26 (9.70 to 15.32) | 36.83 (29.38 to 45.85) | 11.57 (7.13 to 17.03) | 18.04 (10.81 to 26.86) | -2.49 (-2.89 to -2.08) | 0 | 0.53 (0.42 to 0.67) | 1.51 (1.19 to 1.91) | 0.53 (0.33 to 0.76) | 0.73 (0.46 to 1.07) | -2.61 (-2.82 to -2.4) | 0 |
| Sao Tome and Principe | 0.45 (0.33 to 0.58) | 0.67 (0.50 to 0.85) | 1.48 (1.06 to 2.09) | 1.17 (0.84 to 1.62) | 1.83 (1.65 to 2.01) | 0 | 0.02 (0.01 to 0.02) | 0.03 (0.02 to 0.03) | 0.05 (0.04 to 0.07) | 0.04 (0.03 to 0.06) | 1.86 (1.65 to 2.07) | 0 |
| Saudi Arabia | 218.84 (153.95 to 288.26) | 3.21 (2.25 to 4.25) | 1939.89 (1406.09 to 2540.06) | 7.15 (5.28 to 9.32) | 2.61 (2.48 to 2.74) | 0 | 7.13 (5.02 to 9.37) | 0.12 (0.08 to 0.16) | 57.87 (42.59 to 75.53) | 0.27 (0.20 to 0.35) | 2.63 (2.48 to 2.77) | 0 |
| Senegal | 124.36 (92.00 to 161.60) | 3.50 (2.59 to 4.56) | 320.99 (217.52 to 457.52) | 3.65 (2.50 to 5.17) | -0.05 (-0.16 to 0.05) | 0.309 | 4.09 (3.04 to 5.31) | 0.12 (0.09 to 0.16) | 10.52 (7.19 to 14.69) | 0.13 (0.09 to 0.18) | 0.1 (-0.12 to 0.31) | 0.394 |
| Serbia | 4518.89 (3575.90 to 5996.44) | 37.26 (29.55 to 48.84) | 6264.84 (4726.70 to 8088.95) | 41.55 (31.14 to 53.84) | 0.36 (0.19 to 0.52) | 0 | 157.20 (123.60 to 206.43) | 1.36 (1.08 to 1.79) | 245.42 (184.73 to 313.32) | 1.53 (1.15 to 1.96) | 0.42 (0.27 to 0.58) | 0 |
| Seychelles | 14.01 (11.41 to 16.84) | 25.15 (20.43 to 30.28) | 28.18 (22.95 to 33.79) | 22.46 (18.26 to 26.70) | -0.39 (-0.66 to -0.12) | 0.005 | 0.53 (0.43 to 0.64) | 0.94 (0.77 to 1.13) | 1.03 (0.84 to 1.23) | 0.88 (0.71 to 1.06) | -0.3 (-0.67 to 0.08) | 0.12 |
| Sierra Leone | 71.93 (53.44 to 93.61) | 3.40 (2.52 to 4.42) | 162.79 (112.46 to 228.26) | 3.87 (2.71 to 5.34) | 0.42 (0.24 to 0.6) | 0 | 2.60 (1.97 to 3.35) | 0.13 (0.10 to 0.17) | 5.47 (3.84 to 7.52) | 0.14 (0.10 to 0.19) | 0.34 (0.21 to 0.47) | 0 |
| Singapore | 384.12 (336.49 to 442.38) | 16.02 (14.06 to 18.54) | 828.17 (706.16 to 969.86) | 9.28 (7.94 to 10.85) | -1.64 (-2.15 to -1.12) | 0 | 13.76 (12.07 to 15.89) | 0.62 (0.53 to 0.71) | 34.01 (28.86 to 40.22) | 0.39 (0.33 to 0.46) | -1.46 (-2.68 to -0.23) | 0.021 |
| Slovakia | 2684.74 (2138.13 to 3390.99) | 45.69 (36.35 to 57.69) | 3449.23 (2642.77 to 4514.28) | 37.92 (29.06 to 49.66) | -0.59 (-0.82 to -0.36) | 0 | 93.80 (75.10 to 116.69) | 1.57 (1.26 to 1.96) | 130.85 (102.93 to 169.43) | 1.38 (1.08 to 1.79) | -0.41 (-0.75 to -0.06) | 0.021 |
| Slovenia | 786.91 (677.29 to 904.35) | 32.04 (27.39 to 36.76) | 1085.91 (859.21 to 1331.39) | 27.75 (22.08 to 34.01) | -0.58 (-1.32 to 0.16) | 0.124 | 27.95 (23.66 to 32.46) | 1.13 (0.96 to 1.31) | 44.31 (35.01 to 55.21) | 1.05 (0.83 to 1.30) | -0.34 (-0.99 to 0.31) | 0.306 |
| Solomon Islands | 15.45 (8.86 to 22.20) | 9.87 (5.82 to 14.08) | 56.70 (40.26 to 79.63) | 14.00 (9.89 to 19.58) | 1.14 (0.98 to 1.29) | 0 | 0.51 (0.30 to 0.74) | 0.36 (0.22 to 0.51) | 1.82 (1.28 to 2.54) | 0.50 (0.36 to 0.69) | 1.03 (0.89 to 1.18) | 0 |
| Somalia | 110.03 (61.12 to 185.38) | 4.02 (2.31 to 6.56) | 187.45 (96.03 to 307.01) | 2.63 (1.38 to 4.27) | -1.36 (-1.42 to -1.29) | 0 | 3.56 (2.02 to 5.84) | 0.15 (0.09 to 0.25) | 6.12 (3.21 to 10.01) | 0.10 (0.05 to 0.16) | -1.38 (-1.44 to -1.32) | 0 |
| South Africa | 4296.35 (3538.75 to 5638.99) | 19.19 (15.69 to 25.58) | 8718.77 (7306.84 to 10178.30) | 17.17 (14.44 to 20.08) | -0.47 (-1.1 to 0.17) | 0.15 | 145.13 (117.50 to 196.06) | 0.70 (0.56 to 0.95) | 297.36 (247.96 to 348.16) | 0.62 (0.52 to 0.73) | -0.47 (-1.11 to 0.18) | 0.157 |
| South Sudan | 159.96 (96.10 to 249.35) | 5.98 (3.62 to 9.29) | 204.34 (118.36 to 312.47) | 4.73 (2.77 to 7.22) | -0.76 (-0.86 to -0.65) | 0 | 6.00 (3.70 to 9.29) | 0.24 (0.15 to 0.36) | 7.05 (4.13 to 10.80) | 0.19 (0.11 to 0.28) | -0.73 (-0.88 to -0.58) | 0 |
| Spain | 17043.69 (15227.20 to 18928.17) | 33.19 (29.79 to 36.58) | 22189.77 (19361.26 to 25422.16) | 26.89 (23.59 to 30.72) | -0.71 (-1 to -0.43) | 0 | 671.37 (596.05 to 748.30) | 1.25 (1.11 to 1.39) | 907.78 (781.64 to 1059.88) | 1.01 (0.88 to 1.16) | -0.7 (-0.93 to -0.47) | 0 |
| Sri Lanka | 599.48 (488.16 to 726.28) | 5.23 (4.26 to 6.33) | 1009.24 (599.14 to 1501.65) | 3.60 (2.15 to 5.36) | -1.13 (-1.94 to -0.31) | 0.007 | 21.92 (17.92 to 26.45) | 0.21 (0.17 to 0.26) | 39.28 (23.52 to 57.59) | 0.14 (0.09 to 0.21) | -1.25 (-1.85 to -0.65) | 0 |
| Sudan | 370.59 (237.59 to 579.10) | 3.73 (2.38 to 5.77) | 1248.46 (759.60 to 1976.32) | 5.80 (3.53 to 9.12) | 1.44 (1.38 to 1.5) | 0 | 13.13 (8.27 to 20.16) | 0.14 (0.09 to 0.22) | 44.39 (27.10 to 69.06) | 0.23 (0.14 to 0.36) | 1.62 (1.55 to 1.69) | 0 |
| Suriname | 54.02 (42.72 to 64.71) | 19.68 (15.65 to 23.62) | 137.27 (97.39 to 186.45) | 20.33 (14.41 to 27.38) | 0.19 (-0.32 to 0.7) | 0.473 | 1.87 (1.49 to 2.25) | 0.72 (0.57 to 0.87) | 4.77 (3.37 to 6.47) | 0.73 (0.51 to 0.98) | 0.13 (-0.37 to 0.62) | 0.618 |
| Sweden | 5541.88 (4828.69 to 6287.87) | 40.39 (35.39 to 45.57) | 5623.94 (4585.61 to 6780.80) | 28.35 (23.34 to 33.75) | -1.15 (-1.72 to -0.59) | 0 | 253.75 (217.48 to 291.06) | 1.68 (1.45 to 1.92) | 283.36 (226.41 to 349.28) | 1.28 (1.03 to 1.55) | -0.9 (-1.45 to -0.35) | 0.001 |
| Switzerland | 2592.89 (2256.31 to 2990.07) | 27.00 (23.58 to 30.96) | 4569.83 (3930.64 to 5311.86) | 27.24 (23.48 to 31.44) | -0.09 (-0.77 to 0.6) | 0.804 | 111.01 (95.45 to 130.55) | 1.08 (0.94 to 1.26) | 222.88 (187.77 to 262.11) | 1.21 (1.03 to 1.41) | 0.27 (-0.29 to 0.82) | 0.346 |
| Syrian Arab Republic | 615.35 (442.28 to 799.92) | 10.78 (7.74 to 13.94) | 1968.17 (1320.35 to 2761.97) | 13.31 (8.94 to 18.65) | 0.62 (0.31 to 0.94) | 0 | 21.42 (15.24 to 27.68) | 0.41 (0.29 to 0.54) | 70.69 (47.23 to 98.93) | 0.54 (0.36 to 0.73) | 0.76 (0.46 to 1.07) | 0 |
| Taiwan (Province of China) | 3733.64 (3357.25 to 4108.42) | 21.60 (19.47 to 23.69) | 10541.12 (8958.95 to 12148.21) | 25.76 (21.94 to 29.39) | 0.72 (0.46 to 0.98) | 0 | 129.57 (116.20 to 141.91) | 0.80 (0.72 to 0.88) | 416.55 (355.18 to 480.70) | 0.99 (0.84 to 1.14) | 0.76 (0.14 to 1.38) | 0.015 |
| Tajikistan | 167.61 (112.11 to 243.23) | 5.74 (3.82 to 8.42) | 208.50 (130.62 to 309.72) | 3.07 (1.93 to 4.53) | -2.08 (-2.39 to -1.78) | 0 | 0.21 (0.14 to 0.32) | 0.11 (0.07 to 0.16) | 0.12 (0.07 to 0.17) | 0.07 (0.04 to 0.10) | -1.93 (-2.5 to -1.37) | 0 |
| Thailand | 7325.21 (5793.38 to 9201.80) | 18.99 (14.94 to 23.76) | 19946.15 (14617.45 to 27525.81) | 18.30 (13.40 to 25.14) | -0.09 (-0.29 to 0.11) | 0.385 | 260.01 (205.76 to 325.06) | 0.75 (0.60 to 0.92) | 763.15 (564.41 to 1044.19) | 0.69 (0.52 to 0.94) | -0.22 (-0.43 to -0.01) | 0.04 |
| Timor-Leste | 20.81 (12.33 to 30.46) | 6.38 (3.82 to 9.22) | 69.55 (50.00 to 93.96) | 7.82 (5.61 to 10.53) | 0.66 (0.56 to 0.76) | 0 | 0.67 (0.40 to 0.98) | 0.25 (0.15 to 0.35) | 2.56 (1.83 to 3.41) | 0.30 (0.22 to 0.40) | 0.64 (0.55 to 0.72) | 0 |
| Togo | 51.85 (37.45 to 68.41) | 3.87 (2.79 to 5.12) | 218.67 (148.80 to 302.64) | 4.88 (3.34 to 6.63) | 0.78 (0.63 to 0.93) | 0 | 1.80 (1.30 to 2.39) | 0.15 (0.11 to 0.20) | 7.21 (4.92 to 9.78) | 0.18 (0.12 to 0.24) | 0.63 (0.5 to 0.75) | 0 |
| Tokelau | 0.13 (0.08 to 0.19) | 9.32 (6.04 to 13.51) | 0.18 (0.12 to 0.26) | 12.55 (8.08 to 17.91) | 0.97 (0.91 to 1.03) | 0 | 0.00 (0.00 to 0.01) | 0.35 (0.23 to 0.51) | 0.01 (0.00 to 0.01) | 0.46 (0.30 to 0.66) | 0.91 (0.8 to 1.02) | 0 |
| Tonga | 13.56 (10.33 to 18.30) | 23.54 (17.97 to 31.81) | 22.63 (16.38 to 30.32) | 27.70 (20.09 to 36.93) | 0.51 (0.29 to 0.74) | 0 | 0.52 (0.39 to 0.70) | 0.97 (0.73 to 1.32) | 0.89 (0.66 to 1.18) | 1.12 (0.83 to 1.48) | 0.47 (0.21 to 0.73) | 0 |
| Trinidad and Tobago | 142.26 (123.92 to 164.29) | 16.55 (14.38 to 19.16) | 294.14 (209.21 to 390.65) | 15.00 (10.66 to 19.97) | -0.18 (-0.85 to 0.49) | 0.592 | 5.18 (4.46 to 6.04) | 0.62 (0.53 to 0.72) | 10.99 (7.79 to 14.48) | 0.56 (0.40 to 0.73) | -0.22 (-0.83 to 0.4) | 0.485 |
| Tunisia | 487.46 (375.53 to 615.96) | 9.38 (7.24 to 11.87) | 1650.22 (1139.70 to 2313.46) | 11.95 (8.30 to 16.70) | 0.78 (0.69 to 0.86) | 0 | 19.30 (14.83 to 24.28) | 0.40 (0.31 to 0.50) | 66.41 (46.50 to 92.51) | 0.50 (0.36 to 0.70) | 0.7 (0.54 to 0.85) | 0 |
| Turkey | 13784.16 (10273.90 to 18297.05) | 36.13 (26.96 to 47.66) | 31667.01 (23910.41 to 40168.00) | 32.17 (24.39 to 40.88) | -0.37 (-0.69 to -0.06) | 0.021 | 469.50 (351.13 to 615.75) | 1.33 (1.00 to 1.73) | 1176.78 (884.32 to 1482.77) | 1.23 (0.92 to 1.55) | -0.25 (-0.6 to 0.11) | 0.17 |
| Turkmenistan | 15.00 (12.79 to 17.54) | 0.70 (0.60 to 0.82) | 359.66 (255.21 to 493.44) | 7.73 (5.53 to 10.58) | 8.03 (5.52 to 10.6) | 0 | 0.47 (0.40 to 0.55) | 0.02 (0.02 to 0.03) | 11.48 (8.21 to 15.68) | 0.27 (0.19 to 0.36) | 8.17 (5.66 to 10.74) | 0 |
| Tuvalu | 0.74 (0.58 to 0.93) | 9.84 (7.67 to 12.37) | 1.66 (1.23 to 2.22) | 14.76 (10.99 to 19.62) | 1.32 (1.28 to 1.37) | 0 | 0.03 (0.02 to 0.03) | 0.36 (0.28 to 0.44) | 0.06 (0.04 to 0.08) | 0.53 (0.40 to 0.70) | 1.32 (1.27 to 1.38) | 0 |
| Uganda | 329.03 (242.86 to 450.63) | 4.88 (3.65 to 6.61) | 966.22 (654.99 to 1421.52) | 6.06 (4.17 to 8.65) | 0.66 (0.5 to 0.83) | 0 | 12.13 (9.07 to 16.37) | 0.20 (0.15 to 0.26) | 34.45 (23.84 to 48.75) | 0.24 (0.17 to 0.34) | 0.68 (0.51 to 0.84) | 0 |
| Ukraine | 19345.35 (14988.94 to 26649.91) | 27.56 (21.29 to 38.30) | 21748.52 (14848.14 to 30534.76) | 31.19 (21.21 to 43.98) | 0.42 (-0.53 to 1.38) | 0.384 | 639.53 (499.28 to 862.55) | 0.89 (0.69 to 1.20) | 727.84 (497.05 to 1011.31) | 0.99 (0.68 to 1.39) | 0.43 (-0.42 to 1.29) | 0.323 |
| United Arab Emirates | 134.43 (83.81 to 227.74) | 21.70 (13.18 to 36.15) | 1070.88 (784.91 to 1389.75) | 18.52 (13.66 to 24.52) | -0.55 (-0.99 to -0.11) | 0.015 | 3.92 (2.43 to 6.57) | 0.83 (0.52 to 1.38) | 31.07 (22.91 to 40.44) | 0.85 (0.61 to 1.14) | 0.15 (-0.62 to 0.91) | 0.709 |
| United Kingdom | 38513.50 (34562.14 to 42455.53) | 45.23 (40.79 to 49.52) | 33655.11 (28900.75 to 39458.07) | 27.89 (24.30 to 32.32) | -1.57 (-1.9 to -1.23) | 0 | 1733.33 (1537.15 to 1944.99) | 1.90 (1.69 to 2.12) | 1640.25 (1381.48 to 1974.88) | 1.23 (1.05 to 1.46) | -1.39 (-1.65 to -1.13) | 0 |
| United Republic of Tanzania | 883.50 (634.14 to 1243.34) | 7.68 (5.56 to 10.71) | 1681.31 (1074.25 to 2699.69) | 6.07 (3.97 to 9.57) | -0.77 (-0.85 to -0.68) | 0 | 32.34 (23.47 to 44.39) | 0.31 (0.22 to 0.41) | 60.45 (39.79 to 94.88) | 0.24 (0.16 to 0.38) | -0.76 (-0.92 to -0.6) | 0 |
| United States of America | 123470.76 (110952.40 to 137467.74) | 41.06 (37.03 to 45.46) | 180575.14 (155796.46 to 209997.74) | 32.26 (27.99 to 37.16) | -0.77 (-0.99 to -0.55) | 0 | 5096.21 (4525.93 to 5757.36) | 1.61 (1.44 to 1.81) | 7902.69 (6668.53 to 9411.81) | 1.34 (1.14 to 1.58) | -0.62 (-0.79 to -0.45) | 0 |
| United States Virgin Islands | 8.64 (6.63 to 11.17) | 9.21 (7.09 to 11.95) | 15.73 (10.91 to 22.11) | 9.48 (6.59 to 13.28) | 0.02 (-0.45 to 0.49) | 0.932 | 0.30 (0.23 to 0.39) | 0.35 (0.27 to 0.46) | 0.66 (0.45 to 0.93) | 0.36 (0.25 to 0.51) | 0.02 (-0.47 to 0.51) | 0.95 |
| Uruguay | 1623.20 (1411.09 to 1840.65) | 42.96 (37.56 to 48.60) | 2338.66 (1979.95 to 2688.66) | 48.02 (40.96 to 54.96) | 0.36 (0.19 to 0.52) | 0 | 61.96 (53.36 to 71.36) | 1.59 (1.37 to 1.82) | 93.76 (78.28 to 109.92) | 1.80 (1.51 to 2.09) | 0.42 (0.25 to 0.59) | 0 |
| Uzbekistan | 390.37 (282.58 to 501.98) | 3.18 (2.31 to 4.09) | 1971.80 (1512.13 to 2532.97) | 6.38 (4.92 to 8.17) | 2.29 (1.88 to 2.69) | 0 | 12.58 (8.99 to 16.31) | 0.11 (0.08 to 0.14) | 63.00 (48.85 to 80.17) | 0.22 (0.17 to 0.28) | 2.46 (2.09 to 2.82) | 0 |
| Vanuatu | 5.10 (3.80 to 6.74) | 7.36 (5.57 to 9.67) | 15.53 (11.76 to 19.96) | 7.95 (6.08 to 10.11) | 0.23 (0.01 to 0.45) | 0.043 | 0.18 (0.13 to 0.23) | 0.28 (0.21 to 0.36) | 0.53 (0.41 to 0.68) | 0.30 (0.23 to 0.38) | 0.22 (0.08 to 0.37) | 0.002 |
| Venezuela (Bolivarian Republic of) | 512.56 (451.30 to 578.64) | 4.92 (4.30 to 5.55) | 3564.51 (2454.51 to 4853.56) | 11.32 (7.81 to 15.38) | 2.45 (1.85 to 3.05) | 0 | 18.03 (15.68 to 20.29) | 0.19 (0.16 to 0.21) | 136.36 (94.52 to 183.89) | 0.45 (0.31 to 0.60) | 2.59 (1.96 to 3.22) | 0 |
| Viet Nam | 3048.80 (2258.81 to 4031.51) | 7.36 (5.48 to 9.67) | 10798.73 (7946.30 to 14121.60) | 9.90 (7.36 to 12.78) | 0.99 (0.95 to 1.03) | 0 | 116.29 (87.35 to 152.02) | 0.29 (0.22 to 0.38) | 384.45 (288.29 to 488.04) | 0.38 (0.28 to 0.47) | 0.87 (0.81 to 0.92) | 0 |
| Yemen | 297.20 (174.26 to 470.74) | 5.35 (3.15 to 8.42) | 1122.53 (719.08 to 1589.41) | 7.11 (4.56 to 9.83) | 0.93 (0.76 to 1.1) | 0 | 9.84 (5.78 to 15.40) | 0.19 (0.11 to 0.30) | 38.78 (24.82 to 53.38) | 0.28 (0.18 to 0.38) | 1.16 (1.03 to 1.3) | 0 |
| Zambia | 202.16 (154.74 to 256.23) | 6.92 (5.33 to 8.73) | 490.35 (336.67 to 679.51) | 6.69 (4.63 to 9.28) | -0.11 (-0.24 to 0.01) | 0.066 | 7.61 (5.90 to 9.59) | 0.29 (0.23 to 0.36) | 17.72 (12.31 to 24.60) | 0.28 (0.19 to 0.38) | -0.09 (-0.22 to 0.04) | 0.178 |
| Zimbabwe | 694.67 (533.04 to 873.89) | 16.45 (12.73 to 20.59) | 1628.32 (1233.06 to 2136.71) | 21.23 (16.12 to 27.59) | 0.9 (0.7 to 1.09) | 0 | 26.20 (20.47 to 32.64) | 0.69 (0.54 to 0.87) | 55.98 (42.29 to 72.17) | 0.83 (0.64 to 1.06) | 0.73 (0.53 to 0.94) | 0 |

**Note:** PC, pancreatic cancer; DALYs: disability-adjusted life years; ASDR: age-standardized disability-adjusted life year; ASMR: age-standardized mortality rate; SDI: socio-demographic index; AAPC: average annual percentage changes.

**Supplementary Table 2** Global mortality and DALYs of PC attributable to smoking across age in 2021

| **Age** | **Male** | | **Female** | |
| --- | --- | --- | --- | --- |
|  | **Mortality**  **(/100,000) (95% UI)** | **DALYs**  **(/100,000) (95% UI)** | **Mortality**  **(/100,000) (95% UI)** | **DALYs**  **(/100,000) (95% UI)** |
| 30-34 | 0.07 (0.06 to 0.08) | 4.02 (3.42 to 4.73) | 0.01 (0.01 to 0.01) | 0.37 (0.32 to 0.41) |
| 35-39 | 0.19 (0.16 to 0.22) | 10.15 (8.66 to 11.95) | 0.02 (0.02 to 0.02) | 0.98 (0.88 to 1.09) |
| 40-44 | 0.45 (0.39 to 0.52) | 21.61 (18.63 to 25.13) | 0.05 (0.05 to 0.06) | 2.57 (2.28 to 2.88) |
| 45-49 | 0.94 (0.81 to 1.10) | 40.76 (35.00 to 47.45) | 0.14 (0.12 to 0.16) | 5.92 (5.22 to 6.75) |
| 50-54 | 1.98 (1.69 to 2.32) | 75.99 (64.91 to 89.15) | 0.32 (0.28 to 0.37) | 12.35 (10.90 to 14.20) |
| 55-59 | 3.46 (2.98 to 4.04) | 116.87 (100.77 to 136.12) | 0.69 (0.61 to 0.79) | 23.34 (20.50 to 26.59) |
| 60-64 | 5.25 (4.53 to 6.05) | 152.62 (131.67 to 175.69) | 1.18 (1.03 to 1.35) | 34.33 (30.08 to 39.26) |
| 65-69 | 7.47 (6.36 to 8.68) | 182.99 (155.97 to 212.62) | 1.68 (1.43 to 1.98) | 41.21 (34.93 to 48.33) |
| 70-74 | 9.89 (8.44 to 11.6) | 199.4 (170.18 to 234.02) | 2.36 (1.97 to 2.82) | 47.5 (39.67 to 56.89) |
| 75-79 | 11.14 (9.39 to 13.14) | 179.69 (151.49 to 212.00) | 2.76 (2.23 to 3.35) | 44.34 (35.92 to 53.84) |
| 80-84 | 11.56 (9.56 to 13.98) | 146.09 (120.83 to 176.50) | 2.95 (2.15 to 3.64) | 37.17 (27.06 to 45.81) |
| 85-89 | 14.66 (11.89 to 18.10) | 147.45 (119.49 to 181.16) | 3.77 (2.61 to 4.82) | 37.70 (26.07 to 48.29) |
| 90-94 | 14.82 (11.41 to 18.81) | 129.93 (100.04 to 165.27) | 4.81 (3.25 to 6.42) | 42.10 (28.38 to 56.23) |
| 95+ | 10.98 (7.93 to 15.65) | 89.28 (64.59 to 127.18) | 4.73 (2.88 to 6.83) | 38.53 (23.52 to 55.57) |

**Note:** PC：Pancreatic cancer; DALYs: disability-adjusted life years;

### **Supplementary Table 3** The join-points of ASMR and ASDR by sex in global and different SDI regions from 1990 to 2021

| **Age** | **Measure** | **Location** | **Years Join-point 1**  **APC (95%CI)** | **Years Join-point 2**  **APC (95%CI)** | **Years Join-point 3**  **APC (95%CI)** | **Years Join-point 4**  **APC (95%CI)** | **Years Join-point 5**  **APC (95%CI)** |
| --- | --- | --- | --- | --- | --- | --- | --- |
| Both | ASMR | Global | 1990-1994  -0.19(-0.48 to 0.09) | 1994-1998***  -1.04(-1.47 to -0.61) | 1998-2007***  -0.42(-0.52 to -0.32) | 2007-2010  0.02(-0.9 to 0.95) | 2010-2021***  -0.65(-0.72 to -0.58) |
|  |  | High SDI | 1990-2004***  -0.6806(-0.7379 to -0.6232) | 2004-2009  -0.2269(-0.662 to 0.2101) | 2009-2019***  -0.797(-0.9314 to -0.6623) | 2019-2021*  -1.9588(-3.5263 to -0.3658) | - |
|  |  | High-middle SDI | 1990-1994***  0.8271(0.3623 to 1.2941) | 1994-1999***  -1.0667(-1.4985 to -0.633) | 1999-2004**  0.7208(0.2907 to 1.1528) | 2004-2012  -0.018(-0.2108 to 0.1752) | 2012-2021***  -0.3598(-0.5383 to -0.1811) |
|  |  | Middle SDI | 1990-1995  -0.0618(-0.2734 to 0.1502) | 1995-2006***  -0.7018(-0.7642 to -0.6394) | 2006-2011***  1.054(0.7995 to 1.3092) | 2011-2015  -0.3227(-0.7912 to 0.148) | 2015-2021*  0.2783(0.0677 to 0.4894) |
|  |  | Low-middle SDI | 1990-2010***  0.6794(0.6432 to 0.7157) | 2010-2013  -0.1068(-1.0799 to 0.8758) | 2013-2019***  0.9597(0.7574 to 1.1624) | 2019-2021  -0.0309(-0.9875 to 0.9349) | - |
|  |  | Low SDI | 1990-1995  0.2595(-0.111 to 0.6313) | 1995-2005***  -1.0679(-1.2123 to -0.9234) | 2005-2009  -0.2521(-1.025 to 0.5268) | 2009-2021***  0.6497(0.5635 to 0.7359) | - |
|  | ASDR | Global | 1990-1994  -0.25(-0.57 to 0.08) | 1994-1999***  -1.15(-1.44 to -0.85) | 1999-2011***  -0.42(-0.48 to -0.36) | 2011-2021***  -0.83(-0.92 to -0.73) | - |
|  |  | High SDI | 1990-2004***  -0.7354(-0.7916 to -0.6791) | 2004-2009  -0.3373(-0.7552 to 0.0824) | 2009-2019***  -1.0266(-1.1526 to -0.9005) | 2019-2021**  -2.2003(-3.6904 to -0.6872) | - |
|  |  | High-middle SDI | 1990-1994***  0.859(0.3921 to 1.3281) | 1994-1999***  -1.4322(-1.862 to -1.0005) | 1999-2005**  0.4144(0.1178 to 0.7119) | 2005-2021***  -0.3812(-0.4487 to -0.3137) | - |
|  |  | Middle SDI | 1990-1993  -0.146(-0.7673 to 0.4791) | 1993-2006***  -0.7926(-0.849 to -0.7362) | 2006-2011***  0.9585(0.6658 to 1.252) | 2011-2016*  -0.415(-0.7642 to -0.0647) | 2016-2021  0.2828(-0.0681 to 0.635) |
|  |  | Low-middle SDI | 1990-2010***  0.6131(0.5802 to 0.6459) | 2010-2014  0.0623(-0.3643 to 0.4908) | 2014-2019***  0.9595(0.6948 to 1.2249) | 2019-2021  -0.0372(-0.9089 to 0.8421) | - |
|  |  | Low SDI | 1990-1997  -0.1235(-0.2743 to 0.0276) | 1997-2005***  -1.3299(-1.4701 to -1.1895) | 2005-2010  -0.2973(-0.6197 to 0.0262) | 2010-2021***  0.576(0.5094 to 0.6426) | - |
| Male | ASMR | Global | 1990-1994*  -0.32(-0.61 to -0.04) | 1994-1998***  -1.2(-1.61 to -0.78) | 1998-2007***  -0.42(-0.52 to -0.33) | 2007-2010  0.17(-0.72 to 1.06) | 2010-2021***  -0.45(-0.53 to -0.38) |
|  |  | High SDI | 1990-2004***  -0.9593(-1.0145 to -0.9042) | 2004-2011***  -0.6838(-0.908 to -0.4591) | 2011-2014*  -1.461(-2.8246 to -0.0783) | 2014-2018  -0.5841(-1.3128 to 0.15) | 2018-2021***  -1.592(-2.3534 to -0.8246) |
|  |  | High-middle SDI | 1990-1994  0.5767(-0.0008 to 1.1575) | 1994-1999***  -1.4553(-1.9734 to -0.9344) | 1999-2004*  0.573(0.0639 to 1.0847) | 2004-2021***  -0.1699(-0.2446 to -0.0952) | - |
|  |  | Middle SDI | 1990-2006***  -0.4311(-0.4798 to -0.3823) | 2006-2011***  1.5562(1.1959 to 1.9178) | 2011-2021**  0.2008(0.0703 to 0.3314) | - | - |
|  |  | Low-middle SDI | 1990-2003***  0.7051(0.6284 to 0.7818) | 2003-2009***  1.2612(1.0059 to 1.5172) | 2009-2013  0.4869(-0.0054 to 0.9817) | 2013-2019***  1.1884(0.9867 to 1.3906) | 2019-2021  0.4839(-0.4778 to 1.4549) |
|  |  | Low SDI | 1990-1997  -0.0702(-0.2776 to 0.1377) | 1997-2005***  -1.3265(-1.5228 to -1.1299) | 2005-2011  -0.0114(-0.3244 to 0.3027) | 2011-2021***  0.7968(0.6948 to 0.8988) | - |
|  | ASDR | Global | 1990-1994  -0.24(-0.55 to 0.08) | 1994-1998***  -1.37(-1.81 to -0.93) | 1998-2007***  -0.52(-0.62 to -0.42) | 2007-2010  -0.03(-0.96 to 0.91) | 2010-2021***  -0.65(-0.73 to -0.57) |
|  |  | High SDI | 1990-2004***  -0.979(-1.0397 to -0.9183) | 2004-2009**  -0.721(-1.1703 to -0.2697) | 2009-2021***  -1.2435(-1.3366 to -1.1503) | - | - |
|  |  | High-middle SDI | 1990-1994*  0.7153(0.1563 to 1.2775) | 1994-1999***  -1.7538(-2.2479 to -1.2571) | 1999-2004  0.301(-0.1891 to 0.7935) | 2004-2021***  -0.3524(-0.4252 to -0.2795) | - |
|  |  | Middle SDI | 1990-2002***  -0.6716(-0.7379 to -0.6053) | 2002-2006  -0.3864(-0.8692 to 0.0987) | 2006-2011***  1.3654(1.065 to 1.6667) | 2011-2016  -0.1588(-0.5215 to 0.2051) | 2016-2021  0.354(-0.0198 to 0.7292) |
|  |  | Low-middle SDI | 1990-2003***  0.625(0.5564 to 0.6936) | 2003-2010***  1.1401(0.9717 to 1.3087) | 2010-2013  0.1686(-0.696 to 1.0409) | 2013-2019***  1.0675(0.8896 to 1.2458) | 2019-2021  0.4428(-0.4303 to 1.3235) |
|  |  | Low SDI | 1990-1998*  -0.1121(-0.2217 to -0.0025) | 1998-2002***  -1.7918(-2.2528 to -1.3285) | 2002-2006***  -1.0009(-1.4594 to -0.5403) | 2006-2012  -0.0834(-0.2828 to 0.1163) | 2012-2021***  0.7557(0.6761 to 0.8355) |
| Female | ASMR | Global | 1990-2009***  -0.59(-0.62 to -0.55) | 2009-2019***  -1.28(-1.41 to -1.16) | 2019-2021**  -2.5(-3.94 to -1.03) | - | - |
|  |  | High SDI | 1990-2004***  -0.5174(-0.5905 to -0.4443) | 2004-2009  0.2737(-0.2831 to 0.8337) | 2009-2018***  -0.5928(-0.7925 to -0.3927) | 2018-2021***  -2.2056(-3.1684 to -1.2332) | - |
|  |  | High-middle SDI | 1990-1999***  0.3752(0.2326 to 0.5181) | 1999-2005***  1.0281(0.6912 to 1.3662) | 2005-2010  -0.1327(-0.5821 to 0.3187) | 2010-2021***  -1.1711(-1.2884 to -1.0537) | - |
|  |  | Middle SDI | 1990-1995***  0.8073(0.5511 to 1.0641) | 1995-2000***  -0.9272(-1.2472 to -0.6062) | 2000-2015***  -1.9791(-2.0286 to -1.9295) | 2015-2021  -0.0483(-0.2812 to 0.1851) | - |
|  |  | Low-middle SDI | 1990-2001***  1.3905(1.285 to 1.4961) | 2001-2007  -0.1589(-0.4449 to 0.1279) | 2007-2014***  -1.1789(-1.38 to -0.9774) | 2014-2019  0.3818(-0.0037 to 0.7688) | 2019-2021*  -1.6774(-2.9111 to -0.428) |
|  |  | Low SDI | 1990-1994*  0.8338(0.1631 to 1.5091) | 1994-1998  -0.7556(-1.8051 to 0.3051) | 1998-2010  0.0072(-0.1309 to 0.1455) | 2010-2013**  3.0245(1.0542 to 5.0332) | 2013-2021  0.0048(-0.1998 to 0.2099) |
|  | ASDR | Global | 1990-2002***  -0.71(-0.79 to -0.63) | 2002-2009***  -0.49(-0.72 to -0.25) | 2009-2018***  -1.38(-1.54 to -1.22) | 2018-2021***  -2.27(-3.04 to -1.5) | - |
|  |  | High SDI | 1990-2003***  -0.5494(-0.6443 to -0.4544) | 2003-2009  0.2314(-0.2119 to 0.6768) | 2009-2018***  -0.7438(-0.9687 to -0.5184) | 2018-2021***  -2.4317(-3.5188 to -1.3323) | - |
|  |  | High-middle SDI | 1990-1999  -0.0081(-0.1622 to 0.1463) | 1999-2005***  0.929(0.5715 to 1.2877) | 2005-2009  0.0841(-0.6775 to 0.8516) | 2009-2018***  -1.0141(-1.2069 to -0.8209) | 2018-2021**  -1.8802(-2.9431 to -0.8056) |
|  |  | Middle SDI | 1990-1994***  0.7261(0.3676 to 1.0858) | 1994-2000***  -0.9911(-1.2133 to -0.7684) | 2000-2003***  -2.1087(-3.0594 to -1.1486) | 2003-2015***  -1.7993(-1.8686 to -1.7299) | 2015-2021  -0.0507(-0.2787 to 0.1779) |
|  |  | Low-middle SDI | 1990-2001***  1.2644(1.1728 to 1.3561) | 2001-2007*  -0.3028(-0.546 to -0.0589) | 2007-2014***  -1.2057(-1.3715 to -1.0397) | 2014-2018*  0.648(0.1473 to 1.1512) | 2018-2021**  -0.8376(-1.3505 to -0.322) |
|  |  | Low SDI | 1990-1992  0.8155(-1.0881 to 2.7558) | 1992-2009***  -0.3545(-0.4244 to -0.2846) | 2009-2015***  1.2494(0.8657 to 1.6345) | 2015-2021  0.059(-0.2166 to 0.3354) | - |

**Note:** PC, pancreatic cancer; SDI: socio-demographic index; ASMR: age-standardized mortality rate; ASDR: age-standardized disability-adjusted life rate; APC: Annual percentage change; * *P* < 0.05, ** *P* < 0.01, *** *P* < 0.001

### **Supplementary Table 4** The global predictive analysis of PC attributable to smoking in ASDR and ASMR from 1990 to 2035

| **year** | **ASDR (/100,000) (95% UI)** | **ASMR (/100,000) (95% UI)** |
| --- | --- | --- |
| 1990 | 52.47（52.36 to 52.57) | 2.06（2.04 to 2.08) |
| 1991 | 52.24（52.14 to 52.34) | 2.05（2.04 to 2.07) |
| 1992 | 52.12（52.02 to 52.21) | 2.05（2.03 to 2.06) |
| 1993 | 52.14（52.04 to 52.24) | 2.04（2.03 to 2.06) |
| 1994 | 51.86（51.76 to 51.95) | 2.03（2.02 to 2.05) |
| 1995 | 51.44（51.35 to 51.53) | 2.02（2.00 to 2.03) |
| 1996 | 50.70（50.61 to 50.79) | 2.00（1.98 to 2.01) |
| 1997 | 49.98（49.89 to 50.07) | 1.98（1.96 to 1.99) |
| 1998 | 49.48（49.39 to 49.57) | 1.96（1.95 to 1.97) |
| 1999 | 49.13（49.05 to 49.22) | 1.95（1.93 to 1.96) |
| 2000 | 49.05（48.96 to 49.13) | 1.94（1.93 to 1.95) |
| 2001 | 48.59（48.50 to 48.67) | 1.93（1.91 to 1.94) |
| 2002 | 48.23（48.14 to 48.31) | 1.92（1.90 to 1.93) |
| 2003 | 48.05（47.97 to 48.14) | 1.91（1.90 to 1.92) |
| 2004 | 47.88（47.80 to 47.96) | 1.91（1.89 to 1.92) |
| 2005 | 47.94（47.86 to 48.02) | 1.90（1.89 to 1.92) |
| 2006 | 47.41（47.33 to 47.49) | 1.89（1.88 to 1.90) |
| 2007 | 47.17（47.09 to 47.25) | 1.89（1.87 to 1.90) |
| 2008 | 47.19（47.11 to 47.27) | 1.89（1.87 to 1.90) |
| 2009 | 46.98（46.90 to 47.06) | 1.88（1.87 to 1.89) |
| 2010 | 46.99（46.91 to 47.06) | 1.88（1.87 to 1.89) |
| 2011 | 46.68（46.61 to 46.76) | 1.87（1.86 to 1.89) |
| 2012 | 46.40（46.33 to 46.47) | 1.86（1.85 to 1.88) |
| 2013 | 45.78（45.71 to 45.85) | 1.85（1.84 to 1.86) |
| 2014 | 45.17（45.10 to 45.24) | 1.83（1.82 to 1.84) |
| 2015 | 44.84（44.77 to 44.90) | 1.82（1.81 to 1.83) |
| 2016 | 44.68（44.61 to 44.75) | 1.81（1.80 to 1.82) |
| 2017 | 44.34（44.28 to 44.41) | 1.80（1.79 to 1.81) |
| 2018 | 44.13（44.07 to 44.20) | 1.79（1.78 to 1.81) |
| 2019 | 43.91（43.84 to 43.97) | 1.79（1.77 to 1.80) |
| 2020 | 43.14（43.08 to 43.20) | 1.76（1.75 to 1.77) |
| 2021 | 42.79（42.72 to 42.85) | 1.75（1.74 to 1.76) |
| 2022 | 42.12（41.36 to 42.88) | 1.73（1.70 to 1.76) |
| 2023 | 41.55（40.40 to 42.70) | 1.71（1.67 to 1.76) |
| 2024 | 40.98（39.34 to 42.62) | 1.70（1.63 to 1.77) |
| 2025 | 40.40（38.21 to 42.59) | 1.68（1.59 to 1.77) |
| 2026 | 39.82（37.02 to 42.62) | 1.66（1.54 to 1.78) |
| 2027 | 39.24（35.79 to 42.69) | 1.64（1.50 to 1.79) |
| 2028 | 38.67（34.54 to 42.81) | 1.62（1.45 to 1.79) |
| 2029 | 38.11（33.27 to 42.95) | 1.60（1.40 to 1.80) |
| 2030 | 37.55（31.98 to 43.12) | 1.58（1.35 to 1.81) |
| 2031 | 36.98（30.67 to 43.30) | 1.56（1.30 to 1.83) |
| 2032 | 36.44（29.36 to 43.52) | 1.54（1.25 to 1.84) |
| 2033 | 35.93（28.07 to 43.78) | 1.52（1.19 to 1.85) |
| 2034 | 35.43（26.77 to 44.08) | 1.50（1.14 to 1.86) |
| 2035 | 34.93（25.48 to 44.38) | 1.48（1.09 to 1.88) |

### Note: PC, pancreatic cancer; ASDR: age-standardized disability-adjusted life rate; ASMR: age-standardized mortality rate.

### **Supplementary Table 5** The predictive analysis of PC attributable to smoking in ASDR and ASMR across regions with different SDI levels from 1990 to 2035

| **location** | **High SDI** | | **High-middle SDI** | | **Middle SDI** | | **Low-middle SDI** | | **Low SDI** | |
| --- | --- | --- | --- | --- | --- | --- | --- | --- | --- | --- |
| **year** | **ASDR (/100,000) (95% UI)** | **ASMR (/100,000) (95% UI)** | **ASDR (/100,000) (95% UI)** | **ASMR (/100,000) (95% UI)** | **ASDR (/100,000) (95% UI)** | **ASMR (/100,000) (95% UI)** | **ASDR (/100,000) (95% UI)** | **ASMR (/100,000) (95% UI)** | **ASDR (/100,000) (95% UI)** | **ASMR (/100,000) (95% UI)** |
| 1990 | 112.8（112.41 to 113.19) | 5.55（5.46 to 5.64) | 83.84（83.55 to 84.13) | 3.30（3.24 to 3.35) | 42.98（42.78 to 43.18) | 1.67（1.63 to 1.71) | 9.52（9.43 to 9.62) | 0.38（0.36 to 0.39) | 1.98（1.94 to 2.01) | 0.08（0.07 to 0.08) |
| 1991 | 110.48（110.10 to 110.85) | 5.41（5.35 to 5.48) | 83.46（83.18 to 83.75) | 3.28（3.23 to 3.33) | 43.04（42.84 to 43.24) | 1.66（1.63 to 1.69) | 9.50（9.42 to 9.59) | 0.38（0.36 to 0.39) | 1.97（1.94 to 2.00) | 0.08（0.07 to 0.08) |
| 1992 | 108.54（108.18 to 108.90) | 5.29（5.23 to 5.36) | 83.48（83.20 to 83.76) | 3.27（3.23 to 3.32) | 42.97（42.77 to 43.16) | 1.66（1.63 to 1.68) | 9.51（9.43 to 9.59) | 0.38（0.37 to 0.39) | 1.95（1.93 to 1.98) | 0.08（0.07 to 0.08) |
| 1993 | 106.88（106.53 to 107.23) | 5.18（5.12 to 5.24) | 84.11（83.84 to 84.38) | 3.27（3.22 to 3.31) | 42.79（42.60 to 42.99) | 1.65（1.62 to 1.67) | 9.52（9.43 to 9.60) | 0.38（0.37 to 0.39) | 1.95（1.92 to 1.97) | 0.07（0.07 to 0.08) |
| 1994 | 104.64（104.30 to 104.98) | 5.06（5.00 to 5.11) | 83.74（83.48 to 84.01) | 3.25（3.20 to 3.29) | 42.50（42.31 to 42.68) | 1.64（1.61 to 1.66) | 9.55（9.47 to 9.63) | 0.38（0.37 to 0.39) | 1.93（1.91 to 1.96) | 0.07（0.07 to 0.08) |
| 1995 | 102.51（102.18 to 102.84) | 4.93（4.88 to 4.98) | 82.60（82.33 to 82.86) | 3.20（3.16 to 3.24) | 42.54（42.36 to 42.73) | 1.63（1.61 to 1.65) | 9.63（9.55 to 9.71) | 0.38（0.37 to 0.39) | 1.91（1.88 to 1.94) | 0.07（0.07 to 0.08) |
| 1996 | 99.92（99.60 to 100.24) | 4.81（4.76 to 4.86) | 80.68（80.42 to 80.93) | 3.14（3.10 to 3.18) | 42.27（42.09 to 42.45) | 1.62（1.60 to 1.64) | 9.73（9.65 to 9.81) | 0.38（0.37 to 0.39) | 1.89（1.87 to 1.92) | 0.07（0.07 to 0.08) |
| 1997 | 97.94（97.63 to 98.25) | 4.70（4.65 to 4.75) | 78.89（78.64 to 79.14) | 3.08（3.04 to 3.12) | 41.60（41.42 to 41.77) | 1.60（1.58 to 1.63) | 9.77（9.69 to 9.85) | 0.38（0.37 to 0.39) | 1.88（1.86 to 1.91) | 0.07（0.07 to 0.07) |
| 1998 | 96.77（96.47 to 97.07) | 4.62（4.57 to 4.67) | 77.01（76.76 to 77.25) | 3.03（2.99 to 3.06) | 41.33（41.15 to 41.50) | 1.59（1.57 to 1.61) | 9.78（9.70 to 9.86) | 0.38（0.37 to 0.39) | 1.86（1.84 to 1.89) | 0.07（0.07 to 0.07) |
| 1999 | 95.86（95.57 to 96.16) | 4.54（4.50 to 4.59) | 75.42（75.18 to 75.66) | 2.98（2.94 to 3.02) | 40.95（40.78 to 41.12) | 1.58（1.56 to 1.60) | 9.74（9.67 to 9.82) | 0.38（0.37 to 0.39) | 1.82（1.80 to 1.85) | 0.07（0.07 to 0.07) |
| 2000 | 94.31（94.02 to 94.59) | 4.45（4.41 to 4.50) | 75.84（75.61 to 76.08) | 2.98（2.94 to 3.02) | 40.73（40.56 to 40.90) | 1.57（1.55 to 1.59) | 9.81（9.74 to 9.89) | 0.38（0.37 to 0.39) | 1.79（1.76 to 1.81) | 0.07（0.07 to 0.07) |
| 2001 | 91.94（91.67 to 92.22) | 4.35（4.31 to 4.40) | 75.35（75.12 to 75.58) | 2.96（2.93 to 3.00) | 40.39（40.23 to 40.56) | 1.56（1.53 to 1.58) | 9.85（9.77 to 9.92) | 0.38（0.38 to 0.39) | 1.75（1.73 to 1.78) | 0.07（0.06 to 0.07) |
| 2002 | 89.96（89.69 to 90.23) | 4.26（4.22 to 4.31) | 74.85（74.62 to 75.08) | 2.95（2.91 to 2.99) | 40.06（39.90 to 40.22) | 1.55（1.52 to 1.57) | 9.87（9.79 to 9.94) | 0.38（0.38 to 0.39) | 1.72（1.70 to 1.75) | 0.07（0.06 to 0.07) |
| 2003 | 88.56（88.30 to 88.83) | 4.19（4.15 to 4.23) | 74.67（74.45 to 74.89) | 2.95（2.91 to 2.98) | 39.70（39.54 to 39.86) | 1.54（1.51 to 1.56) | 9.90（9.83 to 9.98) | 0.39（0.38 to 0.39) | 1.69（1.67 to 1.72) | 0.07（0.06 to 0.07) |
| 2004 | 87.07（86.81 to 87.32) | 4.12（4.08 to 4.16) | 74.06（73.84 to 74.28) | 2.93（2.89 to 2.97) | 39.67（39.51 to 39.83) | 1.53（1.51 to 1.55) | 9.91（9.83 to 9.98) | 0.39（0.38 to 0.40) | 1.66（1.64 to 1.68) | 0.06（0.06 to 0.07) |
| 2005 | 86.43（86.18 to 86.69) | 4.06（4.02 to 4.10) | 74.17（73.95 to 74.38) | 2.92（2.89 to 2.96) | 39.49（39.33 to 39.64) | 1.53（1.51 to 1.55) | 9.97（9.90 to 10.04) | 0.39（0.38 to 0.40) | 1.64（1.62 to 1.66) | 0.06（0.06 to 0.07) |
| 2006 | 84.81（84.57 to 85.06) | 3.99（3.95 to 4.03) | 72.32（72.11 to 72.53) | 2.87（2.83 to 2.90) | 39.13（38.98 to 39.28) | 1.52（1.50 to 1.54) | 10.02（9.95 to 10.09) | 0.39（0.38 to 0.40) | 1.62（1.60 to 1.64) | 0.06（0.06 to 0.07) |
| 2007 | 83.24（83.01 to 83.48) | 3.91（3.88 to 3.95) | 70.97（70.76 to 71.17) | 2.83（2.79 to 2.86) | 39.30（39.16 to 39.45) | 1.53（1.51 to 1.55) | 10.05（9.98 to 10.12) | 0.39（0.38 to 0.40) | 1.60（1.58 to 1.63) | 0.06（0.06 to 0.06) |
| 2008 | 81.61（81.38 to 81.84) | 3.84（3.80 to 3.88) | 70.45（70.25 to 70.65) | 2.81（2.77 to 2.84) | 39.85（39.70 to 40.00) | 1.54（1.52 to 1.56) | 10.09（10.02 to 10.16) | 0.39（0.38 to 0.40) | 1.59（1.57 to 1.61) | 0.06（0.06 to 0.06) |
| 2009 | 79.84（79.62 to 80.06) | 3.76（3.72 to 3.80) | 69.41（69.22 to 69.60) | 2.77（2.74 to 2.81) | 40.07（39.93 to 40.22) | 1.55（1.53 to 1.57) | 10.07（10.00 to 10.13) | 0.39（0.38 to 0.40) | 1.58（1.56 to 1.60) | 0.06（0.06 to 0.06) |
| 2010 | 78.29（78.08 to 78.51) | 3.68（3.64 to 3.72) | 69.31（69.12 to 69.51) | 2.76（2.73 to 2.79) | 40.70（40.55 to 40.84) | 1.57（1.55 to 1.59) | 10.1（10.03 to 10.17) | 0.39（0.38 to 0.40) | 1.58（1.56 to 1.60) | 0.06（0.06 to 0.06) |
| 2011 | 76.37（76.17 to 76.58) | 3.59（3.56 to 3.63) | 68.49（68.30 to 68.68) | 2.73（2.70 to 2.76) | 40.91（40.77 to 41.06) | 1.58（1.56 to 1.60) | 10.14（10.07 to 10.21) | 0.39（0.38 to 0.40) | 1.58（1.56 to 1.60) | 0.06（0.06 to 0.06) |
| 2012 | 74.42（74.22 to 74.62) | 3.50（3.47 to 3.53) | 67.88（67.70 to 68.06) | 2.70（2.67 to 2.74) | 41.05（40.91 to 41.19) | 1.58（1.56 to 1.60) | 10.1（10.04 to 10.17) | 0.39（0.38 to 0.40) | 1.58（1.56 to 1.60) | 0.06（0.06 to 0.06) |
| 2013 | 72.30（72.10 to 72.49) | 3.41（3.38 to 3.44) | 66.37（66.19 to 66.54) | 2.66（2.63 to 2.69) | 40.85（40.71 to 40.99) | 1.58（1.56 to 1.60) | 10.02（9.95 to 10.08) | 0.39（0.38 to 0.40) | 1.58（1.56 to 1.60) | 0.06（0.06 to 0.06) |
| 2014 | 70.09（69.91 to 70.28) | 3.32（3.29 to 3.35) | 65.13（64.96 to 65.30) | 2.61（2.58 to 2.64) | 40.57（40.44 to 40.71) | 1.58（1.56 to 1.60) | 10.01（9.95 to 10.07) | 0.39（0.38 to 0.40) | 1.58（1.56 to 1.60) | 0.06（0.06 to 0.06) |
| 2015 | 68.69（68.51 to 68.87) | 3.25（3.21 to 3.28) | 64.41（64.24 to 64.58) | 2.58（2.55 to 2.61) | 40.62（40.49 to 40.76) | 1.58（1.56 to 1.60) | 10.17（10.10 to 10.23) | 0.39（0.38 to 0.40) | 1.60（1.58 to 1.62) | 0.06（0.06 to 0.06) |
| 2016 | 67.49（67.31 to 67.66) | 3.18（3.15 to 3.21) | 64.10（63.93 to 64.26) | 2.55（2.53 to 2.58) | 40.74（40.61 to 40.87) | 1.58（1.56 to 1.60) | 10.31（10.25 to 10.37) | 0.39（0.39 to 0.40) | 1.62（1.60 to 1.63) | 0.06（0.06 to 0.06) |
| 2017 | 65.86（65.69 to 66.03) | 3.10（3.07 to 3.13) | 63.39（63.23 to 63.56) | 2.52（2.49 to 2.55) | 40.86（40.73 to 40.99) | 1.58（1.56 to 1.60) | 10.38（10.32 to 10.44) | 0.40（0.39 to 0.40) | 1.63（1.61 to 1.65) | 0.06（0.06 to 0.06) |
| 2018 | 64.10（63.94 to 64.26) | 3.02（2.99 to 3.05) | 63.06（62.90 to 63.22) | 2.50（2.47 to 2.52) | 41.11（40.98 to 41.24) | 1.59（1.57 to 1.61) | 10.48（10.42 to 10.55) | 0.40（0.39 to 0.40) | 1.65（1.63 to 1.67) | 0.06（0.06 to 0.06) |
| 2019 | 62.27（62.11 to 62.42) | 2.93（2.91 to 2.96) | 62.74（62.58 to 62.90) | 2.48（2.45 to 2.50) | 41.52（41.39 to 41.65) | 1.60（1.58 to 1.62) | 10.54（10.48 to 10.61) | 0.40（0.39 to 0.41) | 1.67（1.65 to 1.69) | 0.06（0.06 to 0.06) |
| 2020 | 59.58（59.43 to 59.73) | 2.83（2.81 to 2.86) | 61.69（61.53 to 61.84) | 2.44（2.42 to 2.47) | 41.76（41.63 to 41.88) | 1.61（1.59 to 1.62) | 10.62（10.55 to 10.68) | 0.40（0.39 to 0.41) | 1.69（1.67 to 1.71) | 0.06（0.06 to 0.07) |
| 2021 | 58.25（58.10 to 58.40) | 2.75（2.72 to 2.78) | 60.95（60.80 to 61.10) | 2.41（2.38 to 2.43) | 41.88（41.75 to 42.00) | 1.61（1.59 to 1.63) | 10.65（10.59 to 10.72) | 0.40（0.39 to 0.41) | 1.72（1.69 to 1.74) | 0.06（0.06 to 0.07) |
| 2022 | 55.42（53.51 to 57.32) | 2.66（2.60 to 2.73) | 57.94（55.39 to 60.48) | 2.36（2.28 to 2.43) | 40.75（39.20 to 42.30) | 1.61（1.56 to 1.65) | 10.5（10.19 to 10.82) | 0.40（0.39 to 0.42) | 1.74（1.69 to 1.79) | 0.06（0.06 to 0.07) |
| 2023 | 53.40（51.03 to 55.76) | 2.57（2.48 to 2.67) | 56.97（53.71 to 60.23) | 2.32（2.21 to 2.43) | 40.86（38.88 to 42.84) | 1.62（1.55 to 1.68) | 10.52（10.10 to 10.95) | 0.40（0.38 to 0.43) | 1.77（1.69 to 1.84) | 0.06（0.06 to 0.07) |
| 2024 | 51.56（48.62 to 54.50) | 2.49（2.37 to 2.62) | 56.13（51.94 to 60.31) | 2.29（2.14 to 2.44) | 40.99（38.45 to 43.53) | 1.62（1.53 to 1.71) | 10.58（10.01 to 11.14) | 0.41（0.38 to 0.43) | 1.8（1.70 to 1.90) | 0.06（0.06 to 0.07) |
| 2025 | 49.92（46.32 to 53.52) | 2.42（2.26 to 2.57) | 55.36（50.10 to 60.62) | 2.26（2.07 to 2.46) | 41.13（37.91 to 44.35) | 1.63（1.51 to 1.75) | 10.68（9.95 to 11.41) | 0.41（0.37 to 0.44) | 1.84（1.70 to 1.97) | 0.07（0.06 to 0.07) |
| 2026 | 48.30（43.98 to 52.62) | 2.34（2.15 to 2.54) | 54.51（48.06 to 60.97) | 2.23（1.99 to 2.47) | 41.28（37.29 to 45.26) | 1.64（1.48 to 1.79) | 10.78（9.86 to 11.70) | 0.41（0.37 to 0.46) | 1.88（1.71 to 2.05) | 0.07（0.06 to 0.08) |
| 2027 | 46.62（41.54 to 51.70) | 2.27（2.04 to 2.50) | 53.61（45.86 to 61.36) | 2.2（1.90 to 2.49) | 41.42（36.58 to 46.26) | 1.64（1.45 to 1.83) | 10.85（9.71 to 11.98) | 0.42（0.36 to 0.47) | 1.92（1.70 to 2.13) | 0.07（0.05 to 0.08) |
| 2028 | 44.96（39.11 to 50.80) | 2.19（1.92 to 2.46) | 52.81（43.70 to 61.92) | 2.16（1.81 to 2.51) | 41.58（35.81 to 47.36) | 1.65（1.42 to 1.88) | 10.91（9.54 to 12.28) | 0.42（0.35 to 0.48) | 1.96（1.69 to 2.22) | 0.07（0.05 to 0.08) |
| 2029 | 43.45（36.85 to 50.04) | 2.12（1.81 to 2.43) | 52.13（41.59 to 62.66) | 2.14（1.73 to 2.54) | 41.77（35.00 to 48.54) | 1.66（1.38 to 1.93) | 11.00（9.39 to 12.62) | 0.42（0.35 to 0.50) | 2.00（1.68 to 2.32) | 0.07（0.05 to 0.09) |
| 2030 | 42.09（34.73 to 49.45) | 2.06（1.71 to 2.40) | 51.49（39.48 to 63.50) | 2.11（1.64 to 2.58) | 41.96（34.12 to 49.79) | 1.66（1.34 to 1.98) | 11.14（9.25 to 13.03) | 0.42（0.34 to 0.51) | 2.05（1.67 to 2.43) | 0.07（0.05 to 0.09) |
| 2031 | 40.78（32.66 to 48.90) | 1.99（1.61 to 2.37) | 50.79（37.27 to 64.32) | 2.08（1.56 to 2.61) | 42.14（33.18 to 51.11) | 1.67（1.30 to 2.04) | 11.28（9.10 to 13.46) | 0.43（0.33 to 0.53) | 2.10（1.66 to 2.55) | 0.07（0.05 to 0.10) |
| 2032 | 39.45（30.57 to 48.33) | 1.93（1.51 to 2.34) | 50.06（34.98 to 65.13) | 2.05（1.46 to 2.64) | 42.33（32.18 to 52.48) | 1.68（1.26 to 2.09) | 11.38（8.89 to 13.88) | 0.43（0.32 to 0.54) | 2.16（1.64 to 2.67) | 0.07（0.05 to 0.10) |
| 2033 | 38.15（28.54 to 47.77) | 1.86（1.41 to 2.31) | 49.42（32.74 to 66.10) | 2.02（1.37 to 2.68) | 42.53（31.13 to 53.94) | 1.69（1.22 to 2.15) | 11.48（8.66 to 14.31) | 0.44（0.31 to 0.56) | 2.21（1.61 to 2.80) | 0.08（0.05 to 0.11) |
| 2034 | 36.97（26.62 to 47.31) | 1.80（1.32 to 2.29) | 48.89（30.55 to 67.22) | 2.00（1.28 to 2.72) | 42.75（30.03 to 55.48) | 1.69（1.17 to 2.22) | 11.61（8.43 to 14.78) | 0.44（0.30 to 0.58) | 2.26（1.59 to 2.94) | 0.08（0.04 to 0.11) |
| 2035 | 35.88（24.82 to 46.94) | 1.75（1.23 to 2.27) | 48.36（28.34 to 68.39) | 1.98（1.20 to 2.76) | 42.97（28.87 to 57.07) | 1.70（1.12 to 2.28) | 11.76（8.21 to 15.31) | 0.44（0.29 to 0.60) | 2.32（1.55 to 3.09) | 0.08（0.04 to 0.12) |

**Note:** PC, pancreatic cancer; SDI: socio-demographic index; ASDR: age-standardized disability-adjusted life rate; ASMR: age-standardized mortality rate.

### **Supplementary Table 6** The predictive analysis of ASDR and ASMR of PC attributable to smoking in the seven geographic super-regions with the highest disease burden in 2021, from 1990 to 2035

| **location** | **Central Europe** | | **High-income North America** | | **Western Europe** | | **Eastern Europe** | | **High-income Asia Pacific** | | **Southern Latin America** | | **East Asia** | |
| --- | --- | --- | --- | --- | --- | --- | --- | --- | --- | --- | --- | --- | --- | --- |
| **year** | **ASDR (/100,000) (95% UI)** | **ASMR (/100,000) (95% UI)** | **ASDR (/100,000) (95% UI)** | **ASMR (/100,000) (95% UI)** | **ASDR (/100,000) (95% UI)** | **ASMR (/100,000) (95% UI)** | **ASDR (/100,000) (95% UI)** | **ASMR (/100,000) (95% UI)** | **ASDR (/100,000) (95% UI)** | **ASMR (/100,000) (95% UI)** | **ASDR (/100,000) (95% UI)** | **ASMR (/100,000) (95% UI)** | **ASDR (/100,000) (95% UI)** | **ASMR (/100,000) (95% UI)** |
| 1990 | 93.68 (93.00 to 94.36) | 3.29 (3.19 to 3.38) | 86.39 (85.94 to 86.84) | 3.42 (3.35 to 3.49) | 81.43 (81.08 to 81.77) | 3.24 (3.19 to 3.30) | 75.80 (75.33 to 76.28) | 2.44 (2.36 to 2.51) | 89.31 (88.74 to 89.88) | 3.61 (3.52 to 3.70) | 81.61 (80.51 to 82.70) | 2.96 (2.81 to 3.11) | 55.36 (55.14 to 55.59) | 2.07 (2.03 to 2.11) |
| 1991 | 93.79 (93.15 to 94.43) | 3.30 (3.22 to 3.38) | 86.05 (85.62 to 86.48) | 3.40 (3.35 to 3.46) | 80.37 (80.05 to 80.70) | 3.21 (3.17 to 3.26) | 76.96 (76.49 to 77.43) | 2.52 (2.46 to 2.58) | 88.77 (88.22 to 89.32) | 3.58 (3.51 to 3.65) | 81.54 (80.58 to 82.50) | 2.97 (2.85 to 3.09) | 55.59 (55.37 to 55.81) | 2.07 (2.03 to 2.10) |
| 1992 | 93.91 (93.28 to 94.55) | 3.31 (3.25 to 3.38) | 85.69 (85.27 to 86.12) | 3.39 (3.34 to 3.44) | 79.74 (79.42 to 80.07) | 3.19 (3.14 to 3.23) | 80.11 (79.63 to 80.58) | 2.63 (2.57 to 2.69) | 87.92 (87.38 to 88.46) | 3.54 (3.48 to 3.60) | 81.40 (80.46 to 82.35) | 2.99 (2.88 to 3.09) | 55.37 (55.16 to 55.58) | 2.06 (2.03 to 2.10) |
| 1993 | 94.20 (93.57 to 94.84) | 3.33 (3.26 to 3.40) | 85.28 (84.86 to 85.70) | 3.37 (3.32 to 3.42) | 79.09 (78.77 to 79.41) | 3.16 (3.12 to 3.20) | 87.02 (86.53 to 87.51) | 2.77 (2.71 to 2.83) | 87.61 (87.08 to 88.14) | 3.50 (3.44 to 3.56) | 81.41 (80.47 to 82.35) | 3.00 (2.91 to 3.10) | 54.81 (54.60 to 55.02) | 2.05 (2.02 to 2.08) |
| 1994 | 95.11 (94.48 to 95.75) | 3.35 (3.28 to 3.42) | 84.63 (84.21 to 85.04) | 3.35 (3.30 to 3.40) | 78.56 (78.24 to 78.88) | 3.14 (3.10 to 3.18) | 90.10 (89.60 to 90.60) | 2.85 (2.78 to 2.91) | 86.66 (86.14 to 87.18) | 3.46 (3.40 to 3.52) | 81.26 (80.33 to 82.20) | 3.02 (2.92 to 3.11) | 54.05 (53.84 to 54.25) | 2.03 (2.00 to 2.07) |
| 1995 | 96.21 (95.57 to 96.85) | 3.37 (3.30 to 3.43) | 83.41 (83.00 to 83.83) | 3.32 (3.28 to 3.37) | 77.93 (77.62 to 78.25) | 3.11 (3.07 to 3.15) | 89.33 (88.83 to 89.83) | 2.83 (2.77 to 2.90) | 85.38 (84.87 to 85.89) | 3.41 (3.35 to 3.47) | 81.71 (80.78 to 82.64) | 3.04 (2.94 to 3.13) | 54.09 (53.88 to 54.29) | 2.03 (2.00 to 2.06) |
| 1996 | 96.36 (95.72 to 97.00) | 3.38 (3.31 to 3.45) | 82.19 (81.78 to 82.59) | 3.30 (3.25 to 3.35) | 77.27 (76.96 to 77.58) | 3.09 (3.05 to 3.13) | 85.39 (84.90 to 85.89) | 2.75 (2.69 to 2.81) | 83.80 (83.30 to 84.30) | 3.35 (3.30 to 3.41) | 82.93 (82.00 to 83.86) | 3.06 (2.96 to 3.15) | 53.71 (53.51 to 53.91) | 2.02 (1.99 to 2.05) |
| 1997 | 97.43 (96.79 to 98.07) | 3.39 (3.33 to 3.46) | 81.31 (80.91 to 81.71) | 3.28 (3.23 to 3.32) | 76.94 (76.64 to 77.25) | 3.08 (3.04 to 3.12) | 81.25 (80.78 to 81.73) | 2.64 (2.58 to 2.70) | 81.85 (81.36 to 82.33) | 3.30 (3.24 to 3.35) | 84.91 (83.97 to 85.86) | 3.08 (2.99 to 3.18) | 53.21 (53.01 to 53.41) | 2.01 (1.98 to 2.04) |
| 1998 | 96.57 (95.94 to 97.20) | 3.39 (3.33 to 3.46) | 81.19 (80.79 to 81.58) | 3.27 (3.22 to 3.31) | 77.02 (76.71 to 77.33) | 3.07 (3.03 to 3.11) | 78.05 (77.58 to 78.51) | 2.56 (2.50 to 2.62) | 81.51 (81.03 to 81.99) | 3.26 (3.20 to 3.31) | 85.53 (84.59 to 86.47) | 3.10 (3.00 to 3.19) | 52.71 (52.52 to 52.91) | 2.00 (1.97 to 2.03) |
| 1999 | 95.59 (94.97 to 96.22) | 3.39 (3.33 to 3.46) | 82.48 (82.08 to 82.87) | 3.27 (3.22 to 3.31) | 77.13 (76.82 to 77.43) | 3.06 (3.02 to 3.10) | 76.24 (75.78 to 76.70) | 2.52 (2.46 to 2.58) | 80.83 (80.36 to 81.30) | 3.21 (3.16 to 3.26) | 85.82 (84.88 to 86.76) | 3.11 (3.02 to 3.20) | 52.45 (52.26 to 52.64) | 2.00 (1.97 to 2.03) |
| 2000 | 95.14 (94.52 to 95.76) | 3.39 (3.33 to 3.46) | 81.76 (81.37 to 82.15) | 3.26 (3.21 to 3.30) | 77.15 (76.84 to 77.46) | 3.06 (3.02 to 3.10) | 79.21 (78.74 to 79.68) | 2.56 (2.50 to 2.62) | 79.24 (78.78 to 79.71) | 3.16 (3.10 to 3.21) | 85.25 (84.32 to 86.17) | 3.12 (3.02 to 3.21) | 52.97 (52.78 to 53.16) | 2.02 (1.99 to 2.05) |
| 2001 | 95.12 (94.50 to 95.74) | 3.41 (3.34 to 3.47) | 81.21 (80.82 to 81.60) | 3.24 (3.20 to 3.29) | 76.81 (76.51 to 77.12) | 3.04 (3.00 to 3.08) | 80.09 (79.62 to 80.57) | 2.61 (2.55 to 2.67) | 76.95 (76.50 to 77.41) | 3.10 (3.04 to 3.15) | 84.39 (83.48 to 85.31) | 3.12 (3.03 to 3.21) | 52.83 (52.65 to 53.02) | 2.02 (1.99 to 2.05) |
| 2002 | 95.39 (94.77 to 96.01) | 3.42 (3.36 to 3.49) | 80.54 (80.16 to 80.92) | 3.23 (3.18 to 3.27) | 76.44 (76.14 to 76.75) | 3.03 (2.99 to 3.07) | 81.55 (81.07 to 82.02) | 2.66 (2.60 to 2.72) | 74.90 (74.46 to 75.35) | 3.04 (2.99 to 3.09) | 84.75 (83.84 to 85.66) | 3.12 (3.03 to 3.21) | 52.54 (52.36 to 52.73) | 2.02 (1.99 to 2.05) |
| 2003 | 96.78 (96.16 to 97.40) | 3.45 (3.39 to 3.52) | 79.87 (79.49 to 80.24) | 3.21 (3.17 to 3.26) | 76.22 (75.92 to 76.52) | 3.02 (2.99 to 3.06) | 82.83 (82.35 to 83.30) | 2.71 (2.65 to 2.77) | 74.44 (74.01 to 74.88) | 3.00 (2.95 to 3.05) | 84.81 (83.91 to 85.72) | 3.12 (3.03 to 3.21) | 52.44 (52.26 to 52.62) | 2.03 (2.00 to 2.06) |
| 2004 | 97.43 (96.80 to 98.05) | 3.48 (3.41 to 3.54) | 79.22 (78.85 to 79.59) | 3.20 (3.15 to 3.24) | 75.83 (75.54 to 76.13) | 3.02 (2.98 to 3.05) | 83.29 (82.81 to 83.77) | 2.74 (2.68 to 2.80) | 73.88 (73.45 to 74.32) | 2.97 (2.92 to 3.01) | 83.80 (82.90 to 84.69) | 3.11 (3.02 to 3.20) | 52.83 (52.65 to 53.01) | 2.04 (2.02 to 2.07) |
| 2005 | 98.46 (97.83 to 99.08) | 3.51 (3.44 to 3.57) | 79.03 (78.66 to 79.39) | 3.18 (3.14 to 3.23) | 76.21 (75.92 to 76.51) | 3.02 (2.98 to 3.06) | 86.52 (86.04 to 87.01) | 2.77 (2.71 to 2.84) | 73.24 (72.81 to 73.66) | 2.93 (2.88 to 2.98) | 83.67 (82.78 to 84.55) | 3.09 (3.00 to 3.18) | 52.95 (52.77 to 53.13) | 2.06 (2.03 to 2.08) |
| 2006 | 98.67 (98.05 to 99.29) | 3.53 (3.46 to 3.59) | 78.52 (78.16 to 78.88) | 3.17 (3.12 to 3.21) | 76.76 (76.47 to 77.06) | 3.02 (2.99 to 3.06) | 82.34 (81.87 to 82.82) | 2.72 (2.66 to 2.78) | 71.58 (71.16 to 71.99) | 2.89 (2.84 to 2.94) | 83.32 (82.45 to 84.20) | 3.08 (2.99 to 3.17) | 52.64 (52.47 to 52.81) | 2.05 (2.03 to 2.08) |
| 2007 | 99.66 (99.04 to 100.28) | 3.54 (3.48 to 3.61) | 77.64 (77.29 to 77.99) | 3.14 (3.10 to 3.19) | 76.99 (76.70 to 77.28) | 3.03 (2.99 to 3.07) | 79.57 (79.10 to 80.03) | 2.68 (2.62 to 2.74) | 70.30 (69.89 to 70.71) | 2.85 (2.81 to 2.90) | 83.36 (82.49 to 84.23) | 3.06 (2.97 to 3.14) | 53.12 (52.95 to 53.29) | 2.07 (2.05 to 2.10) |
| 2008 | 100.10 (99.48 to 100.72) | 3.55 (3.49 to 3.62) | 76.83 (76.48 to 77.17) | 3.12 (3.08 to 3.16) | 76.93 (76.64 to 77.22) | 3.03 (3.00 to 3.07) | 80.42 (79.96 to 80.89) | 2.67 (2.61 to 2.73) | 69.68 (69.27 to 70.08) | 2.82 (2.78 to 2.87) | 82.11 (81.25 to 82.96) | 3.02 (2.94 to 3.11) | 54.01 (53.84 to 54.18) | 2.11 (2.08 to 2.14) |
| 2009 | 99.75 (99.13 to 100.36) | 3.55 (3.48 to 3.61) | 76.06 (75.73 to 76.40) | 3.10 (3.05 to 3.14) | 76.65 (76.36 to 76.94) | 3.03 (2.99 to 3.06) | 78.38 (77.93 to 78.84) | 2.65 (2.59 to 2.71) | 69.06 (68.66 to 69.46) | 2.80 (2.75 to 2.84) | 80.71 (79.87 to 81.55) | 2.99 (2.90 to 3.07) | 54.99 (54.82 to 55.16) | 2.15 (2.13 to 2.18) |
| 2010 | 98.51 (97.90 to 99.12) | 3.53 (3.46 to 3.59) | 74.93 (74.60 to 75.27) | 3.07 (3.03 to 3.11) | 76.50 (76.21 to 76.79) | 3.02 (2.98 to 3.06) | 79.58 (79.12 to 80.04) | 2.65 (2.59 to 2.71) | 68.44 (68.04 to 68.83) | 2.77 (2.73 to 2.82) | 78.14 (77.32 to 78.95) | 2.94 (2.86 to 3.03) | 56.29 (56.12 to 56.45) | 2.20 (2.17 to 2.23) |
| 2011 | 97.01 (96.40 to 97.61) | 3.51 (3.44 to 3.57) | 74.07 (73.75 to 74.40) | 3.04 (3.00 to 3.08) | 75.68 (75.40 to 75.96) | 3.01 (2.97 to 3.04) | 77.37 (76.92 to 77.83) | 2.62 (2.56 to 2.68) | 67.92 (67.53 to 68.32) | 2.74 (2.70 to 2.79) | 76.93 (76.13 to 77.73) | 2.91 (2.82 to 2.99) | 57.18 (57.01 to 57.34) | 2.24 (2.21 to 2.27) |
| 2012 | 96.66 (96.06 to 97.26) | 3.48 (3.42 to 3.55) | 73.39 (73.07 to 73.71) | 3.02 (2.98 to 3.06) | 75.20 (74.92 to 75.48) | 3.00 (2.96 to 3.03) | 76.86 (76.41 to 77.30) | 2.60 (2.54 to 2.66) | 66.82 (66.44 to 67.21) | 2.71 (2.67 to 2.75) | 76.20 (75.41 to 76.99) | 2.87 (2.79 to 2.95) | 57.52 (57.35 to 57.68) | 2.25 (2.22 to 2.28) |
| 2013 | 94.94 (94.35 to 95.53) | 3.45 (3.39 to 3.52) | 72.03 (71.72 to 72.34) | 2.98 (2.94 to 3.02) | 74.80 (74.52 to 75.08) | 2.98 (2.95 to 3.02) | 76.28 (75.84 to 76.72) | 2.59 (2.54 to 2.65) | 65.50 (65.12 to 65.88) | 2.68 (2.63 to 2.72) | 74.94 (74.17 to 75.72) | 2.84 (2.76 to 2.92) | 57.09 (56.92 to 57.25) | 2.24 (2.22 to 2.27) |
| 2014 | 94.74 (94.15 to 95.33) | 3.43 (3.36 to 3.49) | 71.70 (71.39 to 72.01) | 2.96 (2.92 to 3.00) | 73.71 (73.43 to 73.98) | 2.97 (2.93 to 3.00) | 77.12 (76.68 to 77.57) | 2.61 (2.55 to 2.66) | 63.70 (63.33 to 64.07) | 2.64 (2.60 to 2.69) | 74.01 (73.24 to 74.77) | 2.80 (2.72 to 2.88) | 56.25 (56.09 to 56.40) | 2.22 (2.19 to 2.24) |
| 2015 | 94.31 (93.72 to 94.89) | 3.40 (3.33 to 3.46) | 71.19 (70.89 to 71.50) | 2.94 (2.90 to 2.98) | 73.91 (73.64 to 74.18) | 2.96 (2.93 to 3.00) | 76.72 (76.28 to 77.16) | 2.61 (2.56 to 2.67) | 61.93 (61.56 to 62.29) | 2.61 (2.57 to 2.66) | 73.30 (72.55 to 74.05) | 2.77 (2.69 to 2.85) | 55.98 (55.82 to 56.13) | 2.21 (2.18 to 2.23) |
| 2016 | 92.12 (91.54 to 92.69) | 3.36 (3.29 to 3.42) | 71.07 (70.76 to 71.37) | 2.92 (2.88 to 2.95) | 73.93 (73.65 to 74.20) | 2.96 (2.92 to 2.99) | 77.80 (77.36 to 78.24) | 2.64 (2.58 to 2.70) | 61.79 (61.43 to 62.15) | 2.60 (2.56 to 2.64) | 72.79 (72.05 to 73.53) | 2.74 (2.66 to 2.81) | 55.98 (55.83 to 56.13) | 2.21 (2.18 to 2.23) |
| 2017 | 91.14 (90.57 to 91.71) | 3.32 (3.26 to 3.38) | 70.03 (69.73 to 70.33) | 2.88 (2.84 to 2.92) | 73.25 (72.98 to 73.52) | 2.94 (2.90 to 2.98) | 77.88 (77.44 to 78.32) | 2.66 (2.60 to 2.72) | 60.97 (60.62 to 61.33) | 2.58 (2.53 to 2.62) | 72.10 (71.36 to 72.83) | 2.70 (2.62 to 2.78) | 56.26 (56.11 to 56.41) | 2.21 (2.19 to 2.24) |
| 2018 | 90.30 (89.73 to 90.87) | 3.28 (3.22 to 3.35) | 68.47 (68.18 to 68.77) | 2.84 (2.80 to 2.88) | 72.62 (72.35 to 72.88) | 2.91 (2.87 to 2.94) | 79.57 (79.13 to 80.02) | 2.69 (2.63 to 2.75) | 60.80 (60.44 to 61.15) | 2.56 (2.52 to 2.60) | 72.10 (71.37 to 72.83) | 2.66 (2.59 to 2.74) | 56.60 (56.45 to 56.75) | 2.22 (2.20 to 2.25) |
| 2019 | 88.66 (88.10 to 89.22) | 3.24 (3.17 to 3.30) | 67.56 (67.27 to 67.84) | 2.80 (2.76 to 2.84) | 71.27 (71.00 to 71.53) | 2.86 (2.82 to 2.89) | 80.68 (80.24 to 81.13) | 2.70 (2.64 to 2.76) | 60.00 (59.64 to 60.35) | 2.53 (2.49 to 2.58) | 71.00 (70.28 to 71.72) | 2.62 (2.54 to 2.70) | 57.10 (56.96 to 57.25) | 2.24 (2.21 to 2.26) |
| 2020 | 86.46 (85.91 to 87.01) | 3.19 (3.12 to 3.26) | 66.09 (65.81 to 66.37) | 2.75 (2.71 to 2.79) | 67.59 (67.34 to 67.84) | 2.78 (2.74 to 2.81) | 78.73 (78.29 to 79.17) | 2.66 (2.61 to 2.72) | 58.34 (58.00 to 58.69) | 2.50 (2.46 to 2.55) | 68.80 (68.10 to 69.50) | 2.57 (2.48 to 2.66) | 57.52 (57.38 to 57.67) | 2.26 (2.23 to 2.28) |
| 2021 | 85.62 (85.05 to 86.20) | 3.14 (3.06 to 3.22) | 65.79 (65.50 to 66.08) | 2.72 (2.68 to 2.77) | 66.45 (66.19 to 66.71) | 2.71 (2.67 to 2.76) | 77.35 (76.91 to 77.79) | 2.62 (2.55 to 2.69) | 58.30 (57.95 to 58.65) | 2.49 (2.43 to 2.54) | 65.56 (64.80 to 66.31) | 2.51 (2.41 to 2.62) | 57.67 (57.52 to 57.81) | 2.26 (2.24 to 2.29) |
| 2022 | 83.07 (80.87 to 85.27) | 3.09 (2.96 to 3.22) | 64.32 (62.86 to 65.79) | 2.68 (2.60 to 2.76) | 64.63 (63.22 to 66.04) | 2.65 (2.57 to 2.73) | 74.78 (70.06 to 79.49) | 2.57 (2.40 to 2.74) | 58.58 (56.85 to 60.31) | 2.46 (2.38 to 2.55) | 62.75 (60.52 to 64.98) | 2.47 (2.32 to 2.61) | 56.90 (54.53 to 59.27) | 2.27 (2.19 to 2.35) |
| 2023 | 81.36 (78.19 to 84.52) | 3.04 (2.87 to 3.21) | 63.26 (61.05 to 65.47) | 2.64 (2.53 to 2.75) | 62.54 (60.22 to 64.86) | 2.59 (2.47 to 2.70) | 73.51 (65.10 to 81.91) | 2.52 (2.24 to 2.81) | 57.92 (55.56 to 60.29) | 2.44 (2.32 to 2.55) | 60.22 (56.69 to 63.76) | 2.42 (2.23 to 2.60) | 57.07 (54.04 to 60.10) | 2.28 (2.16 to 2.39) |
| 2024 | 79.61 (75.26 to 83.96) | 2.98 (2.77 to 3.20) | 62.22 (59.09 to 65.36) | 2.60 (2.46 to 2.75) | 60.46 (57.06 to 63.87) | 2.52 (2.36 to 2.68) | 72.24 (59.35 to 85.13) | 2.48 (2.05 to 2.90) | 57.20 (54.03 to 60.36) | 2.41 (2.26 to 2.56) | 57.79 (52.76 to 62.82) | 2.37 (2.14 to 2.60) | 57.25 (53.35 to 61.15) | 2.29 (2.13 to 2.44) |
| 2025 | 77.83 (72.16 to 83.50) | 2.93 (2.66 to 3.20) | 61.18 (57.01 to 65.35) | 2.56 (2.38 to 2.75) | 58.43 (53.85 to 63.00) | 2.45 (2.25 to 2.65) | 70.93 (53.03 to 88.83) | 2.43 (1.85 to 3.01) | 56.43 (52.35 to 60.51) | 2.38 (2.19 to 2.58) | 55.45 (48.84 to 62.06) | 2.32 (2.04 to 2.60) | 57.44 (52.50 to 62.39) | 2.30 (2.09 to 2.50) |
| 2026 | 76.03 (68.93 to 83.12) | 2.87 (2.54 to 3.20) | 60.14 (54.85 to 65.44) | 2.52 (2.29 to 2.75) | 56.45 (50.63 to 62.26) | 2.38 (2.13 to 2.63) | 69.55 (46.26 to 92.84) | 2.38 (1.64 to 3.13) | 55.70 (50.61 to 60.79) | 2.36 (2.12 to 2.59) | 53.21 (44.98 to 61.43) | 2.27 (1.95 to 2.60) | 57.65 (51.51 to 63.79) | 2.31 (2.04 to 2.57) |
| 2027 | 74.23 (65.64 to 82.82) | 2.81 (2.42 to 3.20) | 59.11 (52.62 to 65.60) | 2.48 (2.21 to 2.76) | 54.50 (47.41 to 61.58) | 2.32 (2.02 to 2.62) | 68.16 (39.18 to 97.13) | 2.34 (1.42 to 3.25) | 54.99 (48.81 to 61.18) | 2.33 (2.04 to 2.61) | 51.06 (41.20 to 60.93) | 2.23 (1.85 to 2.61) | 57.88 (50.42 to 65.34) | 2.32 (1.99 to 2.65) |
| 2028 | 72.45 (62.32 to 82.57) | 2.75 (2.30 to 3.20) | 58.10 (50.35 to 65.84) | 2.44 (2.12 to 2.76) | 52.57 (44.21 to 60.93) | 2.25 (1.90 to 2.60) | 66.80 (31.91 to 101.69) | 2.29 (1.20 to 3.38) | 54.27 (46.94 to 61.60) | 2.30 (1.96 to 2.63) | 49.02 (37.52 to 60.51) | 2.18 (1.75 to 2.62) | 58.13 (49.23 to 67.03) | 2.33 (1.94 to 2.72) |
| 2029 | 70.67 (58.99 to 82.36) | 2.69 (2.18 to 3.20) | 57.12 (48.09 to 66.16) | 2.40 (2.03 to 2.77) | 50.68 (41.05 to 60.30) | 2.19 (1.79 to 2.59) | 65.45 (24.47 to 106.42) | 2.24 (0.97 to 3.52) | 53.50 (44.99 to 62.01) | 2.27 (1.88 to 2.65) | 47.06 (33.95 to 60.16) | 2.14 (1.65 to 2.63) | 58.40 (47.95 to 68.86) | 2.34 (1.88 to 2.81) |
| 2030 | 68.90 (55.65 to 82.14) | 2.63 (2.06 to 3.20) | 56.16 (45.80 to 66.52) | 2.36 (1.94 to 2.78) | 48.83 (37.96 to 59.70) | 2.12 (1.67 to 2.57) | 64.07 (16.92 to 111.22) | 2.19 (0.73 to 3.66) | 52.70 (42.98 to 62.42) | 2.23 (1.80 to 2.67) | 45.18 (30.51 to 59.86) | 2.09 (1.54 to 2.64) | 58.71 (46.60 to 70.81) | 2.35 (1.81 to 2.90) |
| 2031 | 67.13 (52.32 to 81.94) | 2.57 (1.93 to 3.20) | 55.23 (43.51 to 66.94) | 2.32 (1.85 to 2.79) | 47.05 (34.96 to 59.13) | 2.06 (1.56 to 2.55) | 62.64 (9.30 to 115.98) | 2.15 (0.50 to 3.79) | 51.93 (40.97 to 62.89) | 2.20 (1.72 to 2.69) | 43.40 (27.19 to 59.61) | 2.05 (1.44 to 2.65) | 59.03 (45.16 to 72.90) | 2.37 (1.74 to 2.99) |
| 2032 | 65.40 (49.02 to 81.78) | 2.50 (1.81 to 3.20) | 54.31 (41.22 to 67.41) | 2.28 (1.76 to 2.80) | 45.30 (32.02 to 58.57) | 1.99 (1.45 to 2.54) | 61.19 (1.67 to 120.70) | 2.10 (0.26 to 3.93) | 51.20 (38.96 to 63.44) | 2.17 (1.63 to 2.71) | 41.70 (24.00 to 59.40) | 2.00 (1.35 to 2.66) | 59.39 (43.65 to 75.13) | 2.38 (1.67 to 3.09) |
| 2033 | 63.72 (45.79 to 81.66) | 2.44 (1.69 to 3.20) | 53.44 (38.94 to 67.94) | 2.24 (1.67 to 2.81) | 43.59 (29.18 to 58.00) | 1.93 (1.34 to 2.52) | 59.75 (-5.92 to 125.43) | 2.05 (0.03 to 4.07) | 50.47 (36.93 to 64.01) | 2.14 (1.54 to 2.73) | 40.08 (20.94 to 59.21) | 1.96 (1.25 to 2.68) | 59.79 (42.06 to 77.51) | 2.39 (1.59 to 3.19) |
| 2034 | 62.08 (42.61 to 81.55) | 2.38 (1.57 to 3.19) | 52.61 (36.68 to 68.54) | 2.20 (1.58 to 2.83) | 41.92 (26.42 to 57.42) | 1.87 (1.24 to 2.50) | 58.33 (-13.45 to 130.11) | 2.00 (-0.20 to 4.20) | 49.71 (34.87 to 64.54) | 2.10 (1.46 to 2.75) | 38.52 (18.01 to 59.03) | 1.92 (1.15 to 2.69) | 60.22 (40.41 to 80.04) | 2.41 (1.51 to 3.30) |
| 2035 | 60.45 (39.48 to 81.43) | 2.32 (1.45 to 3.19) | 51.82 (34.45 to 69.18) | 2.16 (1.49 to 2.84) | 40.30 (23.77 to 56.84) | 1.81 (1.13 to 2.49) | 56.90 (-20.87 to 134.67) | 1.95 (-0.43 to 4.34) | 48.93 (32.79 to 65.06) | 2.07 (1.37 to 2.77) | 37.03 (15.20 to 58.86) | 1.88 (1.05 to 2.71) | 60.70 (38.68 to 82.72) | 2.42 (1.43 to 3.42) |

**Note:** PC, pancreatic cancer; SDI: socio-demographic index; ASDR: age-standardized disability-adjusted life rate; ASMR: age-standardized mortality rate.
